# Synthesis of pyrazolo[5′,1′:2,3]imidazo[1,5-*c*]quinazolin-6(5*H*)-ones and molecular docking study of their affinity against the COVID-19 main protease†

**DOI:** 10.1039/d2ra03179e

**Published:** 2022-07-08

**Authors:** Shaghayegh Saeedi, Abbas Rahmati, Zahra Chavoshpour-Natanzi

**Affiliations:** Department of Chemistry, University of Isfahan P. O. Box 81746-73441 Isfahan Iran a.rahmati@sci.ui.ac.ir +98 31 37934943

## Abstract

A novel series of fused pyrazolo[5′,1′:2,3]imidazo[1,5-*c*]quinazolin-6(5*H*)-ones were synthesized and their affinity against the COVID-19 main protease was investigated using molecular docking study and compared to that of some used standard clinical drugs. These compounds were obtained in good to excellent yields from 63 to 91% in the presence of 30 mol% catalyst in ethanol at reflux for 2 h through an efficient one-pot three-component reaction including an intramolecular rearrangement and a cyclization through intramolecular nucleophilic reaction. The results of *in silico* studies showed that electronegativity, resonance effects, hydrophobic interaction, halogen and hydrogen bonding had significant effects on the performance of these compounds as an inhibitor ligand. Also, these results indicated the proper affinity of these compounds against the COVID-19 main protease with excellent binding energies (especially 4r = −8.77, 4q = −8.73 and 4m = −8.63) in comparison to remdesivir, chloroquine, hydroxychloroquine, molnupiravir and nirmatrelvir drugs.

## Abstract 2

A novel series of fused pyrazolo[5′,1′:2,3]imidazo[1,5-*c*]quinazolin-6(5*H*)-ones were synthesized and their affinity against the COVID-19 main protease was investigated using molecular docking study and compared to that of some used clinical drugs.

## Graphical abstract

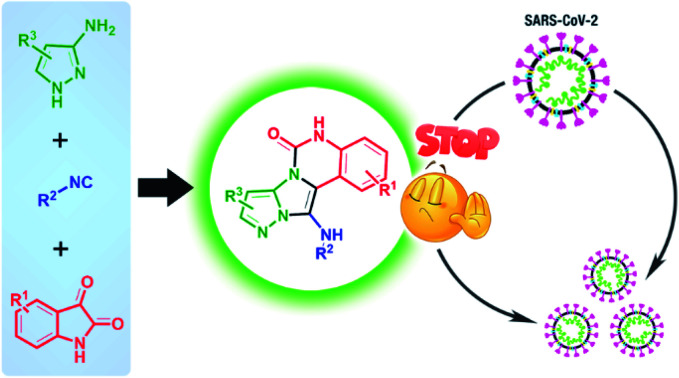

## 1. Introduction

Synthesis of heterocycles containing nitrogen atoms is very essential in organic chemistry and pharmaceutical sciences.^1,2^ In 2014, Vitaku and coworkers revealed that heterocycles are present in about 60% of the small-molecule drugs approved by the US FDA.^3^ They showed that five- and six-membered heterocycles, such as imidazole, pyrazole, and pyrimidine, are extremely prevalent and have many applications in medicinal science.^3–5^ Also, these versatile heterocyclic compounds can be fused to produce famous and significant complex heterocycles such as quinazoline, imidazoquinazoline, imidazopyridine, and pyrazoloimidazols that have synergic effects in some cases. These compounds are mostly inhibitors of α-glucosidase, antitubercular, antileishmania, anticonvulsant, antimalarial, antifungal, anticancer, antidiabetic, antihypertensive, anti-HIV, antidepressant agents and several articles^6–10^ have reported various methods to synthesize these beneficial compounds in medical science. One of the best ways with a minimum number of synthetic steps is multi-component reactions (MCRs).^11,12^ In MCRs, three or more reactants are added in a single vessel and produce the target product, usually with acceptable efficiency.^13^ These reactions are facile, fast, convergent, economic, and environmentally friendly with minimal waste generation, high yields, and no use of extra chemical solvents.^14,15^ One of the important classes of MCRs used for the one-pot synthesis of the fused imidazoles is Groebke–Blackburn–Bienaymé reaction (GBBR).^16,17^ In this reaction, imidazopyridines are synthesized using raw materials including an aldehyde, an isocyanide, and a heterocyclic compound containing an amidine fragment in the presence of various catalysts.^18^ Recently, several groups have improved this reaction and obtained various types of N-fused heterocyclic compounds by changing starting materials using different catalysts.^19–21^

In recent decades, cerium compounds which are the most abundant lanthanide reagents, have received considerable attention as efficient catalysts and oxidation reagents in organic synthesis.^22^ One of the most stable and available types of cerium is cerium(iii) chloride heptahydrate (CeCl_3_·7H_2_O).^23^ The ion Ce(iii) in CeCl_3_ is a hard cation and has a strong interaction with the compounds containing oxygen and nitrogen atoms according to the “hard and soft acids and bases” terminology of Pearson.^24^ Therefore, it can be used as an effective Lewis acid catalyst in chemical reactions. Also, low toxicity, low cost, availability, reusability, and recoverability from water are other advantages of CeCl_3_·7H_2_O.^23^

The novel SARS-CoV-2 disease (COVID-19) has become the 5^th^ documented pandemic since 1918 and has led to infection of more than 530 million people and death of over 6 million people, as yet (based on World Health Organization data). Thus, scientists immediately tried to find a suitable vaccine or drug. They obtained good successes in the field of vaccine preparation and introduced several vaccines. However, they could not make an effective drug up to now in spite of using a number of drugs.^25–27^ For instance, remdesivir has been recognized as a promising antiviral medication against a wide array of RNA viruses such as hepatitis C, ebola and COVID-19 administering *via* injection into a vein for emergency use to treat COVID-19 in many countries. As another example, chloroquine is an anti-malarial and autoimmune disease drug which has been reported as a broadspectrum antiviral drug recently. This drug can block virus infection by increasing endosomal pH required for virus. Molnupiravir and nirmatrelvir are also two new oral anti-viral agents that recently developed and used for adult patients with only mild-to-moderate COVID-19. The use of molnupiravir has some limitations, including high risk for people with >60 years of age, active cancer, chronic kidney diseases, diabetes and so on.^27^

**Fig. 1:**
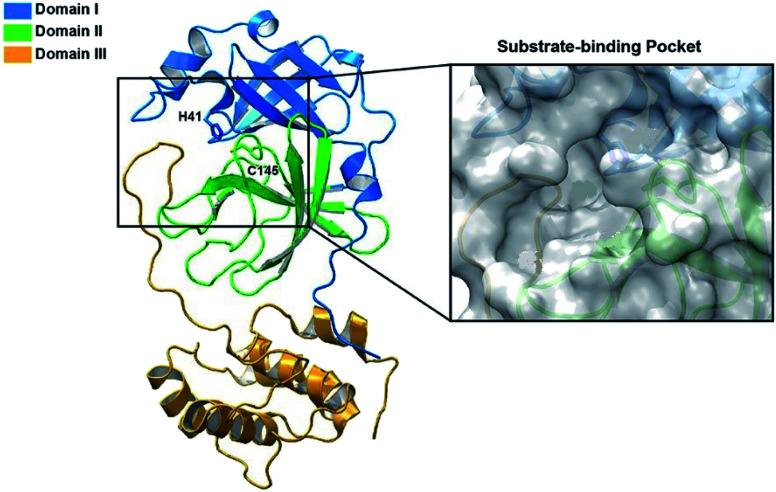
The structure of SARS-CoV-2 Mpro. Ribbon representation of SARS-CoV-2 Mpro (PDB ID: 6LU7) (the substrate-binding pocket with the transparent surface is shown).

It was found that the main protease (Mpro) of coronavirus known chymotrypsin-like protease (3CLpro) plays a vital role in the life cycle of the SARS-CoV-2 virus.^28^ Therefore, inhibition of this cycle using effective drugs could provide a hopeful therapeutic principle for developing strategic and specific treatment against this virus. The main protease protein structure is a homodimer with two chains, A and B based on the 6LU7 from PDB.^29,30^ Each protein consists of three domains (Fig. 1). Domain I (residues 8–101) and domain II (residues 102–184) forms an antiparallel beta-barrel structure and domain III (residues 201–303) is comprised of five alpha-helices arranged with an antiparallel globular structure and connected to domain II through a long loop region (residues 185–200). Mpro protein of SARS-CoV-2 has a catalytic dyad including Cys–His that the substrate-binding site is located in the cleft between domain I and II like Mpro of SARS virus,^31,32^ which can become an attractive target for designing anti-CoV drugs.^33^ Nevertheless, exploring new potent compounds that can be bound to this binding pocket and prevent the activity of the virus is very important. Considering the acceptable influence of quinazolines and imidazoles on the COVID-19 virus according to *in silico* studies,^34–37^ we synthesized some novel derivatives of pyrazolo[5′,1′:2,3]imidazo[1,5-*c*]quinazolin-6(5*H*)-ones using isocyanides, 3-aminopyrazoles, and isatin derivatives as the starting materials in the presence of CeCl_3_·7H_2_O catalyst and examined the affinity of each compound against the main protease (MPro) target of COVID-19 through *in silico* evaluations.

## 2. Results and discussion

### 2.1 Synthesis and characterization of pyrazolo[5′,1′:2,3]imidazo[1,5-*c*]quinazolin-6(5*H*)-ones

**Scheme 1:**
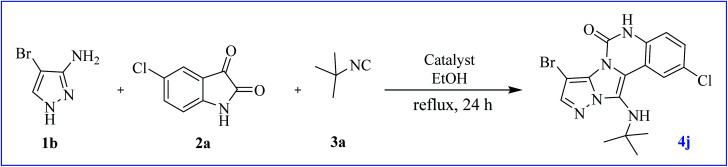
Synthesis of compound 4j using 5-chloroisatin, 3-amino-4-bromo-1*H*-pyrazole, and *tert*-butyl isocyanide.

At first, the reaction of 5-chloroisatin (1 mmol), 3-amino-4-bromo-1*H*-pyrazole (1 mmol), and *tert*-butyl isocyanide (1 mmol) was carried out in ethanol (EtOH) at the reflux condition without any catalyst for 24 h (Scheme 1). As expected, no reaction occurred (Table 1, entry 1). Therefore, as shown in Table 1, the reaction was performed using different amounts of various Lewis and Brønsted acid catalysts. As can be seen, among them, CeCl_3_·7H_2_O was the best and 8-bromo-12-(*tert*-butylamino)-2-chloropyrazolo[5′,1′:2, 3]imidazo[1,5-*c*]quinazolin-6(5*H*)-one (compound 4j) was synthesized with high yields in the presence of the CeCl_3_·7H_2_O (30 mol%) catalyst.

**Table 1:** Effects of various catalysts and different amounts of CeCl_3_·7H_2_O on the yield of compound 4j^a^

Entry	Catalyst (mol%)	Yield (%)
1	—	0
2	AcOH (30)	44
3	ZnCl_2_ (30)	32
4	Sc (OTf)_3_ (30)	51
5	NH_2_SO_3_H (30)	60
6	*p*-TsOH (30)	70
7	MgCl_2_ (30)	37
8	MeSO_3_H (30)	31
9	H_2_SO_4_ (30)	32
10	CeCl_3_·7H_2_O (20)	65
11	CeCl_3_·7H_2_O (30)	75
12	CeCl_3_·7H_2_O (40)	75
13	CeCl_3_·7H_2_O (50)	75

a Reaction conditions: 5-chloroisatin (1 mmol), 3-amino-4-bromo-1*H*-pyrazole (1 mmol), *tert*-butyl isocyanide (1 mmol) and ethanol (3 mL), reaction time: 24 h.

Then, the solvent, temperature and time were optimized. The results of these experiments are reported in Table 2. First, the reaction was performed in various solvents with different polarities in the presence and absence of CeCl_3_·7H_2_O catalyst. The results showed that no product was formed without catalyst in all the solvents while the reaction was carried out in the presence of CeCl_3_·7H_2_O catalyst (Table 2, entries 1–9). Also, under solvent-free condition, no reaction happened. Thus, it was found that the solvent has a significant effect on this reaction and ethanol was selected for the next experiments. In the next step, the reaction temperature was optimized in ethanol in the presence of the optimized amount of catalyst. As shown in Table 2 (entry 16), reflux temperature was the optimum temperature. To obtain the best time, the reaction was carried out under optimized reaction conditions for different times (1, 1.5, 2, 10, and 24 h, Table 2, entries 17–20). According to the obtained results, 2 h was selected as the optimum reaction time.

**Table 2:** The results of optimization of reaction conditions for compound 4j synthesis^a^

Entry	Solvent	Temperature (°C)	Time (h)	Yield^b^ (%)
1	Ethanol	Reflux	24	75
2	Water	Reflux	24	50
3	Methanol	Reflux	24	61
4	DMSO	100	24	52
5	DMF	100	24	52
6	Dioxane	100	24	28
7	THF	Reflux	24	40
8	Acetonitrile	Reflux	24	38
9	Ethyl acetate	Reflux	24	30
10	Chloroform	Reflux	24	0
11	Toluene	100	24	0
12	Solvent-free	100	24	0
13	Ethanol	RT	24	0
14	Ethanol	40	24	0
15	Ethanol	60	24	34
16	Ethanol	Reflux	24	75
17	Ethanol	Reflux	10	75
18	Ethanol	Reflux	2	75
19	Ethanol	Reflux	1.5	60
20	Ethanol	Reflux	1	41

a Reaction conditions: 5-chloroisatin (1 mmol), 3-amino-4-bromo-1*H*-pyrazole (1 mmol), *tert*-butyl isocyanide (1 mmol), solvent (3 mL).

b Isolated yield in the presence of CeCl_3_·7H_2_O catalyst (30 mol%).

**Scheme 2:**
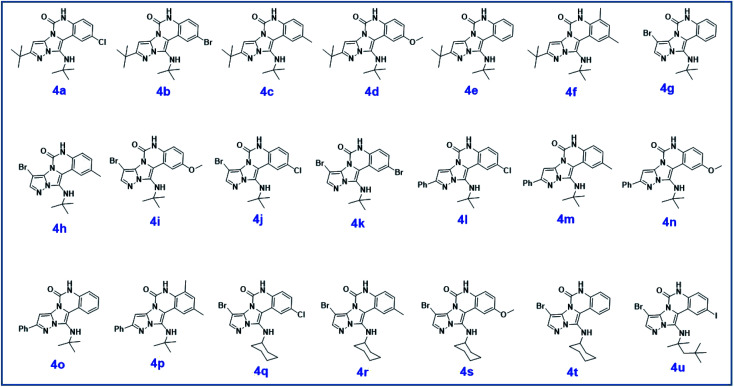
The structure of pyrazolo[5′,1′:2,3]imidazo[1,5-*c*]quinazolin-6(5*H*)-one derivatives synthesized under optimized reaction conditions in this work.

To prove the molecular structure of the product (compound 4j), ^1^H NMR, ^13^C NMR, IR, and MASS analysis were used. For example, the ^1^H NMR spectrum showed a sharp singlet peak at 1.26 ppm corresponding to the *tert*-butyl protons and another singlet at 4.95 ppm related to the proton of the NH group. The aromatic protons including H_A_, H_B,_ and H_C_ appeared at 7.16 (doublet, ^3^*J* = 8.7 Hz), 7.41 (doublet of doublet, ^3^*J*_AB_ = 8.7 Hz and ^4^*J*_BC_ = 2.4 Hz), and 8.54 ppm (doublet, ^4^*J* = 2.4 Hz), respectively. This spectrum also exhibited a sharp singlet at 7.79 ppm for the pyrazolic hydrogen and a wide singlet at 11.33 ppm for the proton of the amide NH group. Moreover, the ^1^H decoupled ^13^C NMR spectrum showed 14 signals, confirming the suggested structure. The IR spectrum of this compound exhibited the absorption bands at 3322 and 3211 cm^−1^ attributed to the NH stretching vibrations and the peaks at 1708 and 1579 cm^−1^ due to the presence of carbonyl group and C=C bonds, respectively. Also, the molecular ion peak at *m*/*z* 407 (13.8%), *m* + 2/*z* 409 (17.9%), and *m* + 4/*z* 411(4.5%) in the mass spectrum revealed the presence of Cl and Br atoms, affirming the structure of this compound. After confirming the structure of compound 4j, other derivatives (4a–4u) were synthesized under optimized reaction conditions (Scheme 2) and the structure of these new compounds was determined by various spectroscopic techniques (the results were presented in ESI†).

The yields of the synthesized compounds are listed in Table 3. As shown, the products were synthesized in good yields using different derivatives of isatin, 3-aminopyrazole, and isocyanide in the presence of CeCl_3_·7H_2_O catalyst. According to the results, the product yield has not been significantly influenced by various substituents of the isatin and pyrazole as well as using different isocyanides.

**Table 3:** The yields of synthesized products (4a–4u) using different derivatives of isatin, 3-aminopyrazole and isocyanide in the presence of CeCl_3_·7H_2_O catalyst^a^

Entry	Pyrazole	Isatin	Isocyanide	Product	Yield (%)
1	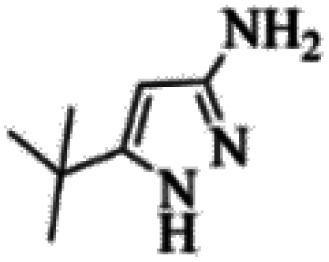	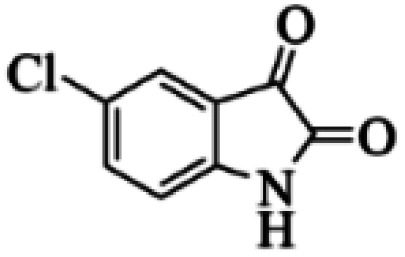	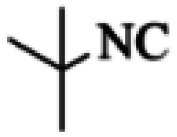	4a	78
2	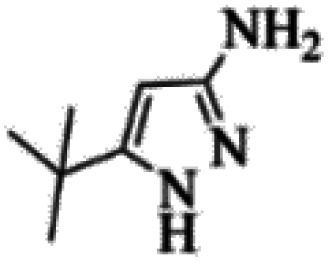	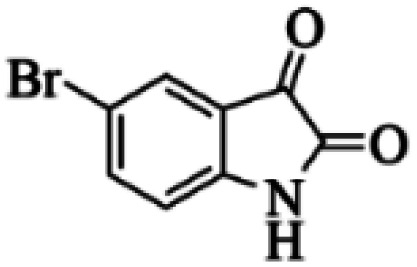	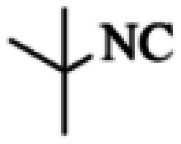	4b	76
3	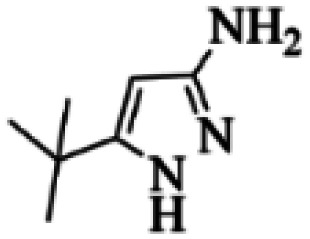	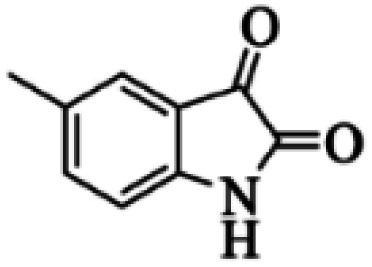	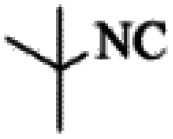	4c	80
4	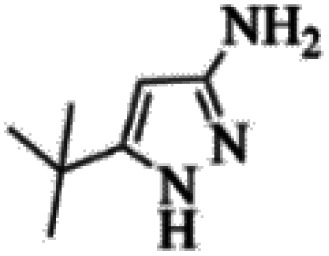	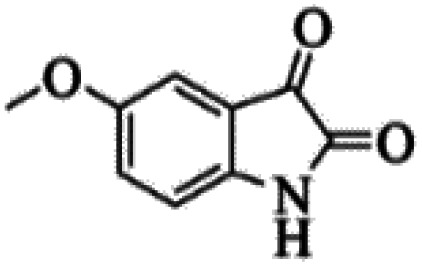	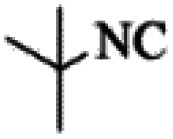	4d	77
5	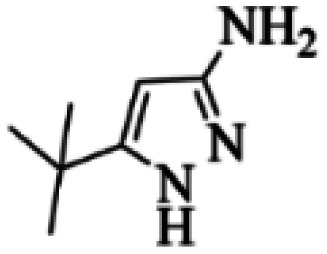	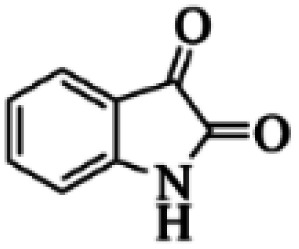	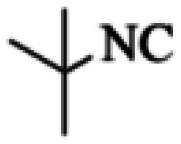	4e	75
6	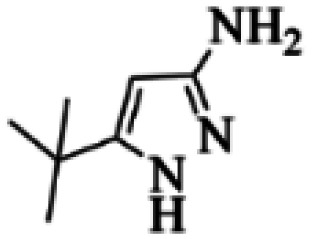	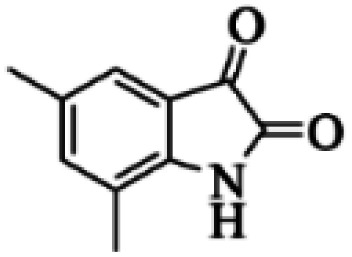	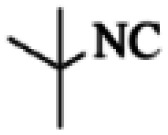	4f	82
7	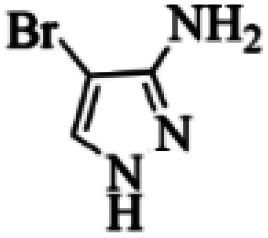	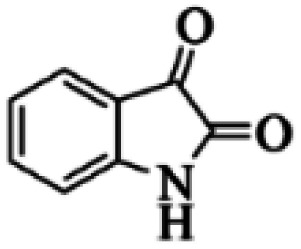	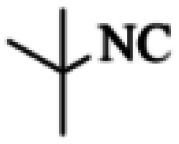	4g	74
8	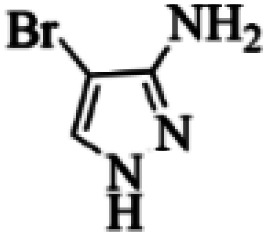	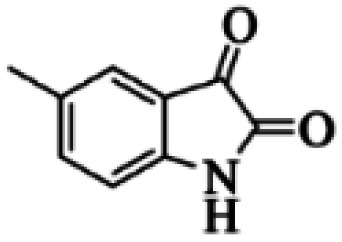	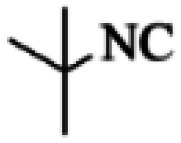	4h	73
9	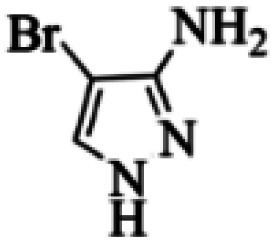	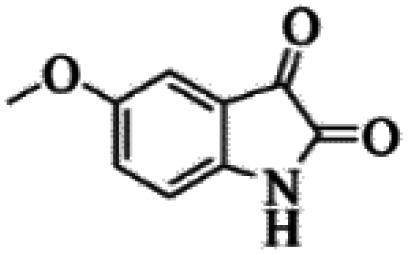	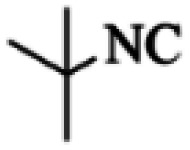	4i	74
10	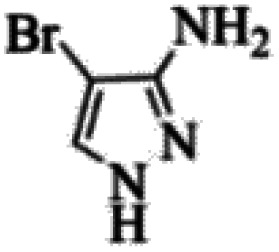	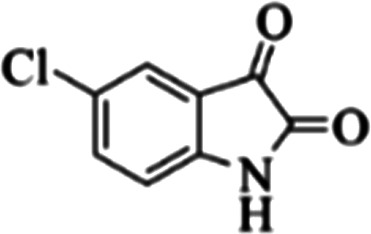	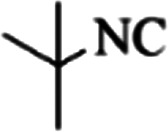	4j	77
11	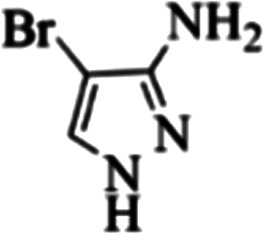	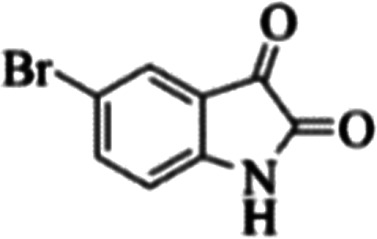	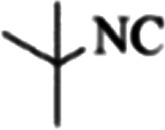	4k	79
12	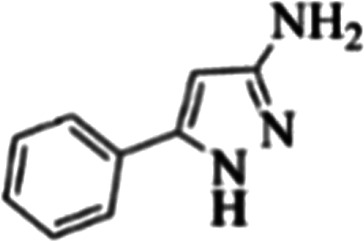	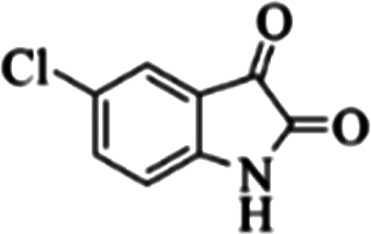	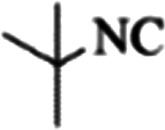	4l	81
13	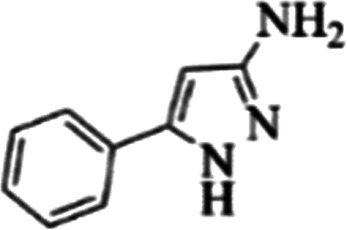	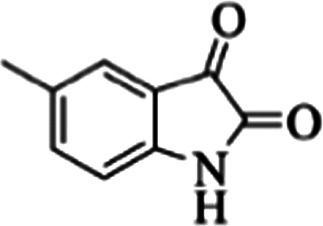	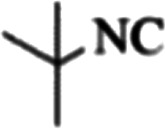	4m	81
14	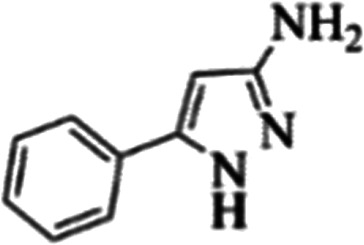	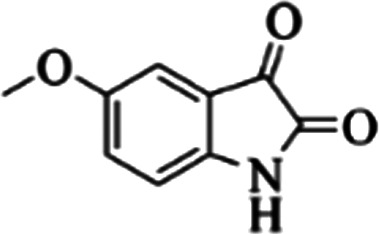	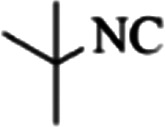	4n	82
15	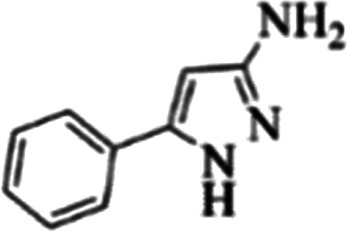	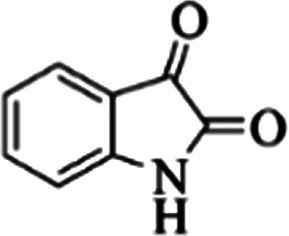	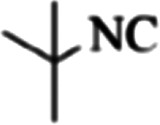	4o	80
16	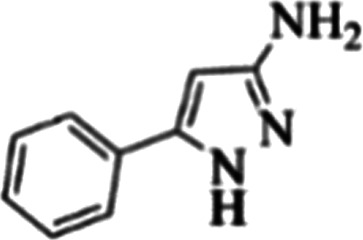	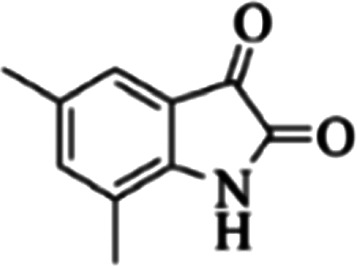	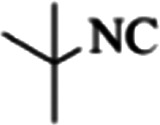	4p	81
17	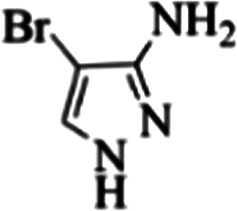	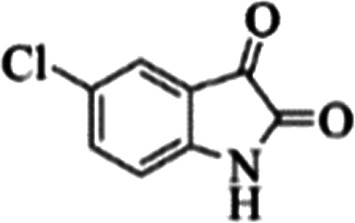	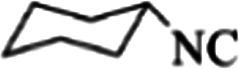	4q	75
18	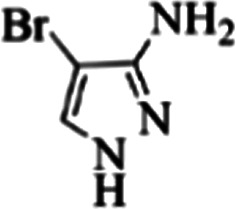	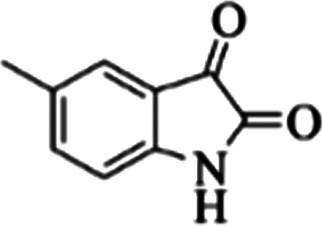	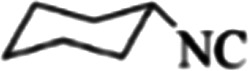	4r	79
19	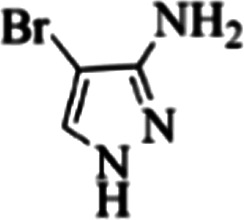	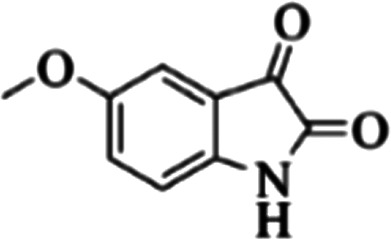	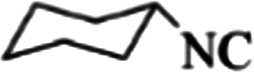	4s	75
20	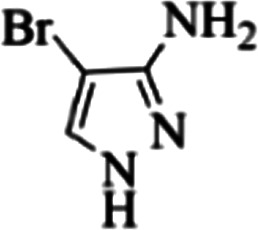	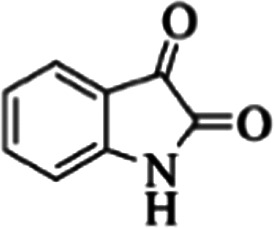	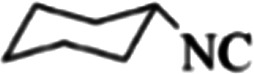	4t	74
21	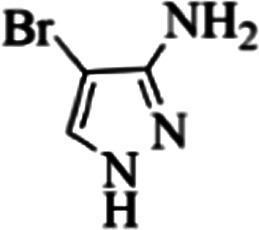	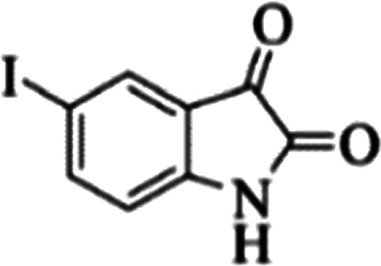	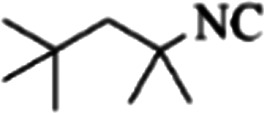	4u	63

a Isolated yield in the presence of CeCl_3_·7H_2_O catalyst (30 mol%).

**Scheme 3:**
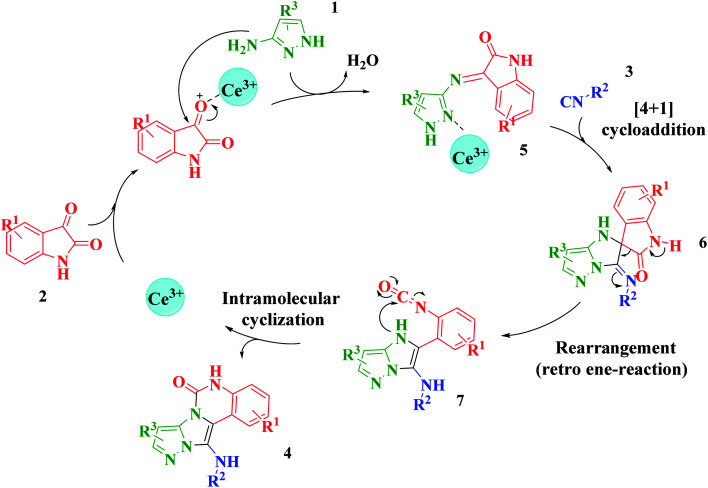
Proposed mechanism for the formation of pyrazolo[5′,1′:2,3]imidazo[1,5-*c*]quinazolin-6(5*H*)-one derivatives using acid catalysts.

Based on the structure of the synthesized compounds, a mechanism was proposed for this reaction. As illustrated in Scheme 3, the carbonyl group of isatin is activated by the acid catalyst and reacts with amino pyrazole to give imine intermediate 5. Then, [4 + 1] cycloaddition with isocyanide occurs and results in a spiro intermediate 6. In the next step, the intermediate 7 is formed by an intermolecular retro ene-reaction and ring opening of intermediate 6, followed by an intramolecular nucleophilic reaction to give the final product.

**Scheme 4:**
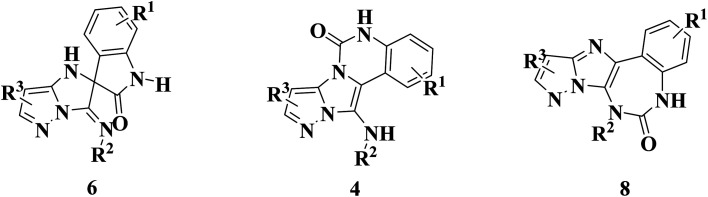
Tree possible products in this reaction.

**Fig. 2:**
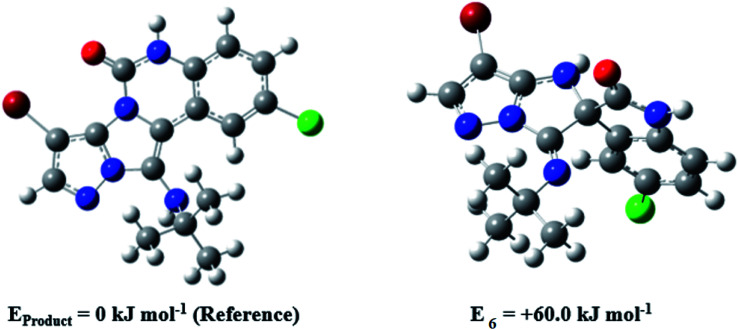
Comparison of the stability of intermediate 6 and the final product in compound 4j based on the B3LYP/6-311++G(d,p) computational study.

During this reaction, formation of three products was possible (Scheme 4). The presence of a singlet peak at 4.95 ppm in ^1^HNMR related to the proton of the NH group rejects the formation of structure 8. To ensure the rearrangement of intermediate 6 and its conversion to the final product 4j, 2D NMR techniques (HMBC and HSQC) and DFT computations were used (ESI†). In the ^1^H–^13^C HMBC NMR spectrum of compound 4j, a correlation peak (H/C: 4.95/30.57) was observed correlating the methyl carbons of the *tert*-butyl unit with NH proton, indicating the synthesis of the desired product. In addition to the spectroscopic study, DFT computations at the B3LYP/6-311++G(d,p) level of theory were carried out to verify the obtained results. Geometry optimization revealed that intermediate 6 was more unstable than the final product (Fig. 2).

### 2.2 Investigation of the pyrazolo[5′,1′:2,3]imidazo[1,5-*c*]quinazolin-6(5*H*)-ones affinity against the COVID-19 main protease through molecular docking

**Fig. 3:**
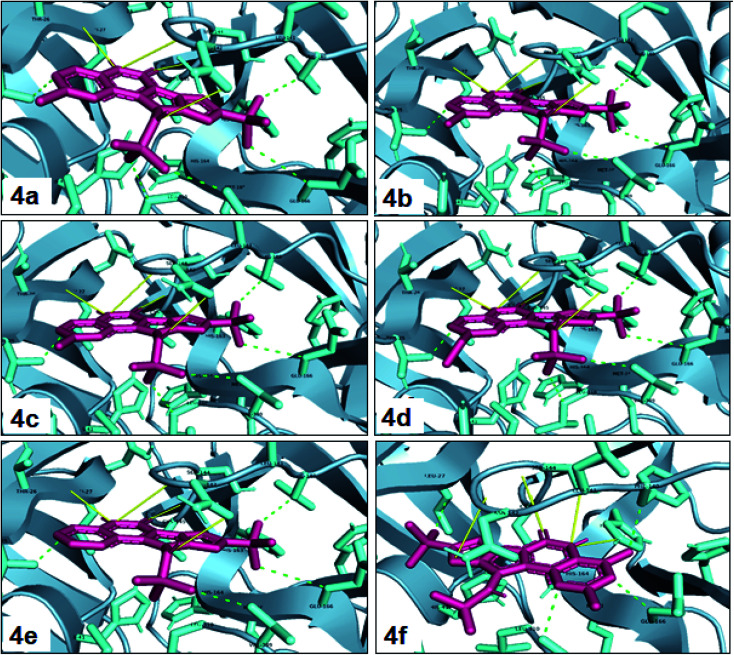
View of the Mpro binding site (Cys145–His41) for compounds 4a–4f. Image of hydrophobic interaction, hydrogen and halogen bonds for ligands (yellow and green color indication H-bond and hydrophobic interaction, respectively) prepared by pymol software.

**Fig. 4:**
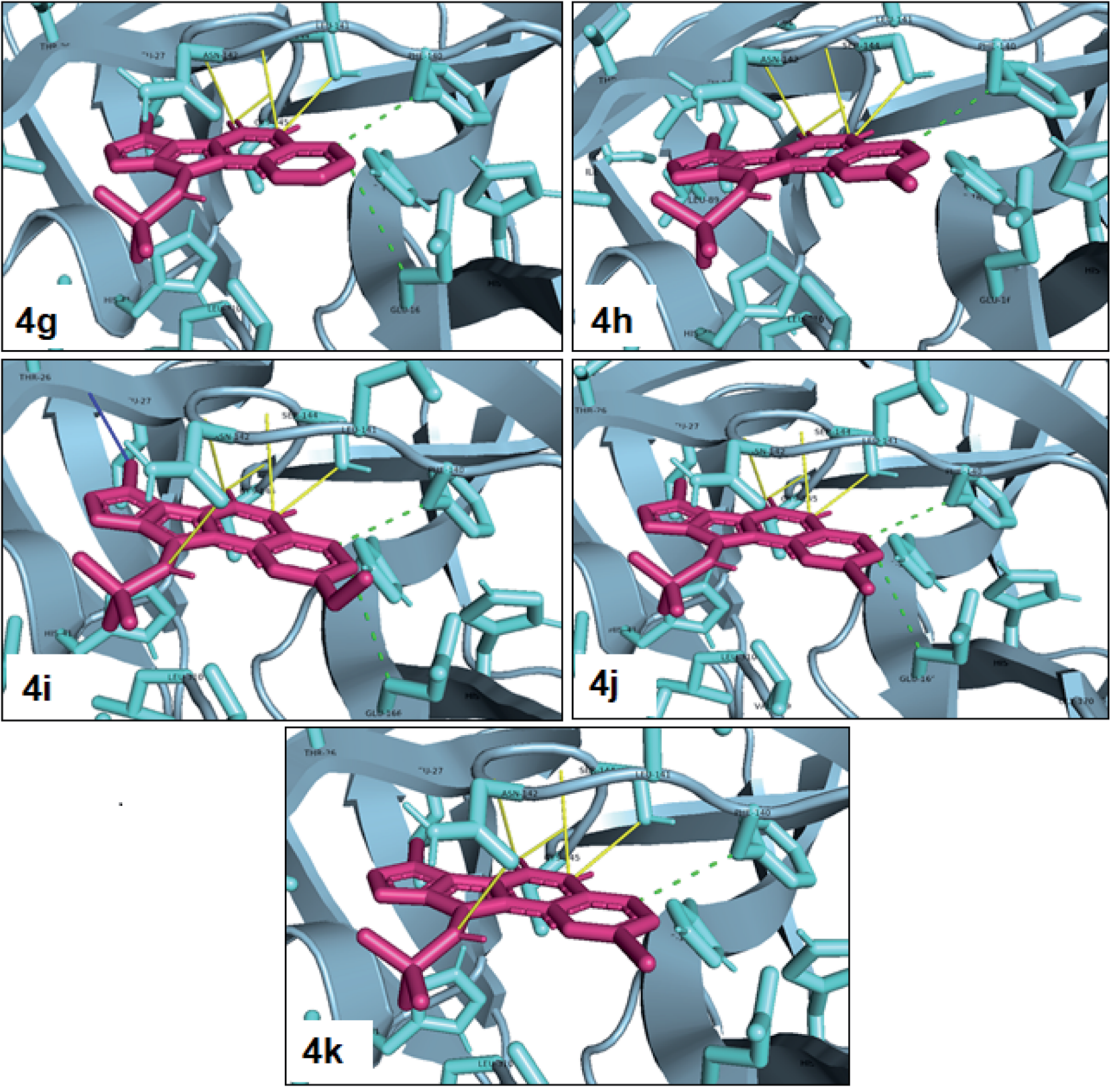
View of the Mpro binding site (Cys145–His41) for compounds 4g–4k. Image of hydrophobic interaction, hydrogen and halogen bonds for ligands (yellow, green and blue color indication H-bond, hydrophobic interaction and halogen bond, respectively) prepared by pymol software.

One of the most potent approaches and important tools for structure-based drug design is molecular docking. This method has an admirable ability to predict the binding energy of compounds to the suitable target binding site. Also, it predicts the virtual orientation of a molecule when being connected to another molecule or an active pocket and creates a stable complex with minimal free energy. Therefore, this method was used to evaluate the affinity of the synthesized compounds against the COVID-19 main protease. The results of docked model suggested that the ligands were bound between domain I and domain II of Mpro. In Fig. 3, 4, S89 and S90,† the binding style and docking poses of ligands–protein and nearby amino acids are shown. Similarly, the standard binding free energies (Δ*G*°) explain the affinity of the compounds for binding to Mpro through halogen bonds, hydrogen bonds and hydrophobic interaction presented in Tables 4–7. Also, the figures show that all compounds interacted with Mpro through hydrogen bonds and hydrophobic interactions.

**Table 4:** Molecular docking results for the interaction between Mpro and compounds 4a–4f

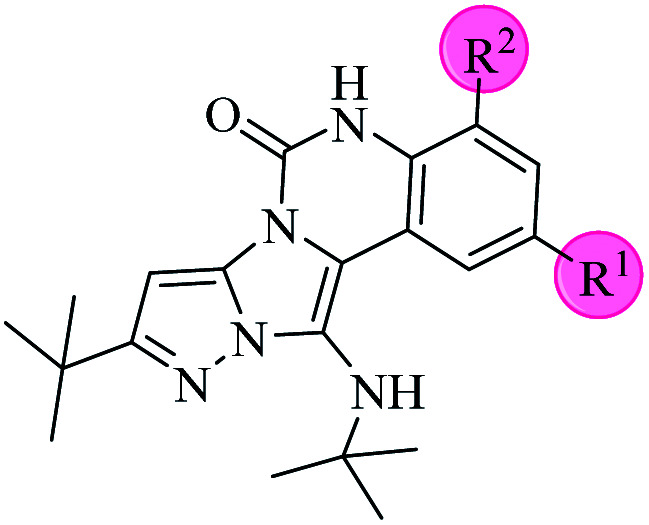						
Compound	R^1^	R^2^	Energy (kcal mol^−1^)	Hydrogen bond	Halogen bond	Hydrophobic interaction
4a	Cl	—	−8.30	THR26, ASN142, GLY143, CYS145	—	THR25, PHE140, GLU166, VAL309, LEU310
4b	Br	—	−8.44	THR26, ASN142, GLY143, CYS145	—	THR25, PHE140, GLU166, VAL309, LEU310
4c	Me	—	−8.26	THR26, ASN142, GLY143, CYS145	—	THR25, PHE140, GLU166, VAL309, LEU310
4d	OMe	—	−8.05	THR26, ASN142, GLY143, CYS145	—	THR25, PHE140, GLU166, VAL309, LEU310
4e	—	—	−8.13	THR26, ASN142, GLY143, CYS145	—	THR25, PHE140, GLU166, VAL309
4f	Me	Me	−8.22	SER144, HIS163, GLY143, CYS145	—	THR25, PHE140, GLU166, VAL309

**Table 5:** Molecular docking results for the interaction between Mpro and compounds 4g–4k

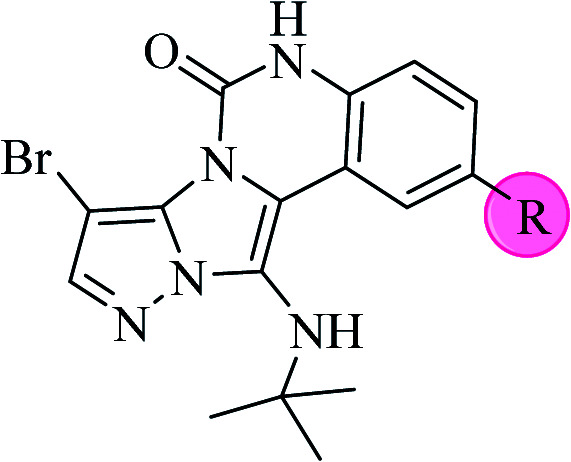					
Compound	R	Energy (kcal mol^−1^)	Hydrogen bond	Halogen bond	Hydrophobic interaction
4g	—	−7.96	GLY143, SER144, CYS145	—	PHE140, GLU166
4h	Me	−8.22	GLY143, SER144, CYS145	—	PHE140
4i	OMe	−7.90	ASN142, GLY143, SER144, CYS145	—	PHE140, GLU166
4j	Cl	−8.18	GLY143, SER144, CYS145	—	PHE140, GLU166
4k	Br	−8.28	ASN142, GLY143, SER144, CYS145	—	PHE140

**Table 6:** Molecular docking results for the interaction between Mpro and compounds 4l–4p

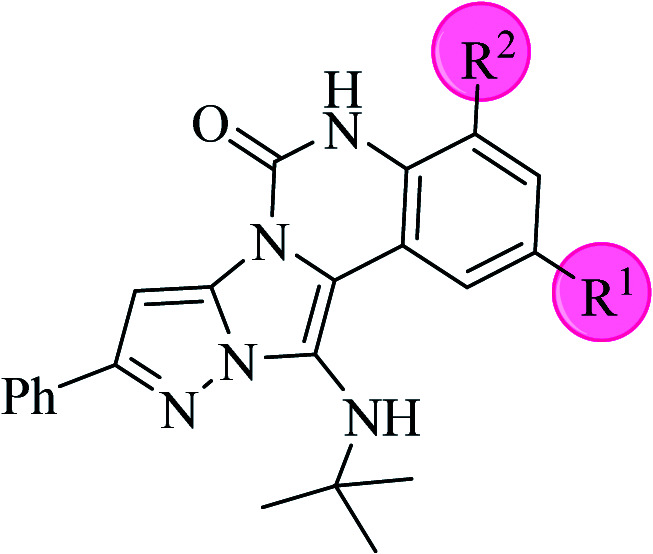						
Compound	R^1^	R^2^	Energy (kcal mol^−1^)	Hydrogen bond	Halogen bond	Hydrophobic interaction
4l	Cl	—	−8.51	SER144, ASN142, GLY143, CYS145	—	THR25, PHE140, GLU166
4m	Me	—	−8.63	SER144, GLY143, CYS145	—	THR25, PHE140, GLU166
4n	OMe	—	−8.39	SER144, ASN142, GLY143, CYS145	—	THR25, PHE140, GLU166
4o	—	—	−8.41	HIS163, SER144, GLY143, CYS145	—	THR25, THR26, GLU166, PHE140
4p	Me	Me	−7.62	ASN142, GLY143	—	LEU27, PHE140, GLU166, VAL309

**Table 7:** Molecular docking results for the interaction between Mpro and compounds 4q–4u

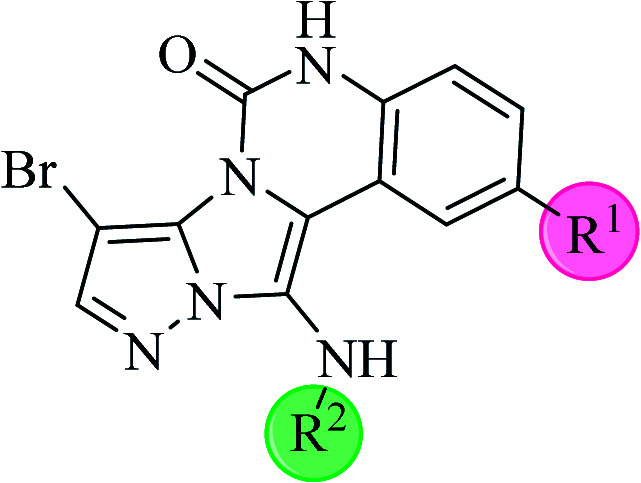						
Compound	R^1^	R^2^	Energy (kcal mol^−1^)	Hydrogen bond	Halogen bond	Hydrophobic interaction
4q	Cl	chx	−8.73	ASN142, GLY143, SER144, CYS145	THR26	PHE140, GLU166
4r	Me	chx	−8.77	HIS163, GLY143, SER144, CYS145	THR26	PHE140, GLU166
4s	OMe	chx	−8.51	ASN142, GLY143, SER144, CYS145	THR26	PHE140, GLU166
4t	—	chx	−8.54	HIS163, GLY143, SER144, CYS145	THR26	PHE140, GLU166
4u	I	1,1,3,3-Tetramethylbutyl	−8.22	ASN142, GLY143, SER144, CYS145	—	PHE140, GLU166, VAL309

In the first step of *in silico* studies, the two *t*-Bu substitutions on the pyrazole ring and amine part were constant to investigate the effect of quinazolinone substitutions on the ligand–receptor binding energy (Table 4). Here, some factors such as electronegativity and resonance effects, hydrophobic interaction as well as halogen and hydrogen bonding can be influential. The best interaction obtained from the Br and Cl substituents proved that the inductive effect of withdrawing substitutions is a significant factor. However, the difference in binding energy between Br and Cl substituents is probably due to the competition of inductive effect and resonance effect. The compound with methyl substitution was in the next position owing to the hydrophobic effect of the methyl group. Therefore, for more reliability, the compound with two Me substituents was investigated. Contrary to expectations, this compound had less binding energy, probably due to the steric effects of the extra methyl group. The comparison of the binding energy of compounds 4e and 4d shows that this value for compound 4e (unsubstituted quinazolinone) is more than 4d (with OMe substitution), indicating resonance is another key factor. Meanwhile, hydrogen bonding had almost the same effect on all compounds, as presented in Table 4. Also, As can be seen, halogens illustrated stronger interactions between the ligand and the receptor. Thus, it was decided to substitute the *t*-butyl of pyrazole ring with Br. The results listed in Table 5 indicate that the binding energy has not significantly changed, illustrating that there is no effective interaction between pyrazole halogen and receptor which is approximately in agreement with the results of Table 4.

In the next step, the phenyl substitution on the pyrazole ring was tested. The results showed that the binding energies were significantly improved which could be due to the effects of the π–π stacking interactions (Table 6).

Finally, alkyl amine was replaced with cyclohexyl. Molecular docking studies showed that these compounds (4q–4t) had almost the highest binding energies (Table 7) due to the good flexibility of cyclohexyl for incorporation in the binding pocket of the protein. To examine the effect of steric effects, 1,1,3,3-tetramethylbutyl isocyanide substrate was used to synthesize compound 4u. Also, the quinazolinonic substitution was substituted by iodine with the highest radius and the lowest resonance effect among halogens. The amount of Δ*G* showed that the effect of spatial disturbance has overcome the electron induction but still this compound had a great affinity in the binding pocket.

In addition to the synthesized molecules, the binding energy of chloroquine, hydroxychloroquine, molnupiravir and nirmatrelvir drugs were calculated by this method (entries 1–3, column 3, Table 8). Also, reported binding energies were added for chloroquine, hydroxychloroquine and remdesivir drugs. The results illustrated that all the synthesized compounds had excellent binding energies compared to these drugs. Therefore, these compounds will probably have a greater inhibitory effect on Mpro activity.

**Table 8:** Molecular docking results for products and some drugs with Mpro

Entry	Products and drugs	Energy^a^ (kcal mol^−1^)	Energy^b^ (kcal mol^−1^)
1	4a–4u	−7.62 to −8.77	—
2	Chloroquine	−7.09	−5.75,^38^ −5.1,^39^ −5.9 (ref. 40)
3	Hydroxychloroquine	−7.01	−6.7,^39^ −5.21,^38^ −6.6 (ref. 40)
4	Remdesivir	—	−7.22,^41^ −6.5 (ref. 39)
5	Molnupiravir	−7.53	—
6	Nirmatrelvir	−8.91	—

a Calculated in this work.

b From other literature data.

## 3. Conclusion

In this work, new derivatives of pyrazolo[5′,1′:2,3]imidazo[1,5-*c*]quinazolin-6(5H)-one were synthesized using GBB three-component reaction in the presence of CeCl_3_·7H_2_O catalyst. The synthesis method was fast and economical and the new compounds were easily obtained in good yields by facile workup, indicating their high potential for use in various fields, especially in pharmaceutical sciences. The results of *in silico* evaluations indicated very good affinity of investigated compounds against MPro target of COVID-19 with excellent binding energies. The change of substitutions in different positions of the synthesized structures caused a change in the binding energy. The results showed that cyclohexyl substitution in the alkyl amine position causes stronger ligand interaction with the receptor and steric effects, resonance effects, electronegativity, hydrophobic interaction, halogen and hydrogen bonding, and so on can affect the binding energy.

## 4. Experimental

### 4.1 Chemicals and characterization techniques

All chemicals were purchased from Alfa-Aesar and/or Acros companies and used without any further purification. IR spectra were recorded on a JASCO FT/IR-6300 spectrometer with KBr discs. Melting points were determined by an Electrothermal 9100 apparatus. The ^1^H NMR and ^13^C NMR spectra were recorded on a Bruker-AVANCE 400 MHz NMR instrument using DMSO-d_6_ as solvent. MASS spectra were acquired on an Agilent Technologies 5975C Series Gas Chromatograph/Mass Selective Detector (GC/MSD) system at 70 eV (United States).

### 4.2 Molecular docking study

Before consideration of the interaction of the synthesized compounds with Mpro, the geometry of twenty-one compounds were optimized by the Gaussian 09 program at the level of B3LYP/6-31G. The crystal structure of Mpro protein (PDB ID: 6LU7) was extracted from the Brookhaven Protein Data Bank (https://www.rcsb.org/pdb). Protein was prepared by removing water molecules from the pdb files and adding missed hydrogen atoms. AutoDock 4.2.5.1 molecular docking program was used with flexible-ligand docking by the executed empirical free energy function and the Lamarckian Genetic Algorithm (LGA). Then, the addition of the Gasteiger charges was done to the macromolecule input file and the Auto Grid was used to estimate grids. For performing the docking procedure of the synthesized compounds to Mpro, the center of the active site was specified and it corresponds to the coordinates: *x* = −15.383, *y* = 21.814, and *z* = 72.589 based on the catalytic dyad (Cys145–His 41). The grid size was set at 80 × 80 × 80 *XYZ* points with a grid spacing of 0.375 Å to cover the substrate-binding site in the Mpro as the center for docking. For each run, 500 docking runs with 5 000 000 energy evaluations were carried out. The results were subsequently analyzed using PYMOL software.

### 4.3 General procedure for the synthesis of pyrazolo[5′,1′:2,3]imidazo[1,5-*c*]quinazolin-6(5*H*)-ones

A solution of isatin (1 mmol), amino pyrazole (1 mmol), isocyanide (1 mmol), ethanol (3 mL), and CeCl_3_·7H_2_O (30 mol%) in a round bottom flask was heated under stirring at reflux for 2 h. The progress of the reaction was checked using TLC (petroleum ether : EtOAc (3 : 1)), and after the completion of the reaction, the obtained precipitate was filtered, washed with EtOH/H_2_O (1 : 1) solution, and dried at room temperature.

## Data availability

All datasets generated for this study were detailed in the manuscript and/or the ESI files.†

## Conflicts of interest

There are no conflicts to declare.

## Supplementary Material

RA-012-D2RA03179E-s001
