# Supplementary material for: Synthesis of pyrazolo[5′,1′:2,3]imidazo[1,5-c]quinazolin-6(5H)-ones and molecular docking study of their affinity against the COVID-19 main protease
Source: RSC Adv. 2022 Jul 8;12(30):19579–89. doi: 10.1039/d2ra03179e (PMC9264878; doi:10.1039/d2ra03179e)
Supplement: RA-012-D2RA03179E-s001 [file RA-012-D2RA03179E-s001.pdf]

Electronic Supplementary Material (ESI) for RSC Advances.
This journal is © The Royal Society of Chemistry 2022

Supplementary Information

Synthesis of pyrazolo[5',1':2,3]imidazo[1,5-c]quinazolin-6(5H)-ones and molecular docking study of their affinity against the COVID-19 main protease

Shaghayegh Saeedi, Abbas Rahmati*, Zahra Chavoshpour-Natanzi

Department of Chemistry, University of Isfahan, P.O. Box 81746-73441, Isfahan, Iran.

*Corresponding author: Tel.: 98 31 37934943, E-mail address: a.rahmati@sci.ui.ac.ir

Table of contents:

1. Supporting Material S1: Characterization Data of the compounds (**4a-4u**)……..…..S2- S10
2. Supporting Figures S1-S86: ^{1}H NMR, ^{13}C NMR, FT-IR and MASS of compounds (**4a-4u**) and 2D HMBC, HSQC NMR (**4j**)……………………………………………….S11-S75
3. Supporting Tables S1 - S4: Molecular docking results of ligand-target complex formation were summarized in the Tables…………………………………………..……..S76-S82
4. Supporting Figures S87-S90: Complexes of all ligand-target interacting systems were visually shown that prepared by pymol software…………………….………………S77-S83

1. Characterization Data of the compounds

9-(tert-butyl)-12-(tert-butylamino)-2-chloropyrazolo[5',1':2,3]imidazo[1,5-c]quinazolin-6(5H)-one (4a)

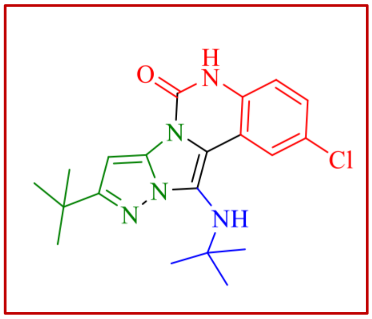

White solid; Mp: >250 °C; 1H NMR (400 MHz, DMSO-d_6) 1.27 (s, 9H), 1.33 (s, 9H), 4.89 (s, 1H), 6.15 (s, 1H), 7.11 (d, *j* = 8.6 Hz, 1H), 7.35 (dd, *j* = 8.6, *j* = 2.4 Hz 1H), 8.48 (d, *j* = 2.4 Hz, 11.27 (s, 1H);13C NMR (100 MHz, DMSO-d_6) δ 30.31, 30.59, 32.77, 55.15, 81.62, 115.09, 116.74, 117.02, 121.48, 122.01, 126.30, 127.66, 133.00, 134.40, 143.95, 164.80. IR (KBr): cm^{-1} 3392, 3319, 3059, 2961, 2770, 1707, 1581.

2-bromo-9-(tert-butyl)-12-(tert-butylamino)pyrazolo[5',1':2,3]imidazo[1,5-c]quinazolin-6(5H)-one (4b)

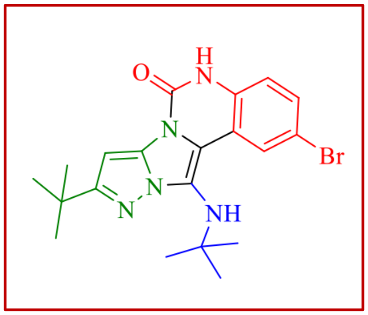

Off White solid; Mp: >250 °C; 1H NMR (400 MHz, DMSO-d_6) 1.28 (s, 9H), 1.33 (s, 9H), 4.87 (s, 1H), 6.15 (s, 1H), 7.05 (d, *j* = 8.5 Hz, 1H), 7.46 (d, *j* = 7.9 Hz, 1H), 8.63 (s, 1H), 11.26 (s, 1H);13C NMR (100 MHz, DMSO-d_6) δ 30.30, 30.59, 32.77, 55.17, 81.62, 114.08, 115.51, 116.63, 117.32, 121.43, 124.96, 130.40, 133.35, 134.38, 143.95, 164.79. IR (KBr): cm^{-1} 3396, 3329, 3059, 2960, 2766, 1707, 1581.

9-(tert-butyl)-12-(tert-butylamino)-2-methylpyrazolo[5',1':2,3]imidazo[1,5-c]quinazolin-6(5H)-one (4c)

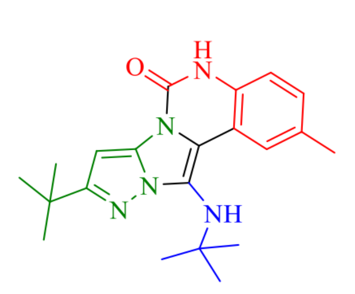

White solid; Mp: >250 °C; 1H NMR (400 MHz, DMSO-d_6) 1.28 (s, 9H), 1.34 (s, 9H), 2.33 (s, 3H), 4.71 (s, 1H), 6.13 (s, 1H), 7.02 (d, *j* = 8.2 Hz, 1H), 7.13 (d, *j* = 8.2 1H), 8.31 (s, 1H), 11.08 (s, 1H); 13C NMR (100 MHz, DMSO-d_6) δ 20.64, 30.34, 30.64, 32.73, 55.10, 81.52, 113.25, 115.22, 117.85, 120.62, 123.02, 128.83, 131.39, 131.79, 134.23, 144.20, 164.30. IR (KBr): cm^{-1} 3388, 3332, 3060, 2960, 2774, 1707, 1579.

9-(tert-butyl)-12-(tert-butylamino)-2-methoxypyrazolo[5',1':2,3]imidazo[1,5-c]quinazolin-6(5H)-one (4d)

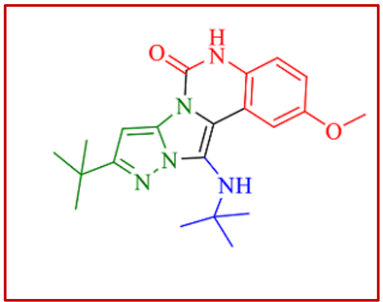

White solid; Mp: >250 °C; 1H NMR (400 MHz, DMSO-d_6) 1.43 (s, 9H), 1.47 (s, 9H), 3.93 (s, 3H), 4.92 (s, 1H), 6.26 (s, 1H), 7.07 (d, *j* = 8.8, *j* = 2.8 Hz, 1H), 7.19 (d, *j* = 8.8 Hz, 1H), 8.17 (d, *j* = 2.8 Hz, 11.15 (s, 1H); 13C NMR (100 MHz, DMSO-d_6) δ 30.41, 30.63, 32.75, 54.81, 5530, 81.51, 106.28, 114.05, 115.54, 116.52, 117.83, 120.76, 127.79, 134.31, 144.03, 154.51, 164.41. IR (KBr): cm^{-1} 3396, 3331, 3061, 2959, 2919, 2867, 1701, 1587.

9-(tert-butyl)-12-(tert-butylamino)pyrazolo[5',1':2,3]imidazo[1,5-c]quinazolin-6(5H)-one (4e)

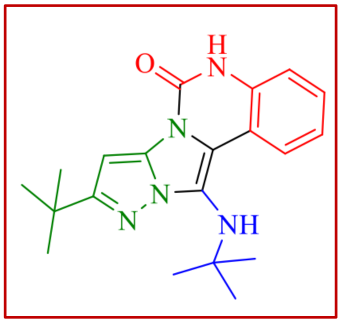

White solid; Mp: >250 °C; 1H NMR (400 MHz, DMSO-d_6) 1.28 (s, 9H), 1.34 (s, 9H), 4.72 (s, 1H), 6.15 (s, 1H), 7.12 (d, *j* = 8 Hz, 1H), 7.17 (ddd, appeared as a dt, *j* = 7.4, *j* = 1 Hz, 1H), 7.30 (ddd, appeared as a dt, *j* = 7.5, *j* = 0.9 Hz, 1H), 8.48 (dd, *j* = 7.8, *j* = 1 Hz, 1H), 11.16 (s, 1H); 13C NMR (100 MHz, DMSO-d_6) δ 30.31, 30.63, 32.73, 55.06, 81.56, 113.42, 115.33, 117.85, 120.79, 122.52, 123.02, 128.09, 134.05, 134.23, 144.21, 164.34. IR (KBr): cm^{-1} 3401, 3304, 3084, 2961, 2867, 2789, 1718, 1583.

9-(tert-butyl)-12-(tert-butylamino)-2,4-dimethylpyrazolo[5',1':2,3]imidazo[1,5-c]quinazolin-6(5H)-one (4f)

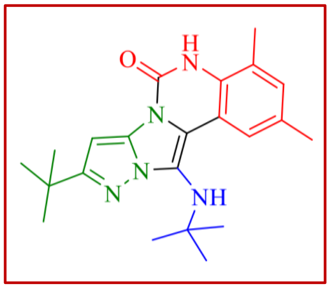

Off White solid; Mp: >250 °C; 1H NMR (400 MHz, DMSO-d_6) 1.27 (s, 9H), 1.34 (s, 9H), 2.29 (s, 3H), 2.32 (s, 3H), 4.68 (s, 1H), 6.15 (s, 1H), 6.98 (s, 1H), 8.25 (s, 1H), 10.20 (s, 1H); 13C NMR (100 MHz, DMSO-d_6) δ 17.65, 20.47, 30.33, 30.65, 32.74, 55.08, 81.69, 113.37, 117.89, 120.52, 120.93, 123.73, 130.04, 130.54, 131.05, 134.00, 144.52, 164.25. IR (KBr): cm^{-1} 3406, 3307, 3215, 2959, 2924, 2864, 1712, 1577.

8-bromo-12-(tert-butylamino)pyrazolo[5',1':2,3]imidazo[1,5-c]quinazolin-6(5H)-one (4g)

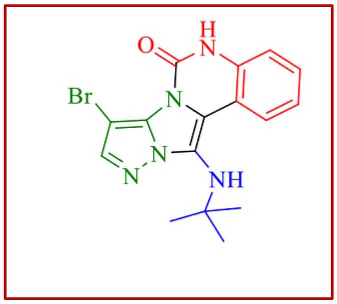

Yellow solid; Mp: 230-232 °C;1H NMR (400 MHz, DMSO-d_6) 1.25(s, 9H), 4.80 (s, 1H), 7.14 – 7.23 (m, 2H), 7.35 (ddd, appeared as a dt, *j* = 7.7, *j* = 1.3 Hz, 1H), 7.76 (s, 1H), 8.52 (dd, *j* = 7.5, *j* = 0.9 Hz, 1H), 11.22 (s, 1H); 13C NMR (100 MHz, DMSO-d_6) δ 30.30, 55.31, 70.38, 112.66, 115.32, 120.13, 120.79, 122.53, 123.35, 128.78, 131.74, 134.25, 143.29, 143.78. IR (KBr): cm^{-1} 3405, 3277, 3081, 2959, 1718,1586.

8-bromo-12-(tert-butylamino)-2-methylpyrazolo[5',1':2,3]imidazo[1,5-c]quinazolin-6(5H)-one (4h)

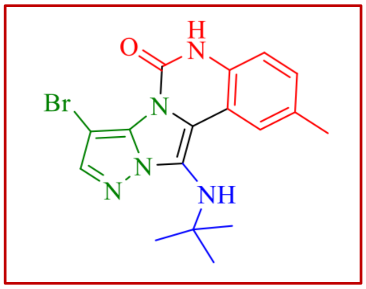

Off White solid; Mp: 225 decomposed; 1H NMR (400 MHz, DMSO-d_6) 1.26 (s, 9H), 2.34 (s, 3H), 4.80 (s, 1H), 7.06 (dd, *j* = 8.2 Hz, 1H), 7.18 (dd, *j* = 8.3 Hz, *j* = 1.6 Hz, 1H), 7.75 (s, 1H), 8.36 (s, 1H), 11.15 (s, 1H);13C NMR (100 MHz, DMSO-d_6) δ 20.63, 30.35, 55.31, 70.34, 112.51, 115.22, 120.63, 123.30, 129.53, 131.41, 131.73, 132.03, 143.26, 143.77. IR (KBr): cm^{-1} 3399, 3324, 3056, 2955, 2924, 1712, 1572.

8-bromo-12-(tert-butylamino)-2-methoxypyrazolo[5',1':2,3]imidazo[1,5-c]quinazolin-6(5H)-one (4i)

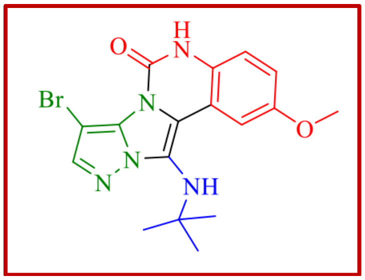

White solid; Mp: 227 decomposed; 1H NMR (400 MHz, DMSO-d_6) 1.27 (s, 9H), 3.80 (s, 3H), 4.87 (s, 3H), 6.94 – 7.31 (m, 2H), 7.76 (s, 1H), 8.09 (s, 1H), 11.10 (s, 1H);13C NMR (100 MHz, DMSO-d_6) δ 30.42, 55.02, 55.38, 70.31, 106.62, 113.30, 116.32, 116.56, 120.08, 120.78, 128.05, 128.17, 128.87, 143.39, 143.60, 154.5. IR (KBr): cm^{-1} 3399, 3331, 3208, 3097,2964, 2254, 1709, 1573.

8-bromo-12-(tert-butylamino)-2-chloropyrazolo[5',1':2,3]imidazo[1,5-c]quinazolin-6(5H)-one (4j)

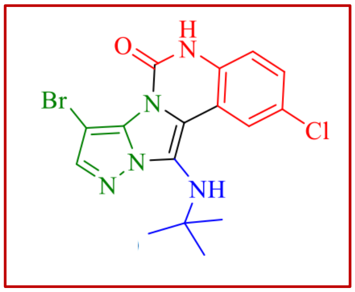

Off White solid; Mp: >250 °C; 1H NMR (400 MHz, DMSO-d_6) 1.26 (s, 9H), 4.95 (s, 1H), 7.16 (d, *j* = 8.7 Hz, 1H), 7.41 (dd, *j* = 8.7, *j* = 2.4 Hz 1H), 7.79 (s, 1H), 8.54 (d, *j* = 2.4 Hz, 1H) 11.33 (s, 1H);13C NMR (100 MHz, DMSO-d_6) δ 30.31, 55.41, 70.46, 114.34, 117.06, 119.07, 121.40,

122.36, 126.24, 128.38, 131.91, 133.28, 143.56, 143.67. IR (KBr): cm^{-1} 3407, 3322, 3090, 2962, 2760, 1709, 1579.

2,8-dibromo-12-(tert-butylamino)pyrazolo[5',1':2,3]imidazo[1,5-c]quinazolin-6(5H)-one (4k)

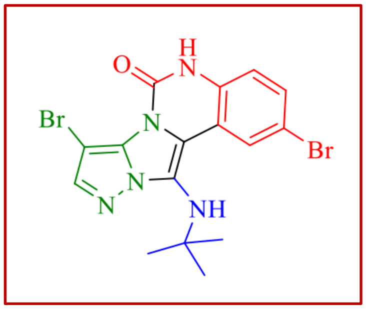

Off White solid; Mp: 243-245 °C; 1H NMR (400 MHz, DMSO-d_6) 1.26 (s, 9H), 4.95 (s, 1H), 7.11 (d, j=7.3 Hz, 1H), 7.52 (d, j=7.3 Hz, 1H), 7.79 (s, 1H), 8.69 (s, 1H), 11.33 (s, 1H); 13C NMR (100 MHz, DMSO-d_6) δ 30.31, 55.41, 70.47, 114.01, 114.78, 117.33, 118.95, 121.38, 125.32, 131.10, 131.93, 133.62, 143.55, 143.66. IR (KBr): cm^{-1} 3399, 3323, 3095, 2962, 1710, 1580.

12-(tert-butylamino)-2-chloro-9-phenylpyrazolo[5',1':2,3]imidazo[1,5-c]quinazolin-6(5H)-one (4l)

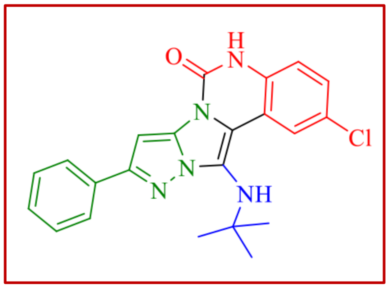

White solid; Mp: >250 °C; 1H NMR (400 MHz, DMSO-d_6) 1.34 (s, 9H), 5.02 (s, 1H), 6.80 (s, 1H),7.16 (d, *j* = 8.7 Hz, 1H), 7.33 (t, *j* = 7.4 Hz, 1H), 7.37- 7.48 (m, 3H), 7.96 (d, *j* = 7.8 Hz, 1H), 8.53 (d, *j* = 2.4 Hz, 1H), 11.41 (s, 1H);13C NMR (100 MHz, DMSO-d_6) δ 30.40, 55.32, 82.74, 114.81, 117.16, 117.92, 121.46, 122.18, 125.30, 126.41, 127.70, 128.05, 128.68, 133.17, 133.98, 135.23, 143.87, 153.31. IR (KBr): cm^{-1} 3403, 3316, 3133, 3060, 2965, 2916, 2867,1722, 1587.

12-(tert-butylamino)-2-methyl-9-phenylpyrazolo[5',1':2,3]imidazo[1,5-c]quinazolin-6(5H)-one (4m)

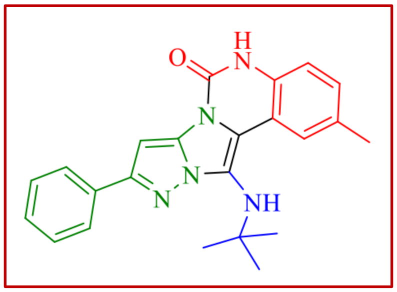

White solid; Mp: >250 °C; 1H NMR (400 MHz, DMSO-d_6) 1.46 (s, 9H), 2.48 (s, 3H), 4.97 (s, 1H), 6.90 (s, 1H),7.19 (d, *j* = 8.3 Hz, 1H), 7.30 (dd, *j* = 8.3, *j* = 1.5 Hz, 1H), 7.45 (t, *j* = 7.3 Hz, 1H), 7.56 (dd, appeared as a t, *j* = 7.6 Hz, 2H), 8.08 (dd, *j* = 7.8, *j* = 1.3 Hz, 2H), 8.49 (s, 1H), 11.34 (s, 1H);13C NMR (100 MHz, DMSO-d_6) δ 20.66, 30.43, 55.27, 82.69, 112.97, 115.35, 119.05, 120.61, 123.16, 125.22, 127.57, 128.66, 129.24, 131.54, 131.95, 134.12, 135.06, 144.10, 152.85. IR (KBr): cm^{-1} 3403, 3314, 3061, 2966, 2917, 2863, 1717, 1591.

12-(tert-butylamino)-2-methoxy-9-phenylpyrazolo[5',1':2,3]imidazo[1,5-c]quinazolin-6(5H)-one (4n)

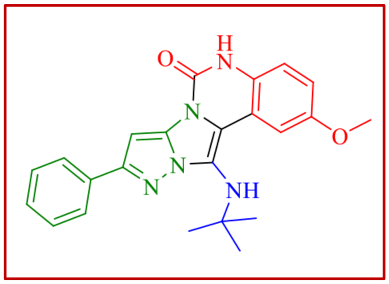

White solid; Mp: >250 °C; 1H NMR (400 MHz, DMSO-d_6) 1.48 (s, 9H), 3.95 (s, 3H), 5.06 (s, 1H), 6.90 (s, 1H),7.11 (dd, *j* = 8.8, *j* = 2.8 Hz, 1H), 7.23 (d, *j* = 8.8 Hz, 1H), 7.45 (t, *j* = 7.3 Hz, 1H), 7.56 (dd, appeared as a t, *j* = 7.6 Hz, 2H), 8.08 (dd, *j* = 8.4, *j* = 1.3 Hz, 2H), 8.21 (d, *j* = 2.8 Hz, 2H), 11.29 (s, 1H);13C NMR (100 MHz, DMSO-d_6) δ 30.51, 54.97, 55.33, 82.66, 106.35, 113.77, 115.98, 116.67, 119.01, 120.75, 125.24, 127.60, 127.96, 128.67, 134.09, 135.14, 143.92, 152.96, 154.57. IR (KBr): cm^{-1} 3392, 3315, 3064, 2962, 2924, 2870, 1712, 1587.

12-(tert-butylamino)-9-phenylpyrazolo[5',1':2,3]imidazo[1,5-c]quinazolin-6(5H)-one (4o)

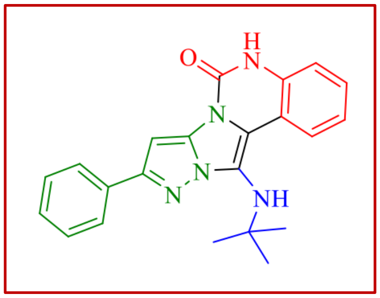

Off White solid; Mp: >250 °C; 1H NMR (400 MHz, DMSO-d_6) 1.33 (s, 9H), 4.86 (s, 1H), 6.79 (s, 1H), 7.16 (d, *j* = 8.0 Hz, 1H), 7.21 (t, *j* = 7.5 Hz, 1H), 7.29- 7.38 (m, 2H), 7.44 (t, *j* = 7.6 Hz, 2H), 7.95 (d, *j* = 7.4 Hz, 1H), 8.52 (d, *j* = 7.8 Hz, 1H) 11.29 (s, 1H); 13C NMR (100 MHz, DMSO-d_6) δ 30.40, 55.22, 82.71, 113.15, 115.46, 119.05, 120.80, 122.65, 123.18, 125.26, 127.57, 128.66, 134.12, 134.23, 135.09, 144.12, 152.92. IR (KBr): cm^{-1} 3402, 3304, 3063, 2930, 2789, 1715, 1590.

12-(tert-butylamino)-2,4-dimethyl-9-phenylpyrazolo[5',1':2,3]imidazo[1,5-c]quinazolin-6(5H)-one (4p)

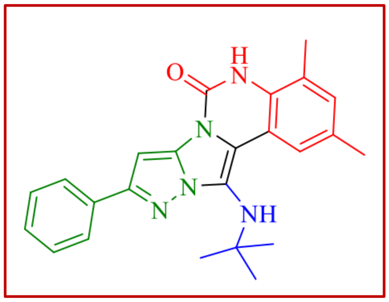

White solid; Mp: >250 °C; 1H NMR (400 MHz, DMSO-d_6) 1.32 (s, 9H), 2.31 (s, 3H), 2.34 (s, 3H), 4.81 (s, 1H), 6.80 (s, 1H), 7.03 (s, 1H), 7.31 (t, *j* = 7.3 Hz, 1H), 7.43 (t, *j* = 7.6 Hz, 2H), 7.96 (d, *j* = 7.4 Hz, 2H), 8.30 (s, 1H), 10.33 (s, 1H);13C NMR (100 MHz, DMSO-d_6) δ 17.67, 20.48, 30.43, 55.23, 82.87, 113.11, 119.08, 120.53, 121.07, 123.87, 125.24, 127.54, 128.64, 130.22, 130.92, 131.20, 134.14, 134.86, 144.42, 152.84. IR (KBr): cm^{-1} 3405, 3313, 3222, 3110, 2967, 2924, 2867, 1708, 1574.

8-bromo-2-chloro-12-(cyclohexylamino)pyrazolo[5',1':2,3]imidazo[1,5-c]quinazolin-6(5H)-one (4q)

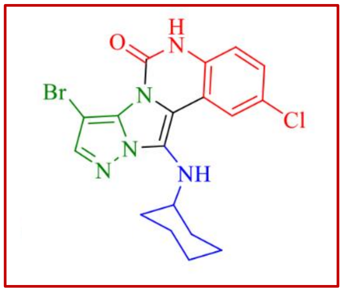

White solid; Mp: >250 °C; ^{1}H NMR (400 MHz, DMSO-d_6) 1.16 – 1.24 (m, 3H), 1.28 – 1.37 (m, 2H), 1.51 – 1.56 (m, 1H), 1.67 – 1.73 (m, 2H), 1.83 – 1.89 (m, 2H), 3.45 – 3.54 (m, 1H), 5.36 (d, *j* = 5.9 Hz, 1H), 7.14 (d, *j* = 8.6 Hz ,1H), 7.36 (dd, *j* = 8.7, 2.4 Hz, 1H), 7.80 (s, 1H), 8.28 (d, *j* = 2.3 Hz, 1H), 11.22 (s, 1H); 13C NMR (100 MHz, DMSO-d_6) δ 24.27, 25.36, 33.32, 54.28, 70.58, 114.75, 116.87, 121.64, 122.96, 126.63, 127.73, 132.84, 143.63, 144.11. IR (KBr) (ν_{max}/cm^{-1}): 3405, 3277, 3082, 2959, 1718, 1586. MS (EI, 70 eV) *m/z*: 439 (M+).

8-bromo-12-(cyclohexylamino)-2-methylpyrazolo[5',1':2,3]imidazo[1,5-c]quinazolin-6(5H)-one (4r)

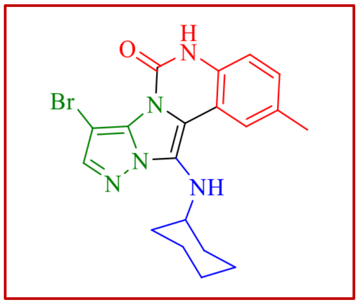

Off White solid; Mp: 243-245 °C; 1H NMR (400 MHz, DMSO-d_6) 1.17 – 1.24 (m, 3H), 1.31 – 1.39 (m, 2H), 1.51 – 1.56 (m, 1H), 1.68 – 1.74 (m, 2H), 1.83 – 1.89 (m, 2H), 2.34 (s, 3H), 3.40 – 3.48 (m, 1H), 5.11 (d, *j* = 6.0 Hz, 1H), 7.05 (d, *j* = 8.2 Hz ,1H), 7.14(dd, *j* = 8.3 Hz, *j* = 1.4 Hz, 1H), 7.76 (s, 1H), 8.11 (s, 1H), 11.04 (s, 1H); 13C NMR (100 MHz, DMSO-d_6) δ 20.66, 24.31, 25.39, 33.28, 54.53, 70.47, 112.81, 115.13, 115.91, 122.13, 122.65, 128.93, 131.64, 131.78, 143.65, 143.84. IR (KBr): cm^{-1} 3382, 3291, 2922, 2851, 1698, 1501.

8-bromo-12-(cyclohexylamino)-2-methoxypyrazolo[5',1':2,3]imidazo[1,5-c]quinazolin-6(5H)-one (4s)

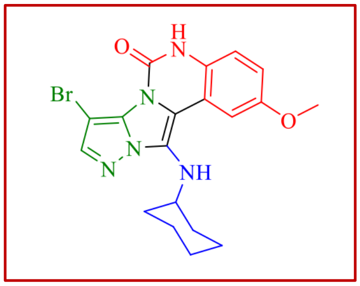

White solid; Mp: 247-248 °C; 1H NMR (400 MHz, DMSO-d_6) 1.16 – 1.24 (m, 3H)), 1.29 – 1.39 (m, 2H), 1.50 – 1.56 (m, 1H), 1.67 – 1.74 (m, 2H), 1.81 – 1.92 (m, 2H), 3.39 – 4.48 (m, 1H), 5.20 (d, *j* = 5.8 Hz, 1H), 6.96 (dd, *j* = 8.8, *j* = 2.7 Hz, 1H), 7.09 (d, *j* = 8.8 Hz, 1H), 7.77 (s, 1H), 7.85 (d, *j* = 2.7 Hz, 1H), 11.01 (s, 1H);13C NMR (100 MHz, DMSO-d_6) δ 24.18, 25.40, 33.32, 54.45, 55.31, 70.46, 106.34, 113.59, 115.37, 116.27, 116.42, 122.14, 127.68, 131.68, 143.75, 154.79. IR (KBr): cm^{-1} 3401, 3283, 3066, 2927, 1711, 1574.

8-bromo-12-(cyclohexylamino)pyrazolo[5',1':2,3]imidazo[1,5-c]quinazolin-6(5H)-one (4t)

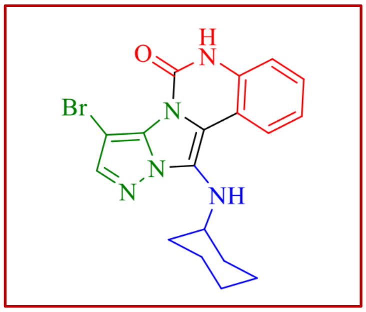

White solid; Mp: >250 °C; 1H NMR (400 MHz, DMSO-d$_6$) 1.26 – 1.34 (m, 3H), 1.39 – 1.49 (m, 2H), 1.63 – 1.69 (m, 1H), 1.78 – 1.85 (m, 2H), 1.94 – 2.01 (m, 2H), 3.52 – 3.60 (m, 1H), 5.25 (d, *j* = 5.8 Hz, 1H), 7.27 (d, *j* = 8.0 Hz, 1H), 7.32 (ddd, appeared as a dt, *j* = 8.8, *j* = 2.7 Hz, 1H), 7.44 (ddd, appeared as a dt, *j* = 7.92, *j* = 7.48, *j* = 1.24 Hz, 1H), 7.89 (s, 1H), 8.42(d, *j* = 7.04 Hz, 1H), 11.26 (s, 1H); 13C NMR (100 MHz, DMSO-d$_6$) δ 24.37, 25.39, 33.24, 54.47, 70.52, 112.97, 115.23, 116.09, 122.15, 122.66, 122.82, 128.20, 133.92, 143.68, 143.84. IR (KBr): cm^{-1} 3403, 3243, 3088, 2925, 1712, 1589.

8-bromo-2-iodo-12-((2,4,4-trimethylpentan-2-yl)amino)pyrazolo[5',1':2,3]imidazo[1,5-c]quinazolin-6(5H)-one (4u)

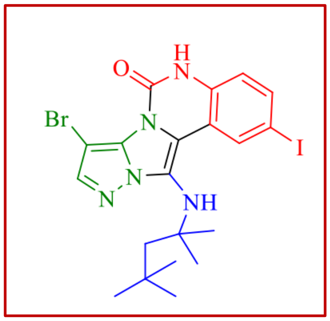

White solid; Mp: 250 decomposed; 1H NMR (400 MHz, DMSO-d$_6$) 1.22 (s, 9H), 1.37 (s, 6H), 1.91 (s, 2H), 4.85 (s, 1H), 7.08 (d, j=8.5 Hz, 1H), 7.77 (dd, *j*=8.5, *j*=1.9 Hz, 1H), 7.91 (s, 1H), 9.02 (d, *j* = 1.9 Hz, 1H), 11.42 (s, 1H); 13C NMR (100 MHz, DMSO-d$_6$) δ 29.19, 31.52, 31.70, 56.17, 59.45, 70.42, 85.61, 115.03, 117.42, 121.18, 131.23, 133.95, 136.69, 143.58. IR (KBr): cm^{-1} 3400, 3372, 3056, 2945, 2766, 1718, 1580.

^{1}H NMR, ^{13}C NMR, FT-IR and MASS of compound (4a)

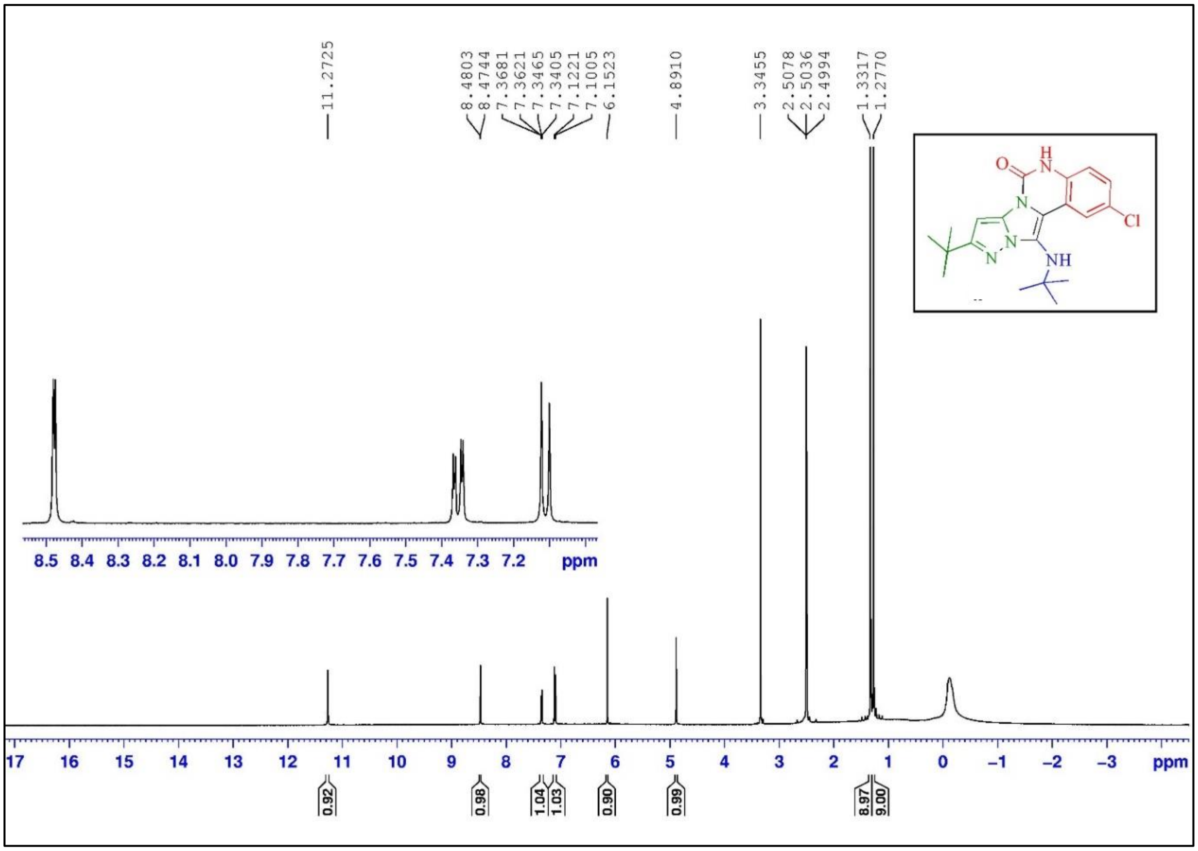

Figure S1. ^{1}H NMR spectrum of compound (4a) in DMSO.

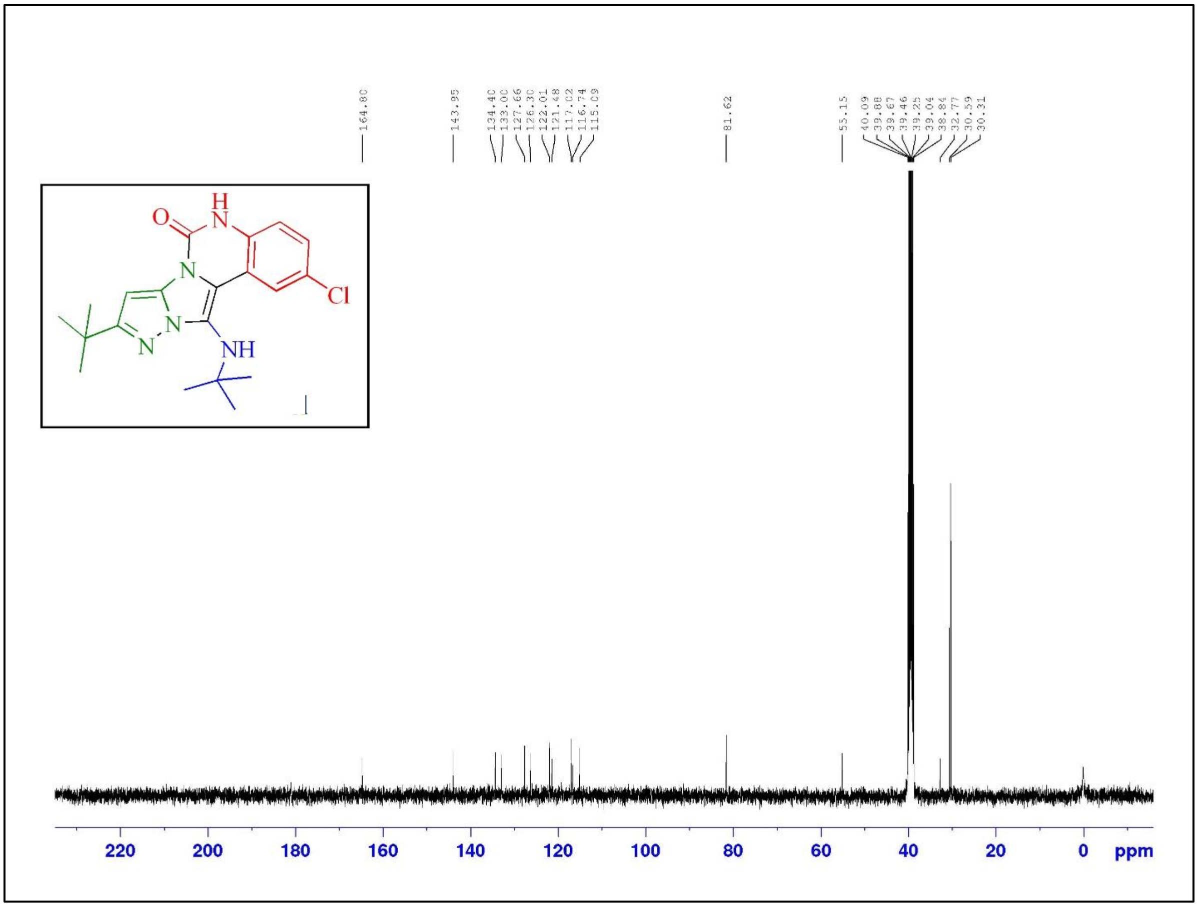

Figure S2. ${}^{13}C$ NMR spectrum of compound (4a) in DMSO.

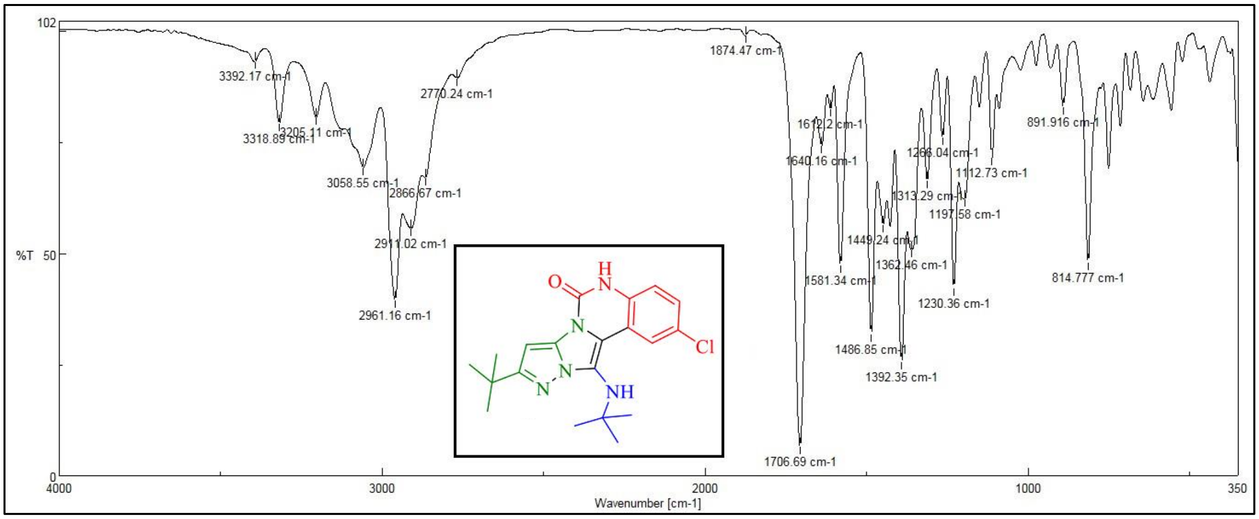

Figure S3. FT-IR spectrum of compound (4a).

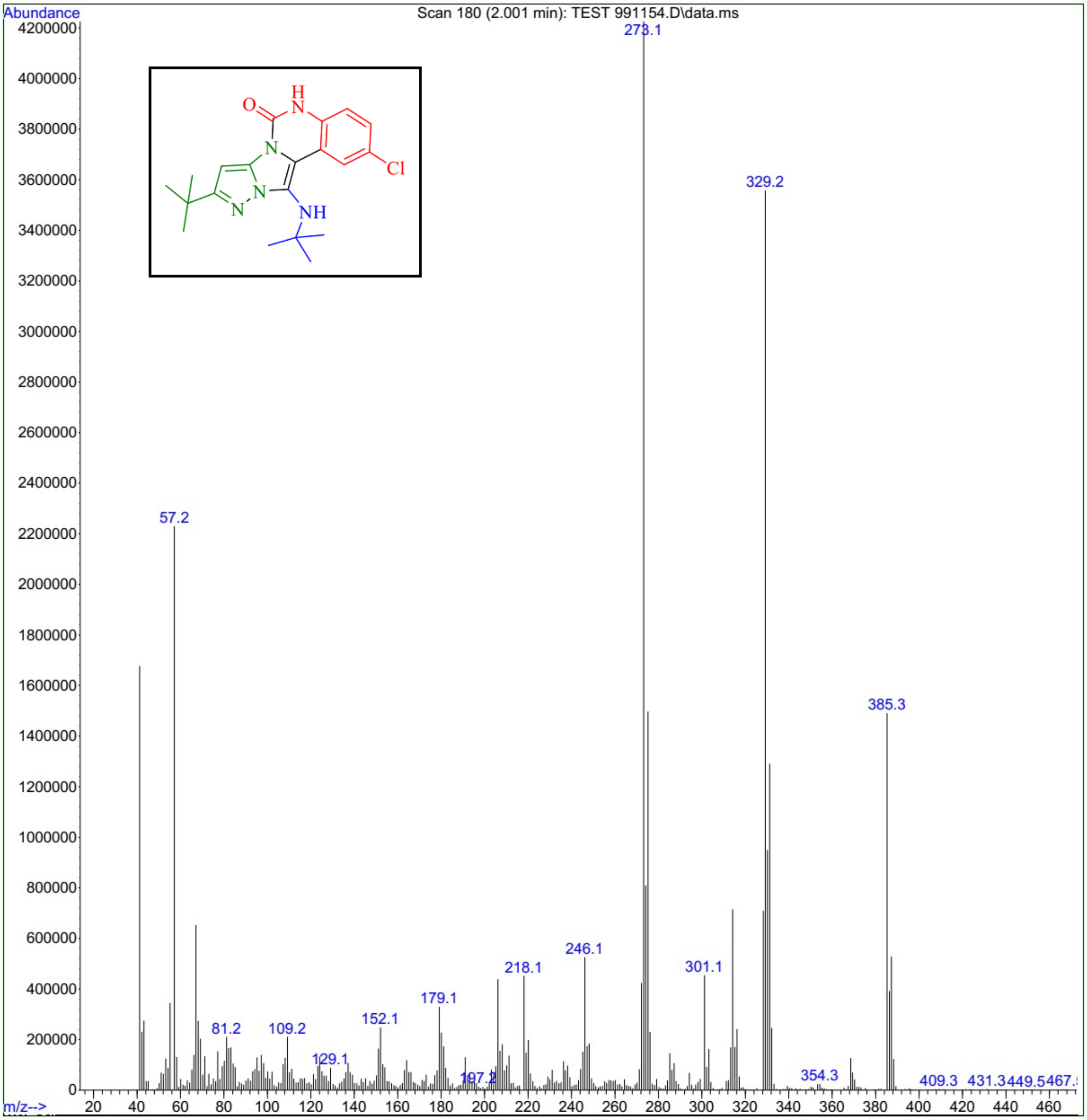

Figure S4. MASS spectrum of compound (4a).

^{1}H NMR, ^{13}C NMR, FT-IR and MASS of compound (4b)

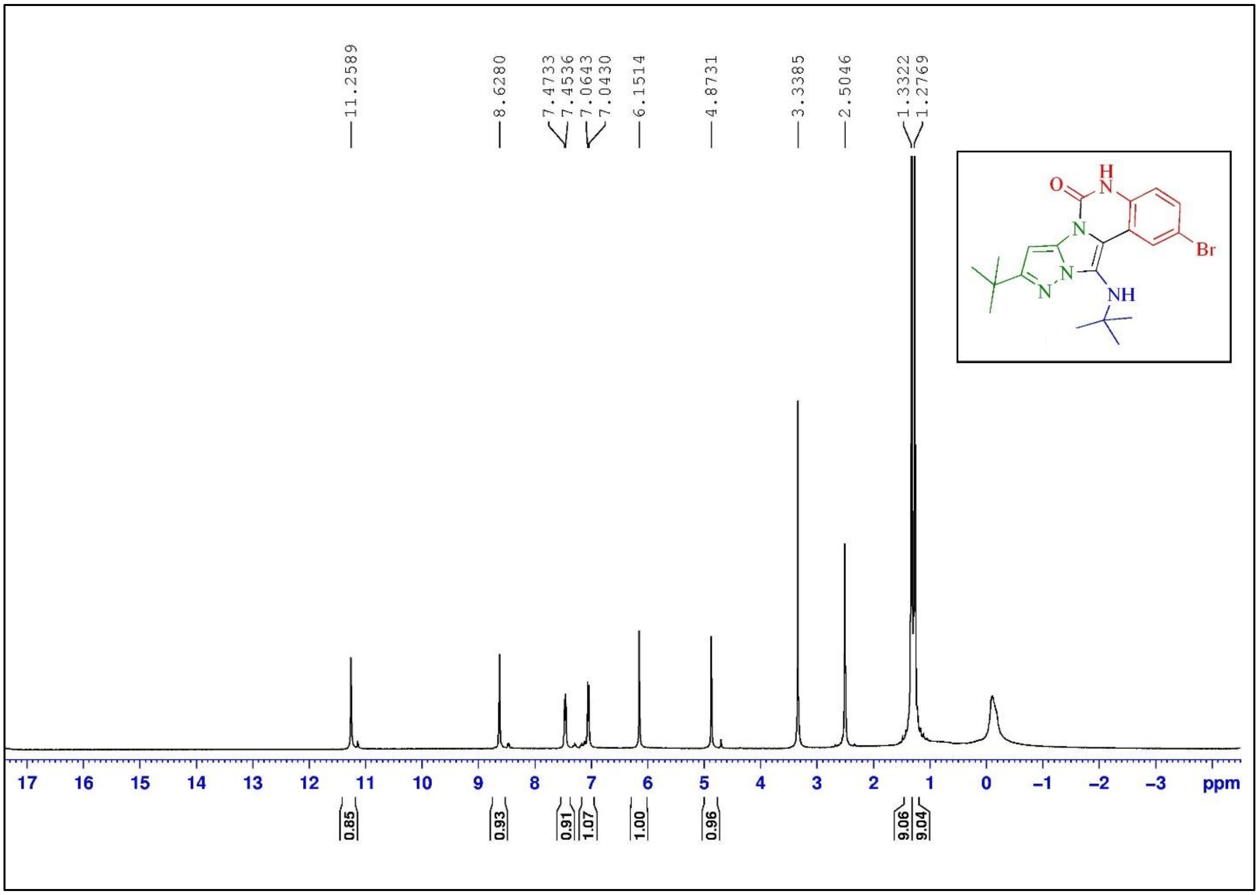

Figure S5. ^{1}H NMR spectrum of compound (4b) in DMSO.

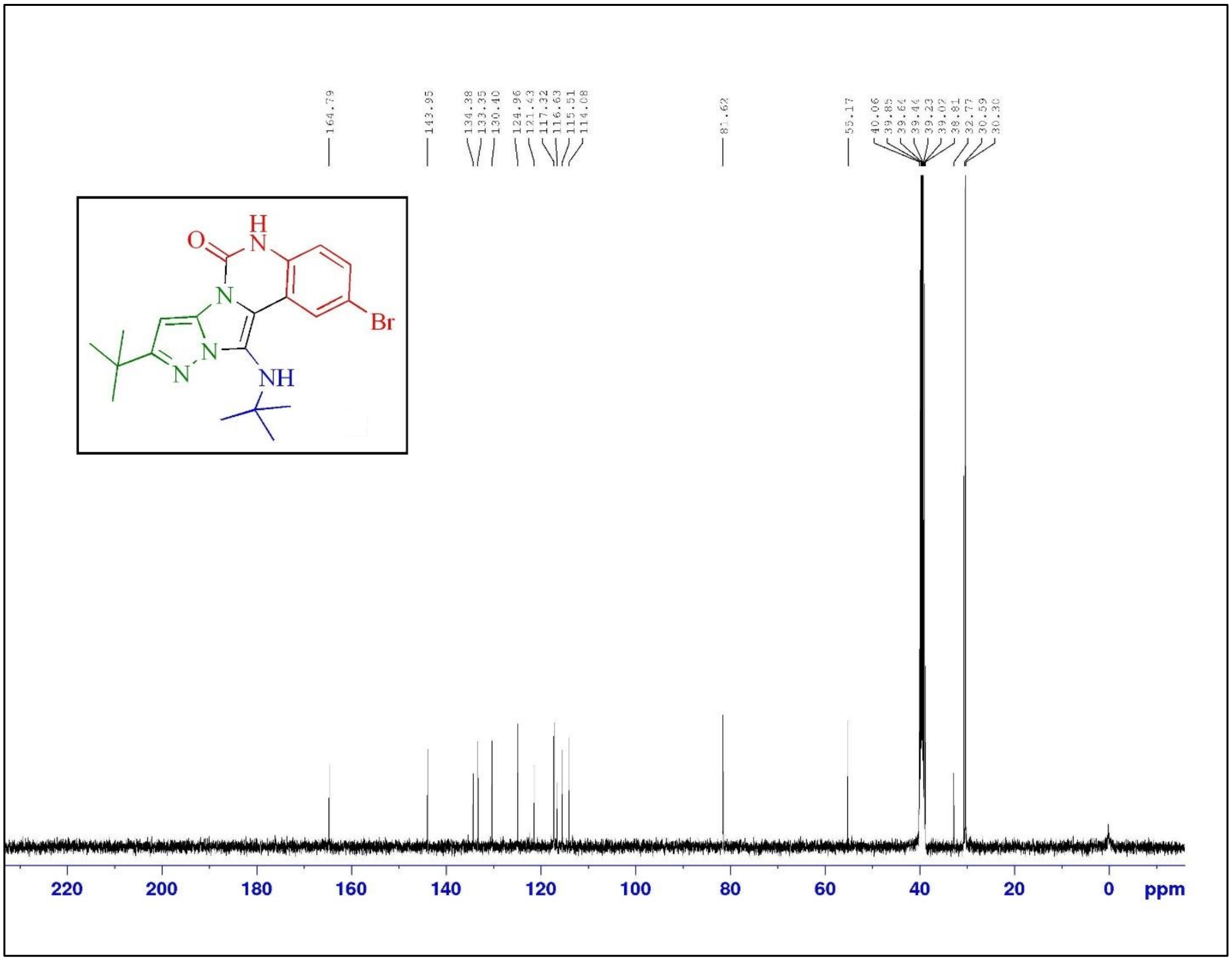

Figure S6. ^{13}C NMR spectrum of compound (4b) in DMSO.

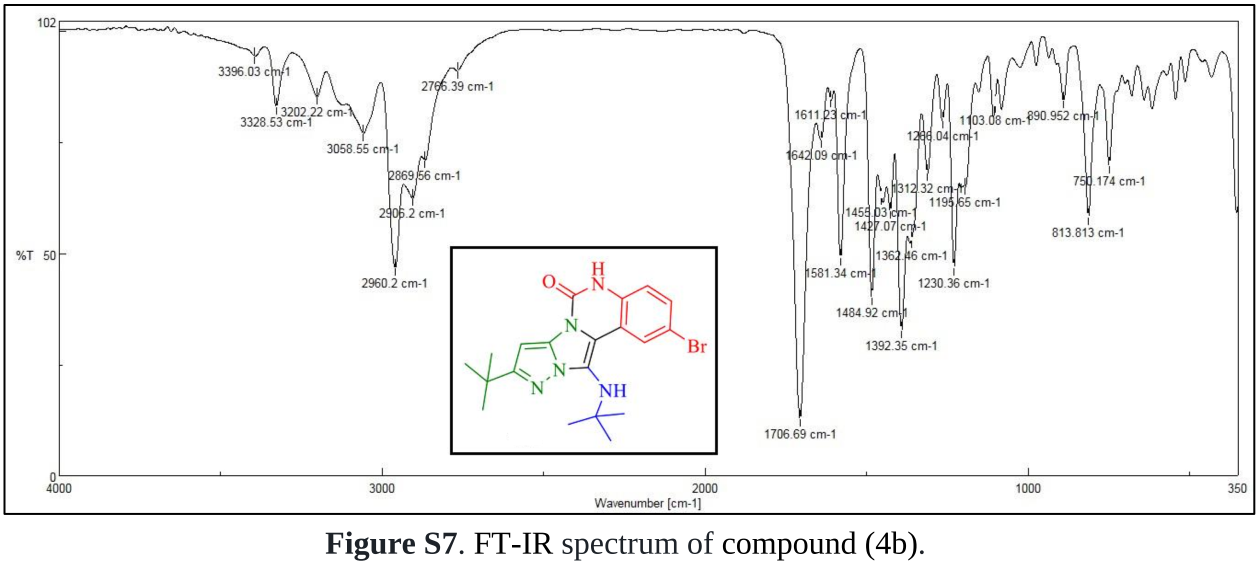

Figure S7. FT-IR spectrum of compound (4b).

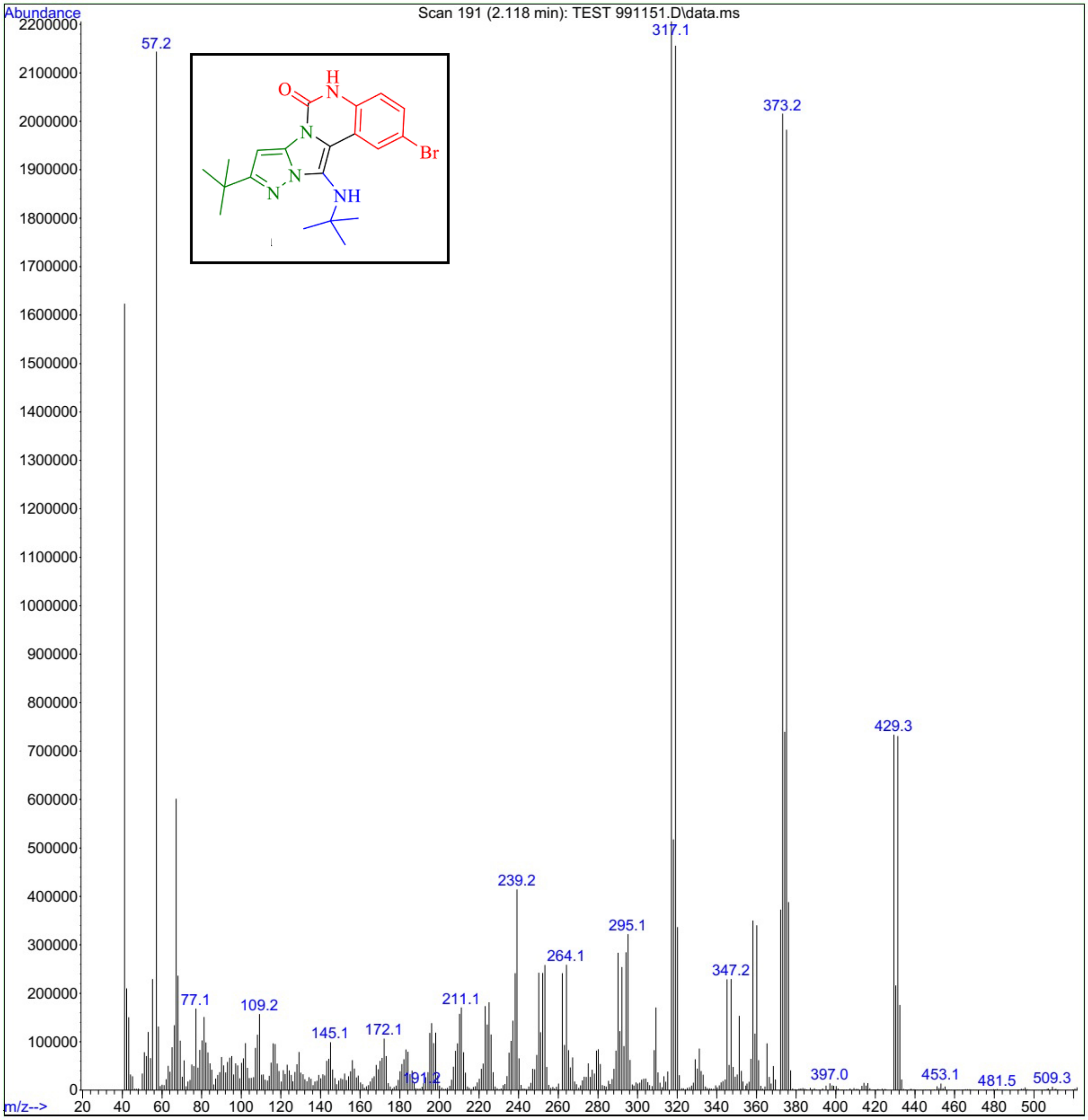

Figure S8. MASS spectrum of compound (4b).

^{1}H NMR, ^{13}C NMR, FT-IR and MASS of compound (4c)

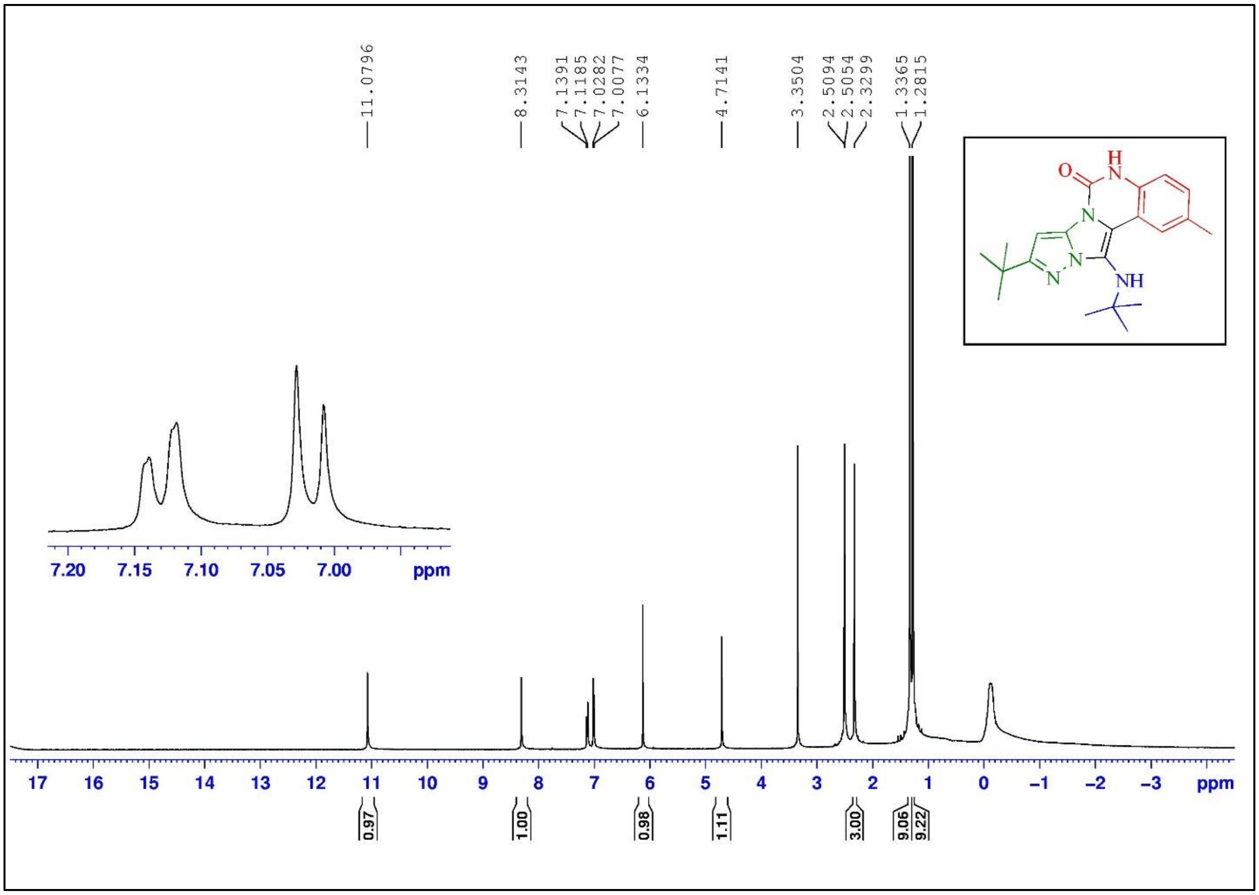

Figure S9. ^{1}H NMR spectrum of compound (4c) in DMSO.

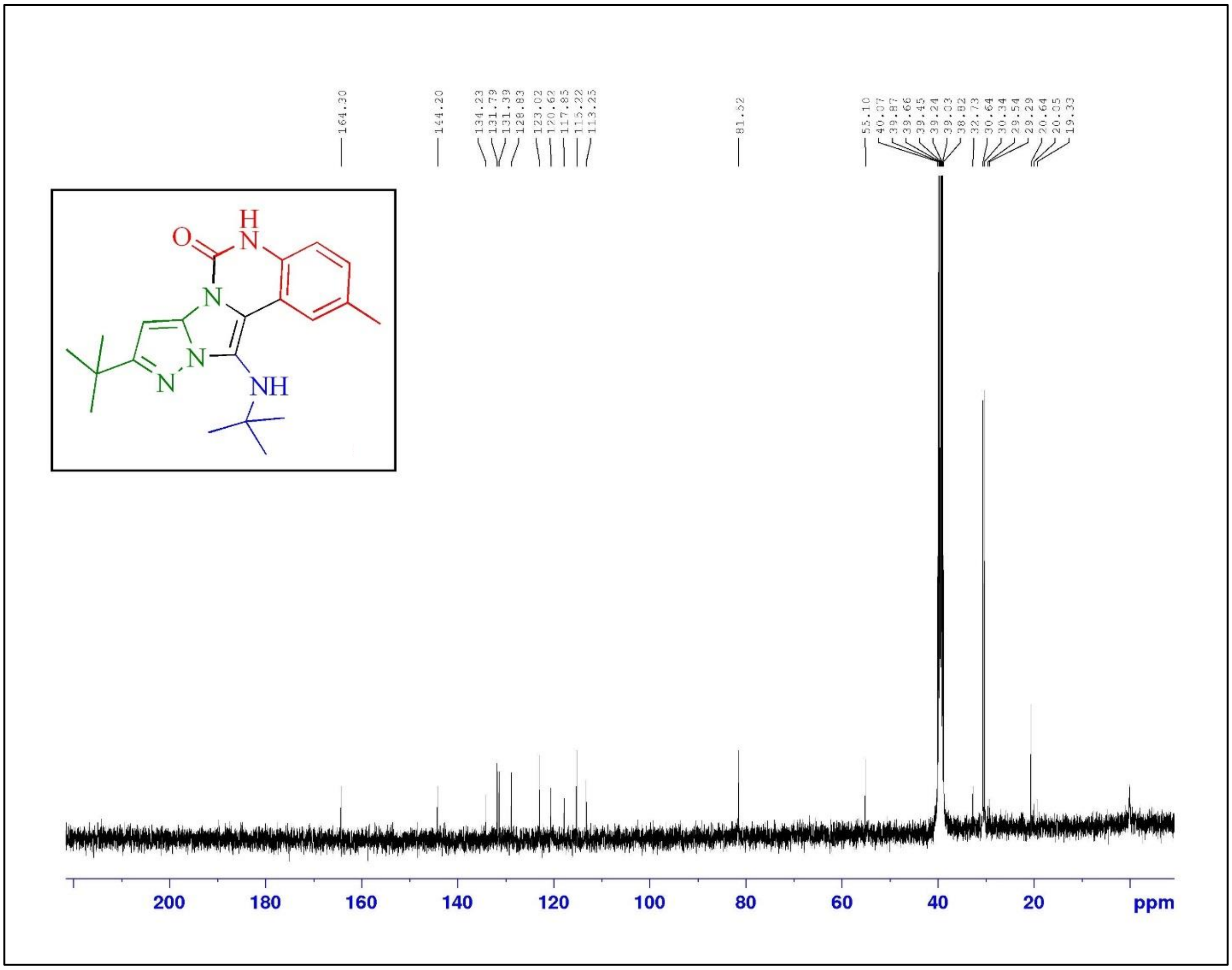

Figure S10. ^{13}C NMR spectrum of compound (4c) in DMSO.

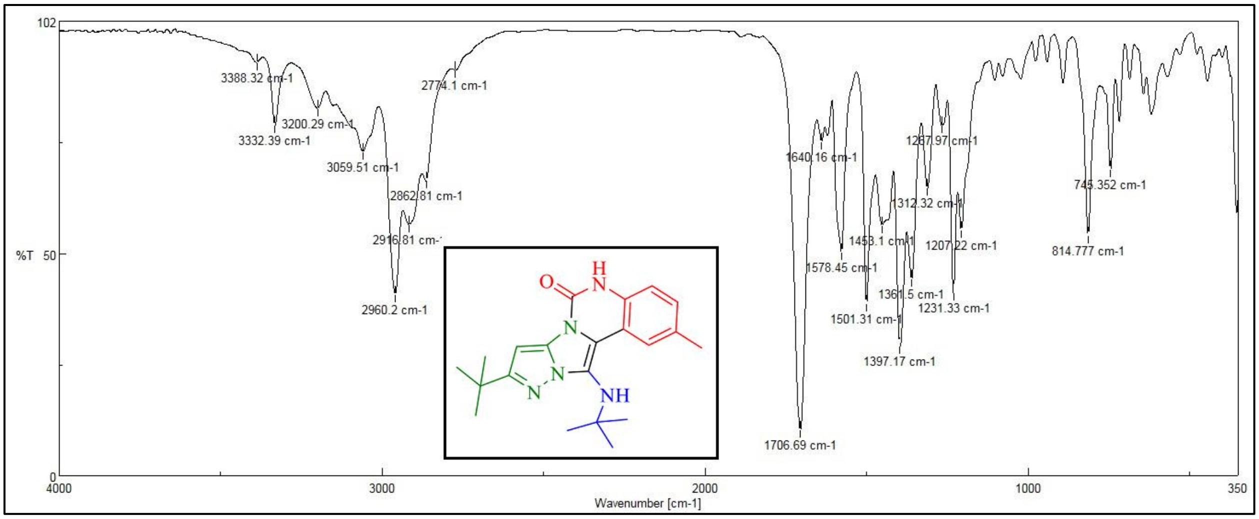

Figure S11. FT-IR spectrum of compound (4c).

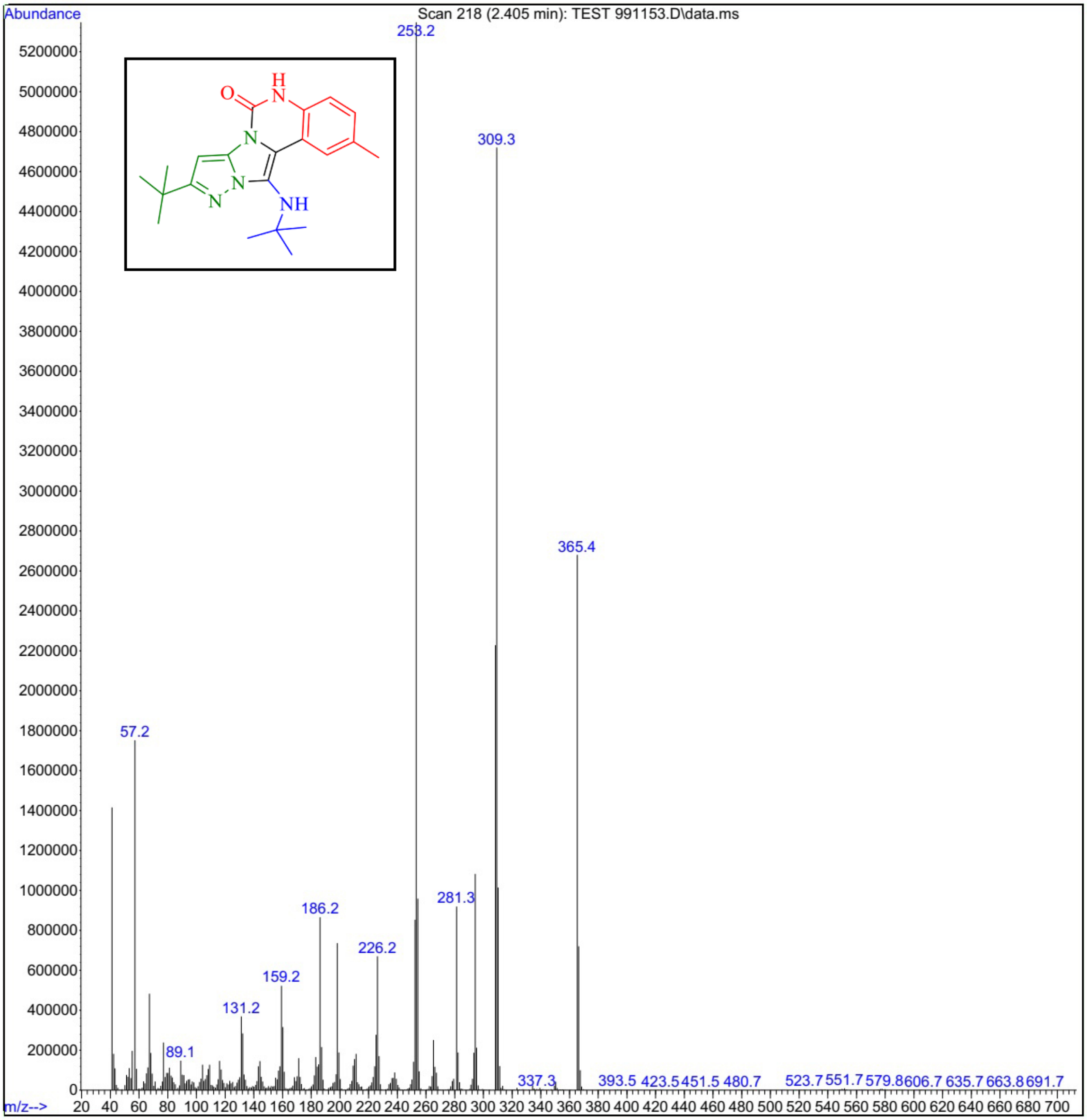

Figure S12. MASS spectrum of compound (4c).

^{1}H NMR, ^{13}C NMR, FT-IR and MASS of compound (4d)

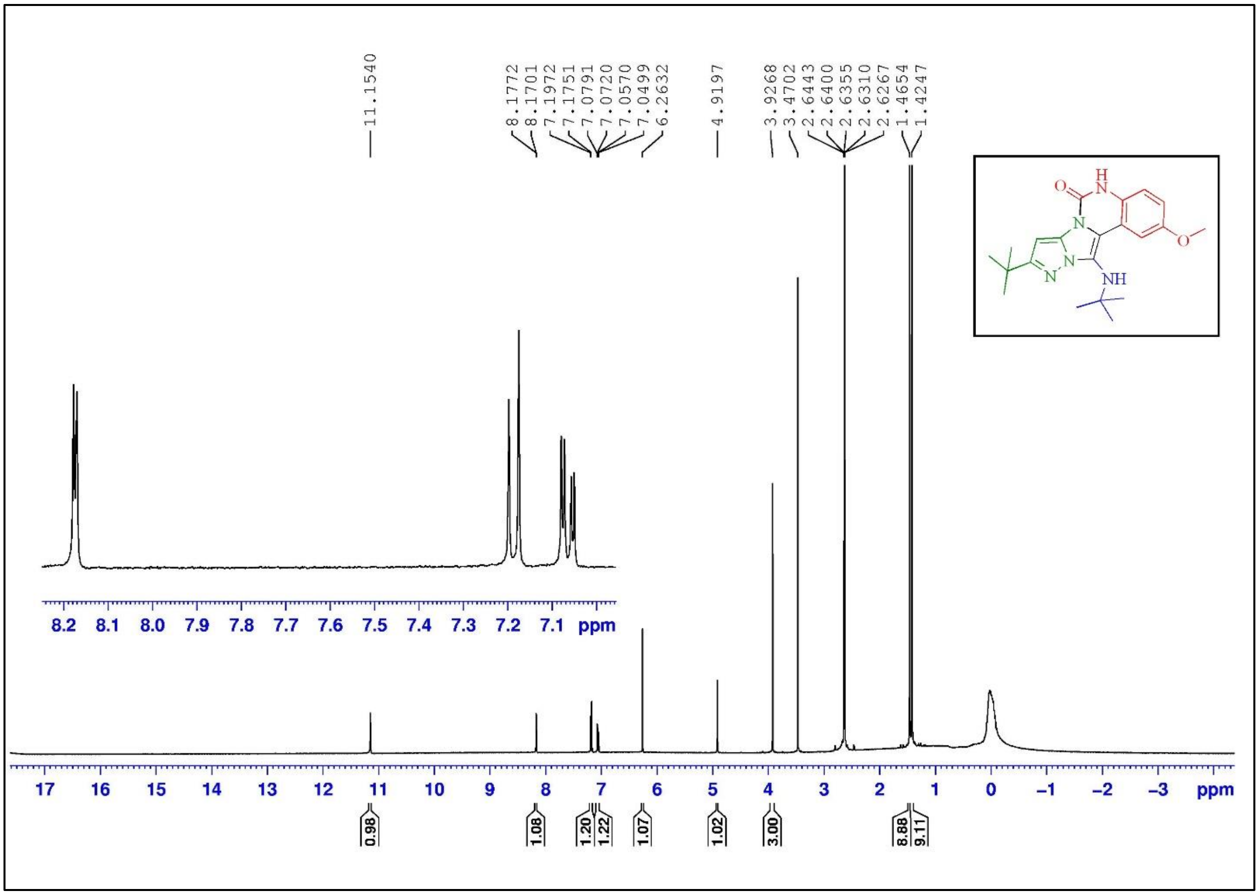

Figure S13. ^{1}H NMR spectrum of compound (4d) in DMSO.

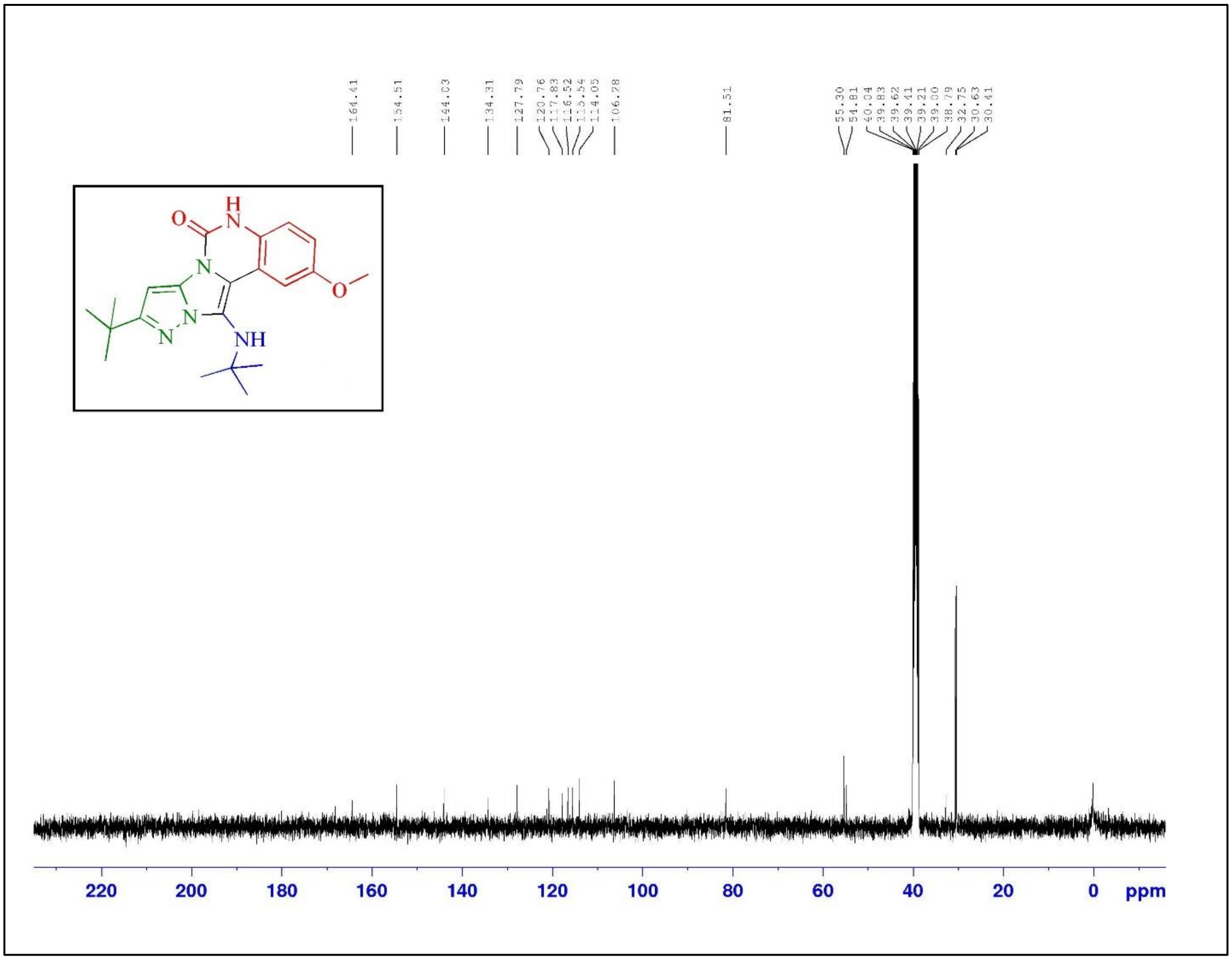

Figure S14. ^{13}C NMR spectrum of compound (4d) in DMSO.

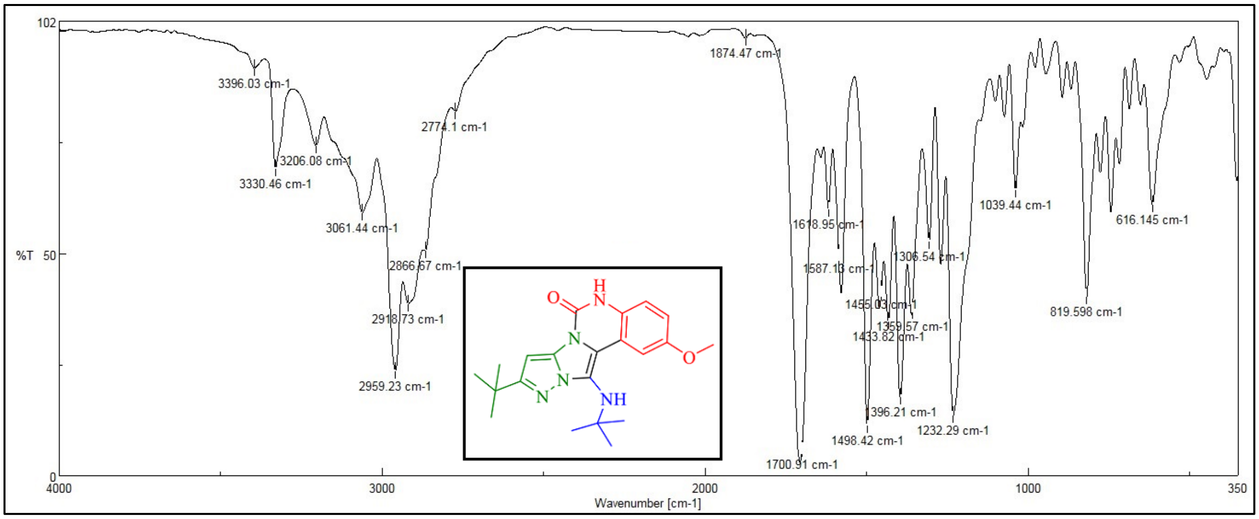

Figure S15. FT-IR spectrum of compound (4d).

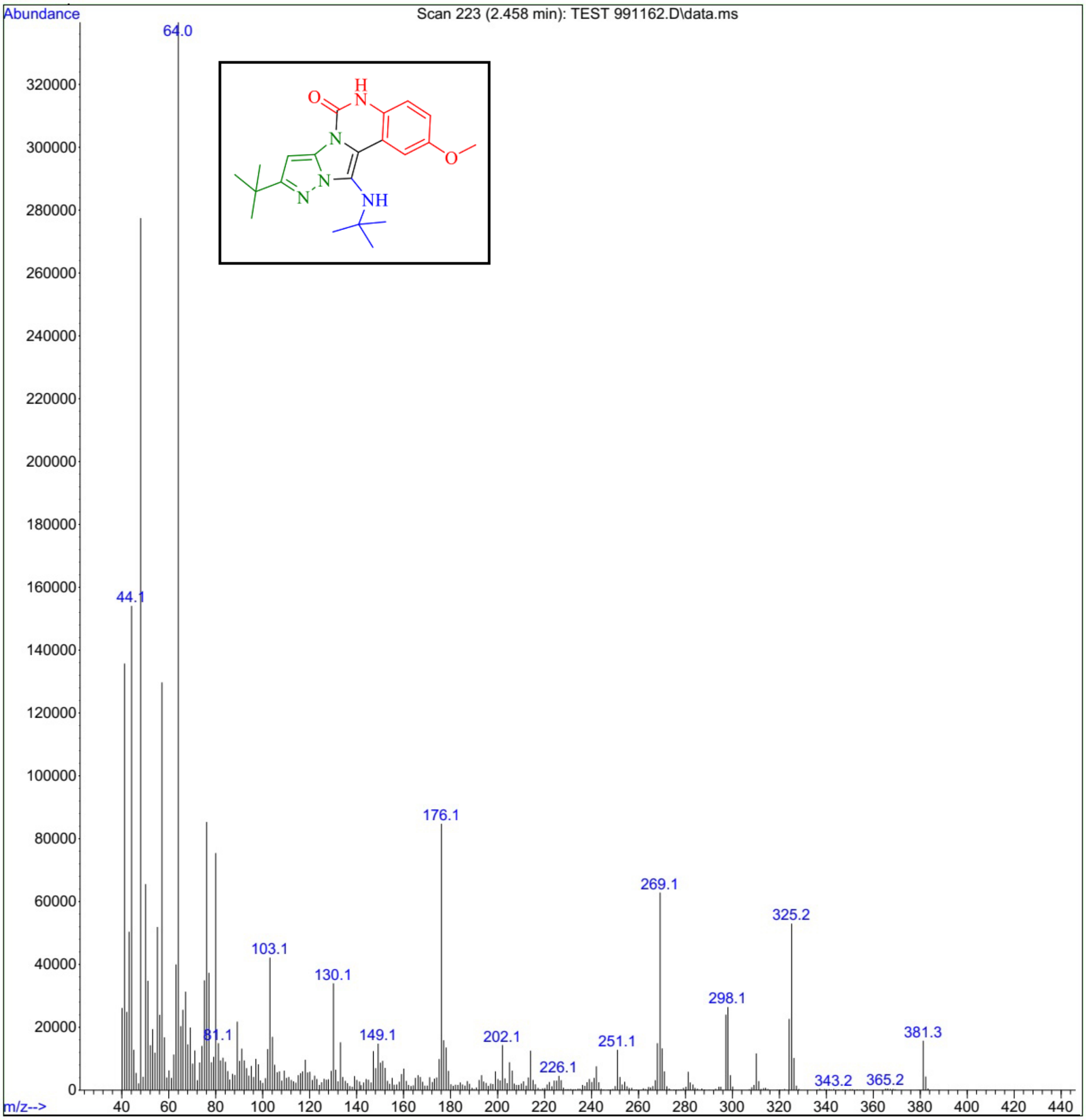

Figure S16. MASS spectrum of compound (4d).

^{1}H NMR, ^{13}C NMR, FT-IR and MASS of compound (4e)

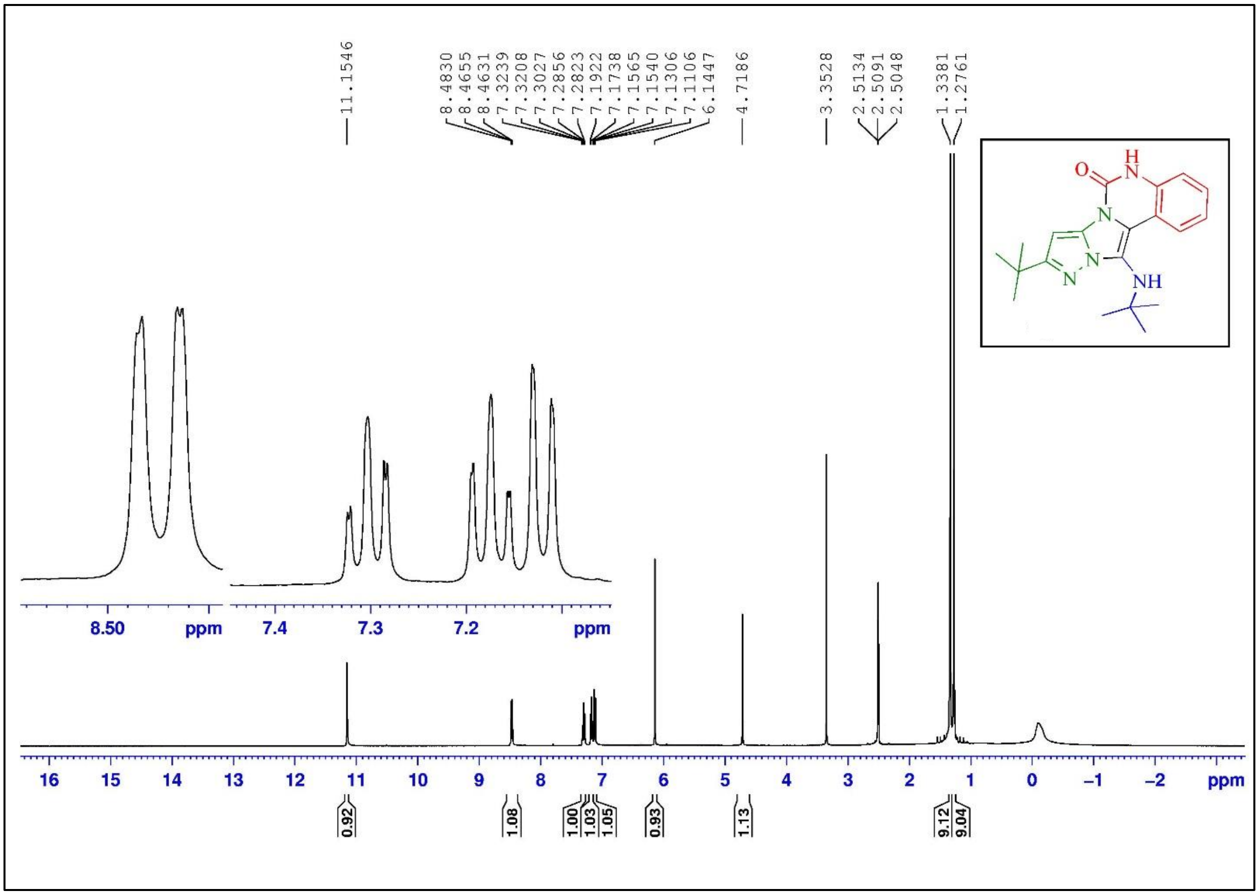

Figure S17. ^{1}H NMR spectrum of compound (4e) in DMSO.

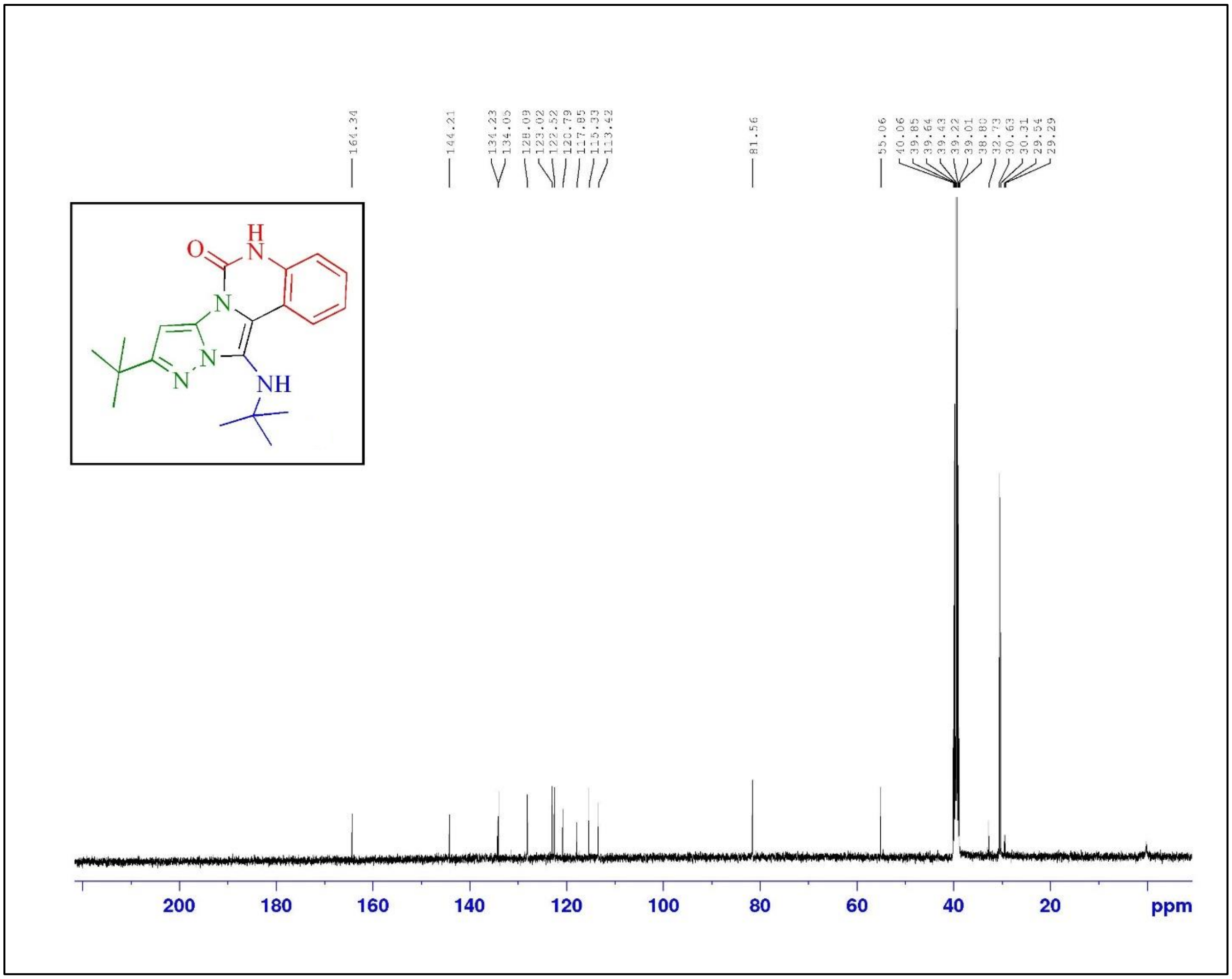

Figure S18. ^{13}C NMR spectrum of compound (4e) in DMSO.

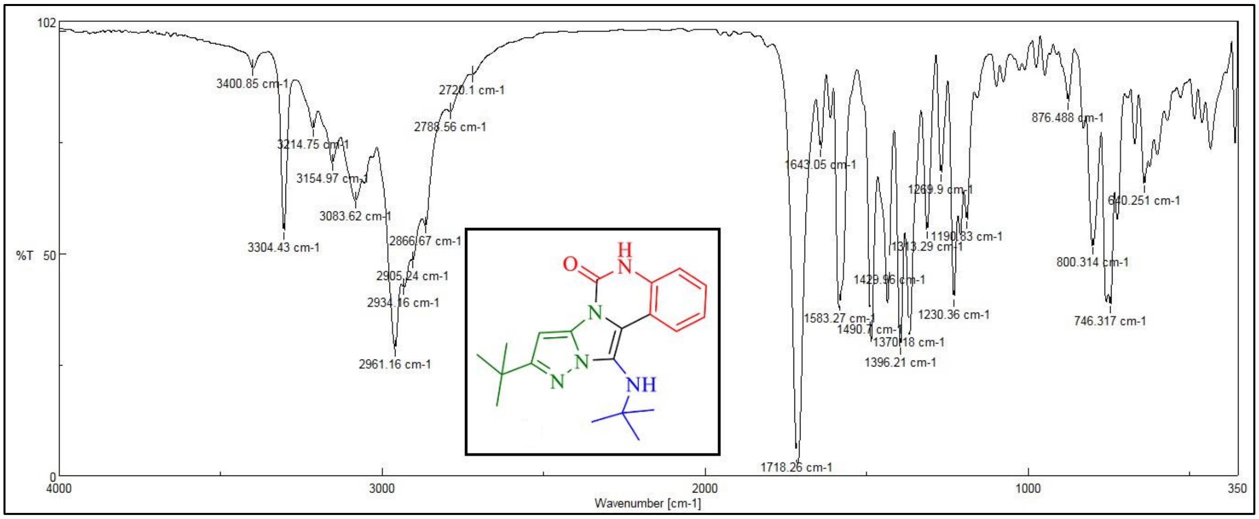

Figure S19. FT-IR spectrum of compound (4e).

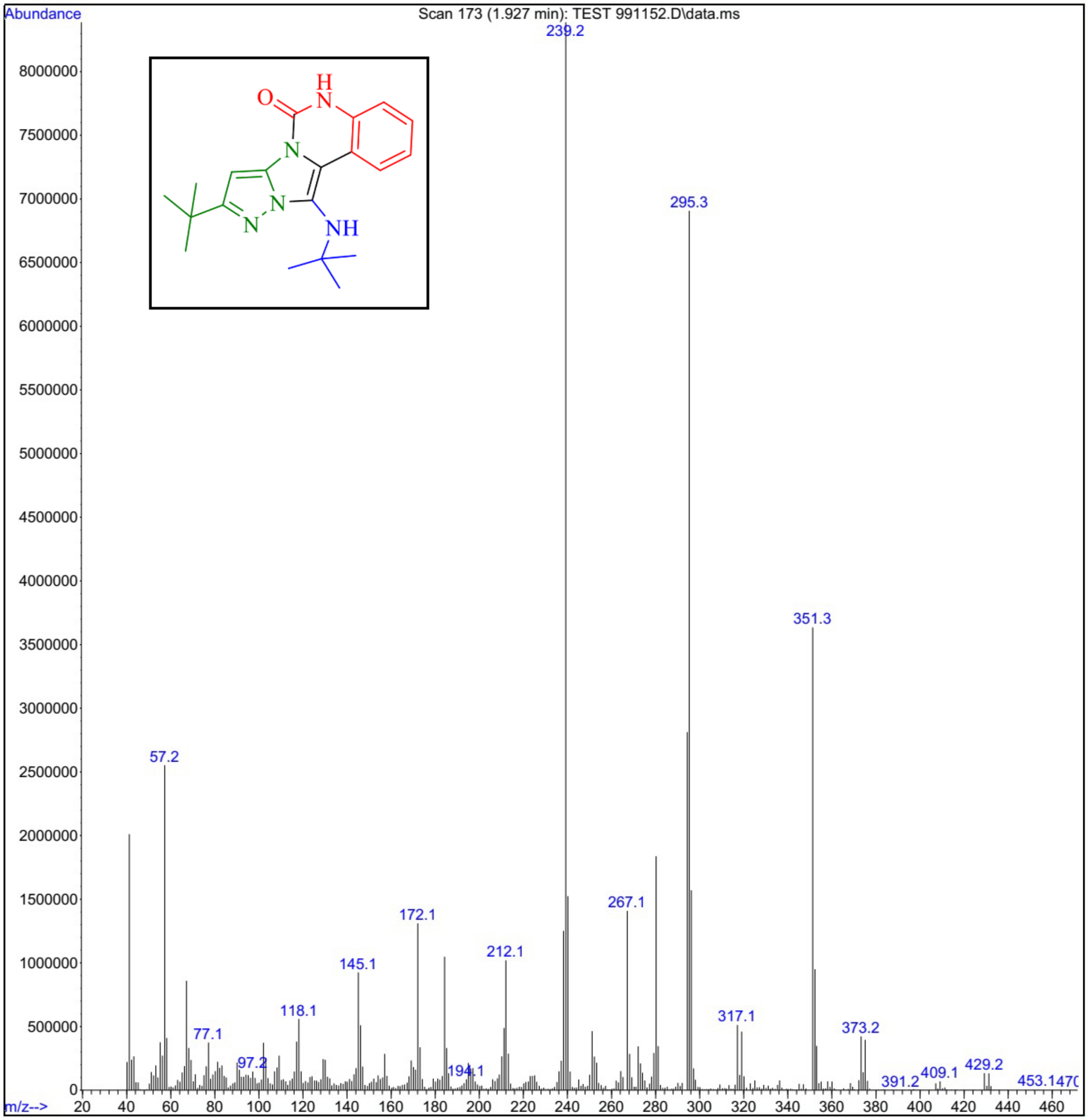

Figure S20. MASS spectrum of compound (4e).

^{1}H NMR, ^{13}C NMR, FT-IR and MASS of compound (4f)

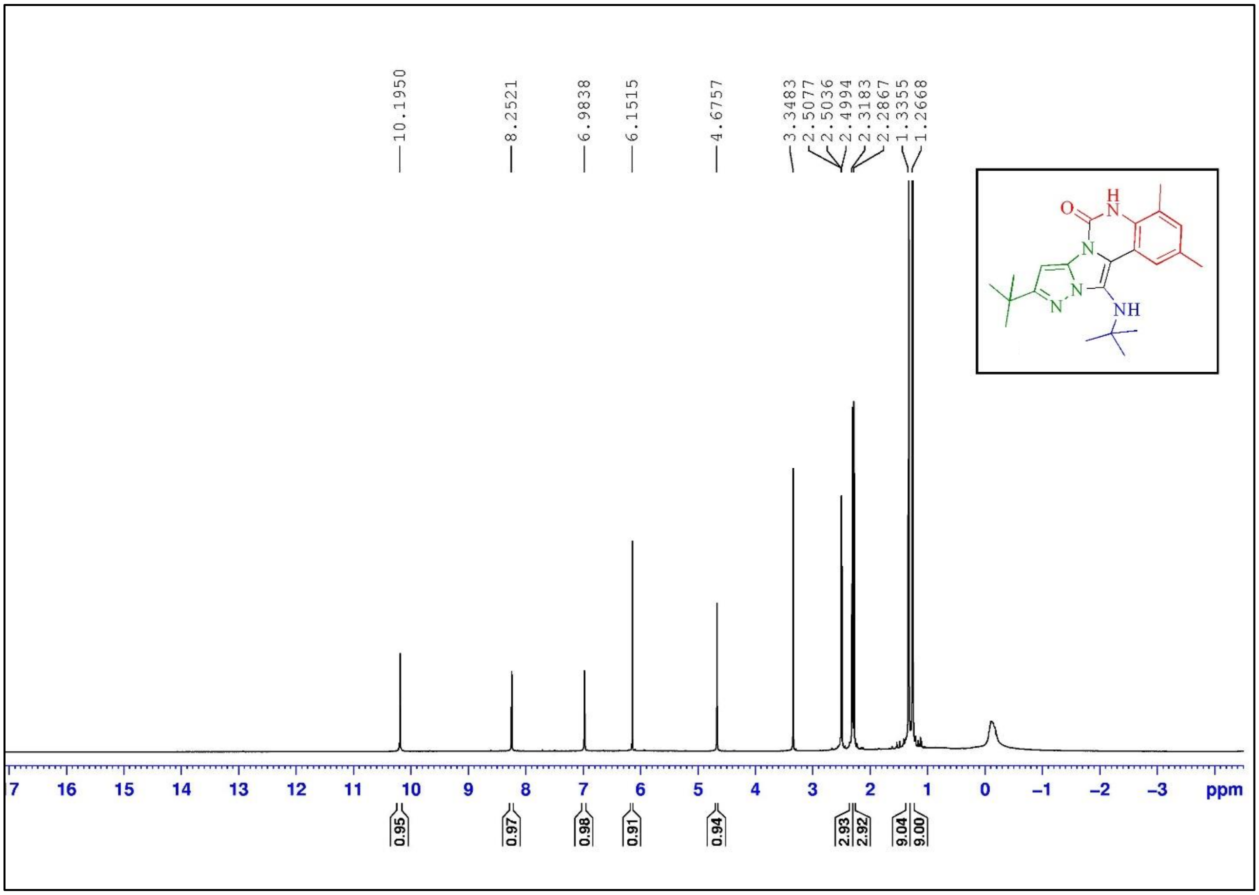

Figure S21. ^{1}H NMR spectrum of compound (4f) in DMSO.

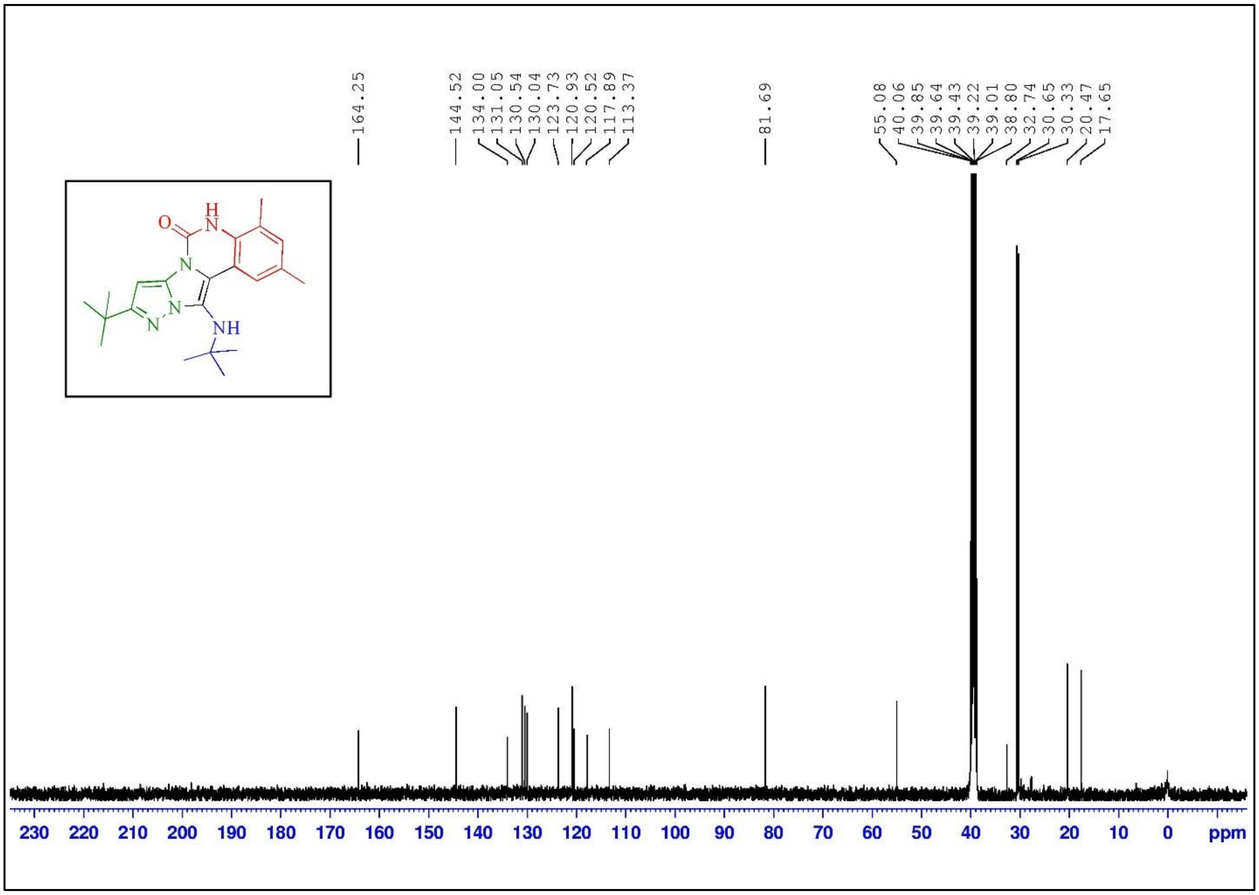

Figure S22. ^{13}C NMR spectrum of compound (4f) in DMSO.

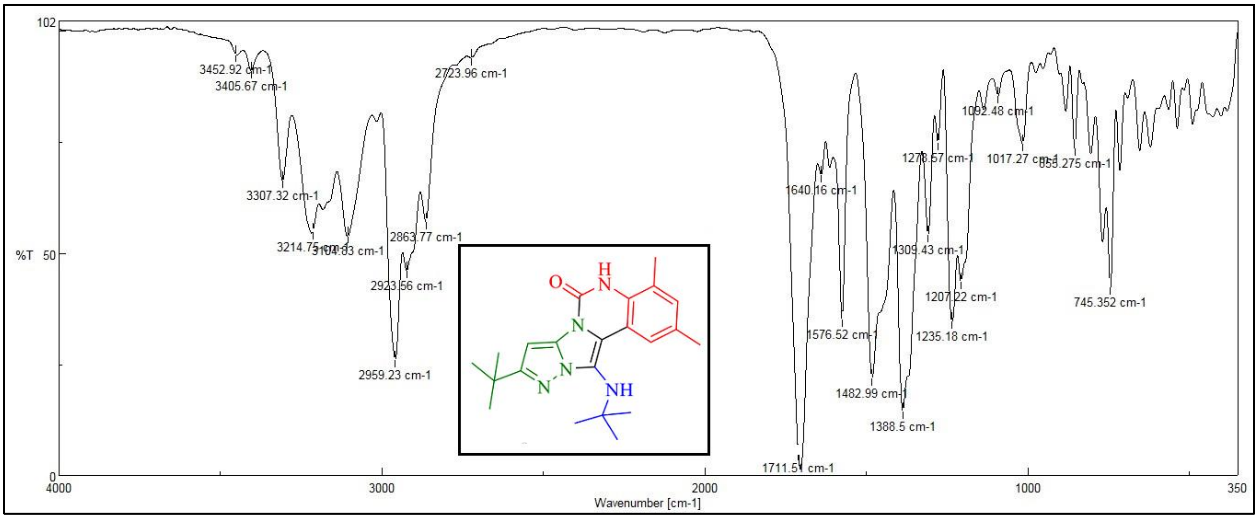

Figure S23. FT-IR spectrum of compound (4f).

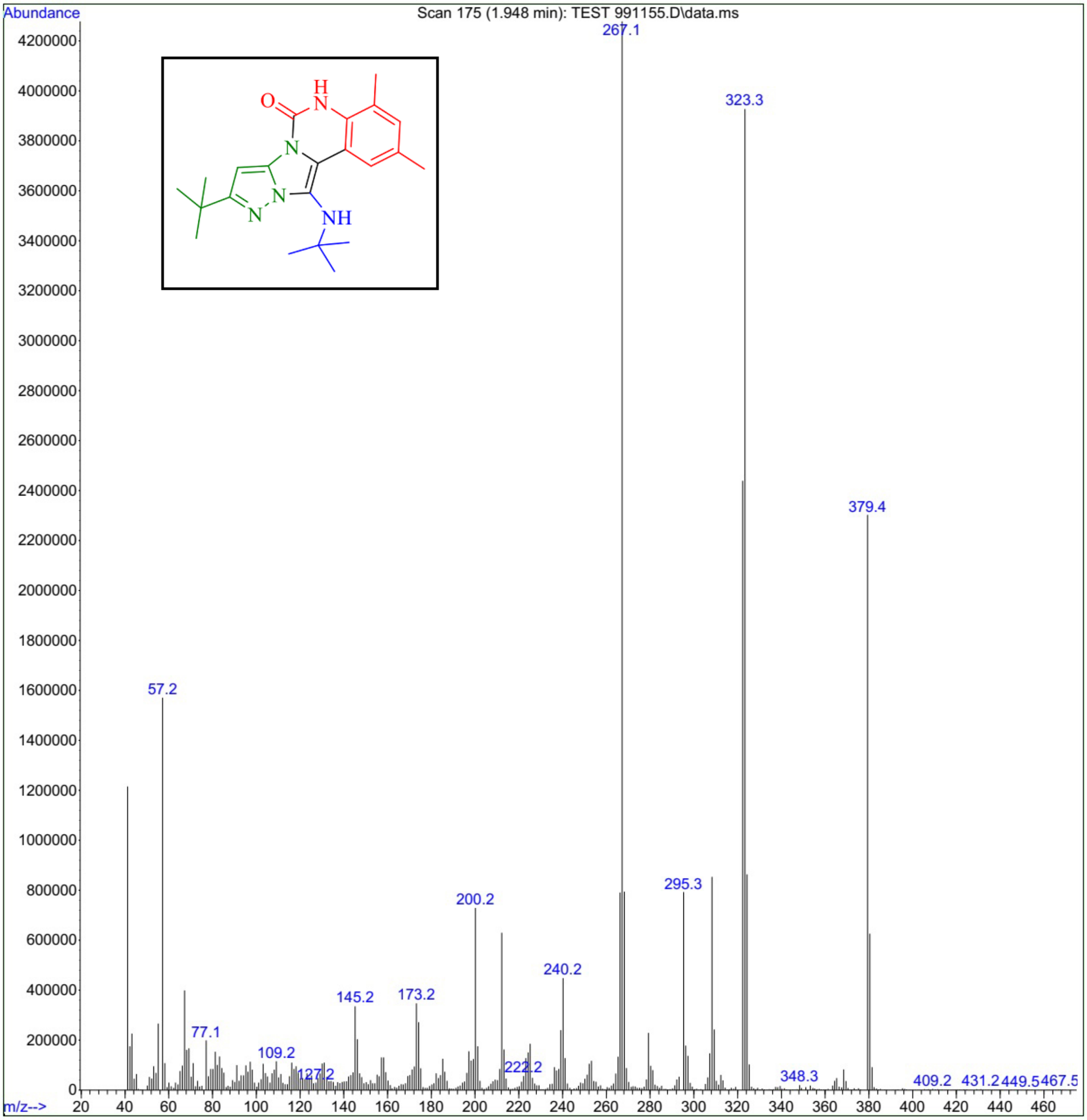

Figure S24. MASS spectrum of compound (4f).

^{1}H NMR, ^{13}C NMR, FT-IR and MASS of compound (4g)

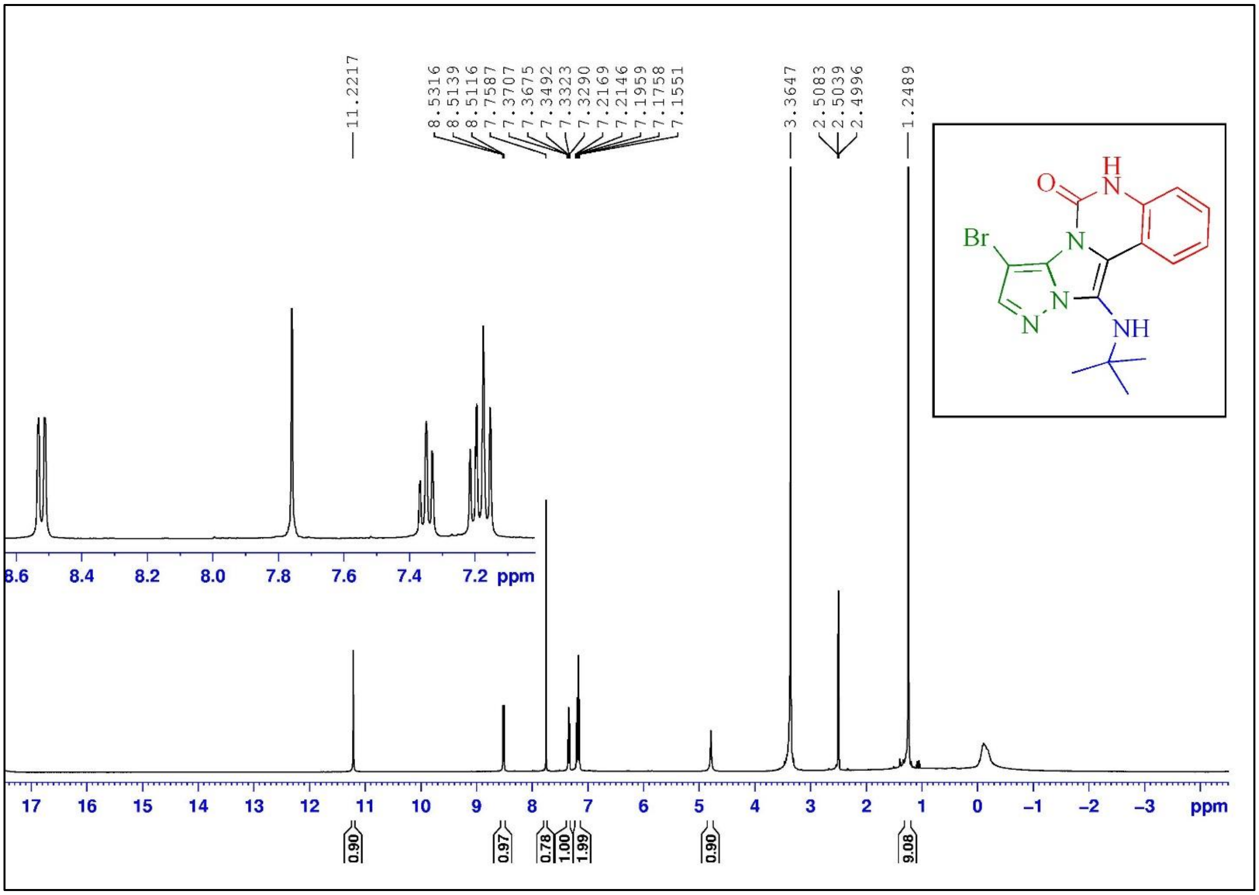

Figure S25. ^{1}H NMR spectrum of compound (4g) in DMSO.

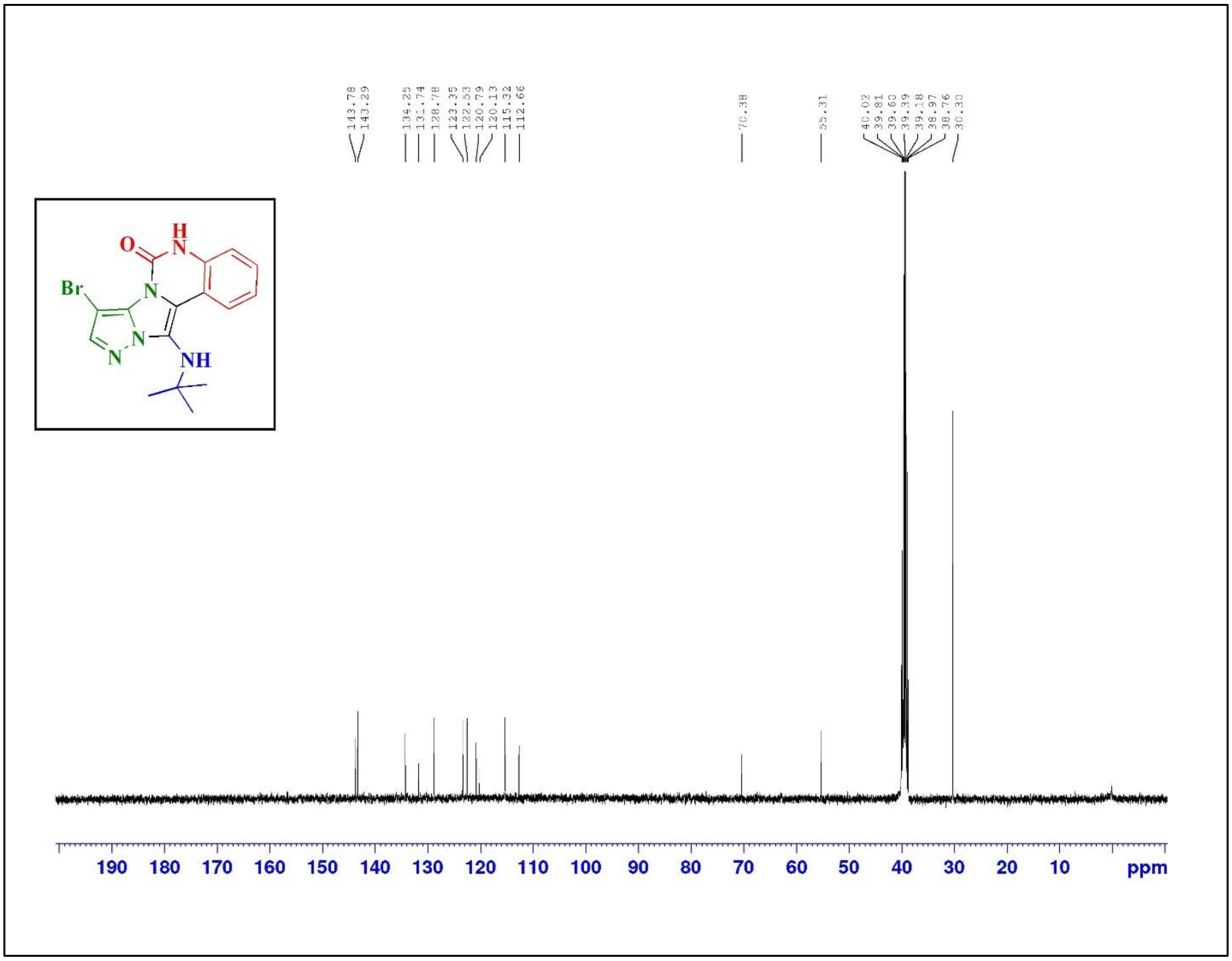

Figure S26. ^{13}C NMR spectrum of compound (4g) in DMSO.

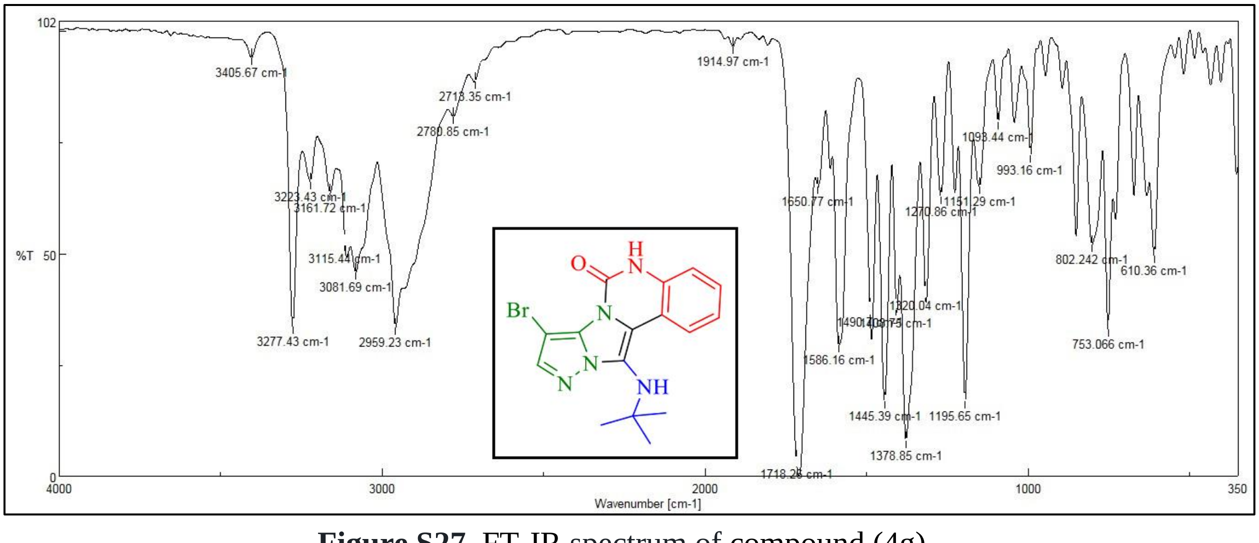

Figure S27. FT-IR spectrum of compound (4g).

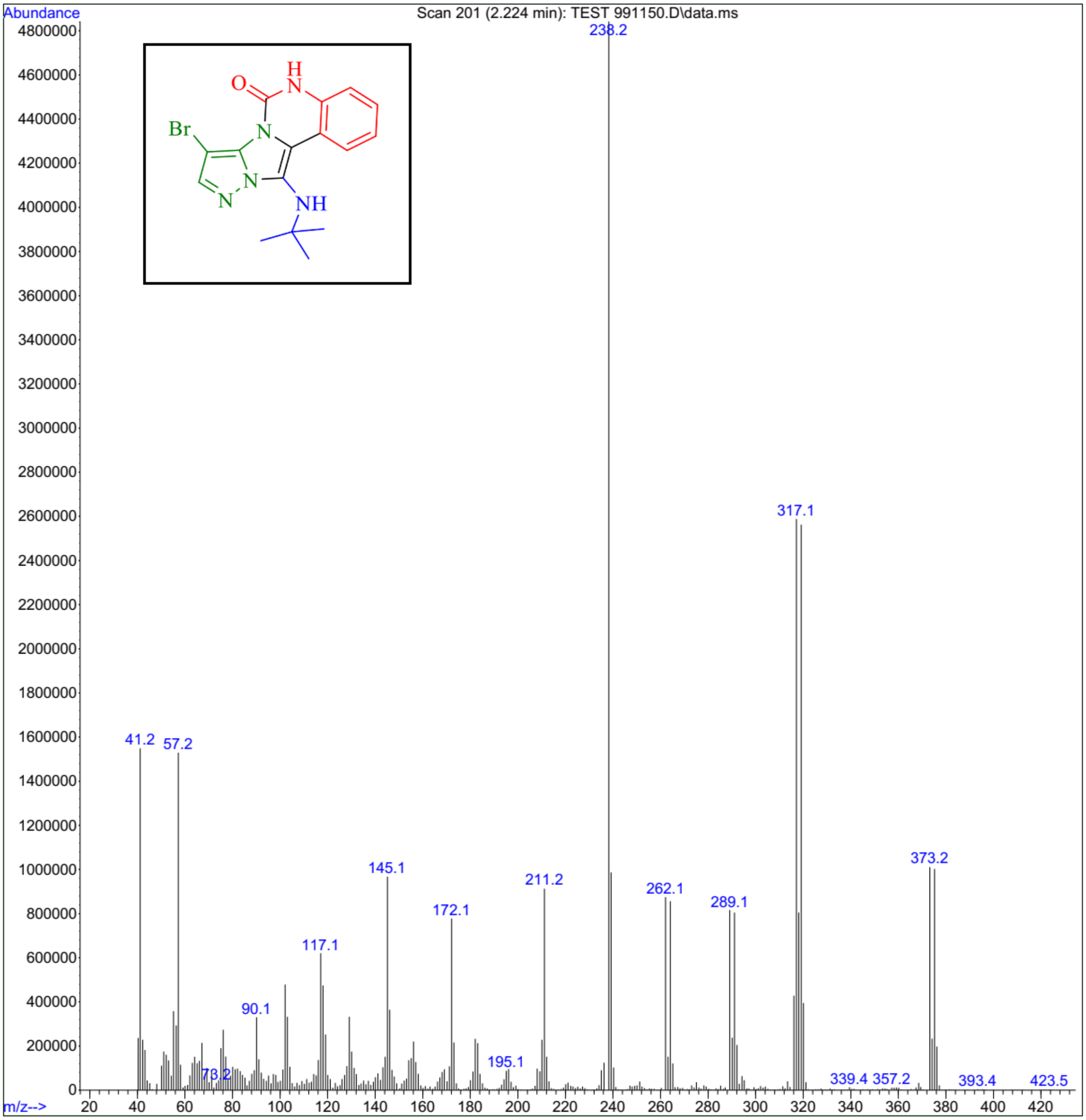

Figure S28. MASS spectrum of compound (4g).

^{1}H NMR, ^{13}C NMR, FT-IR and MASS of compound (4h)

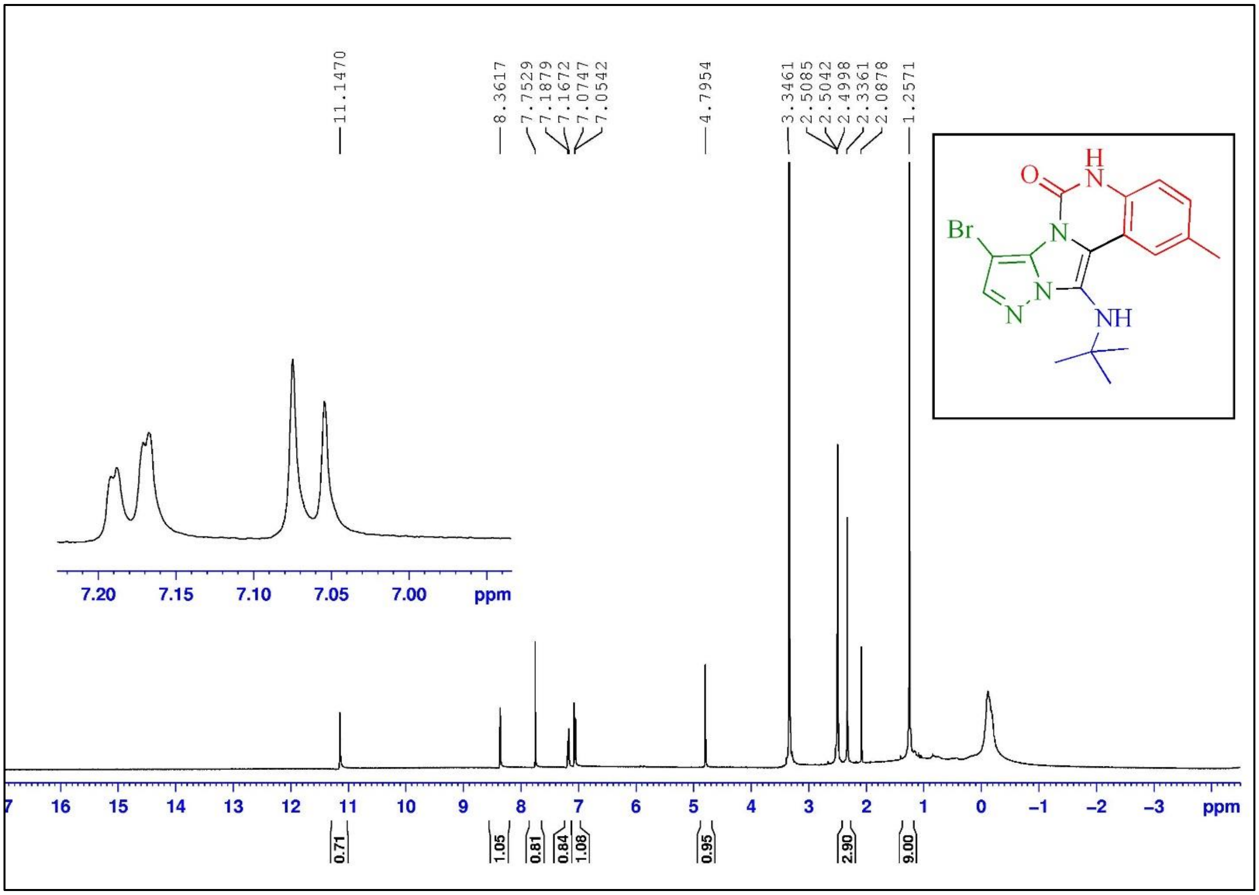

Figure S29. ^{1}H NMR spectrum of compound (4h) in DMSO.

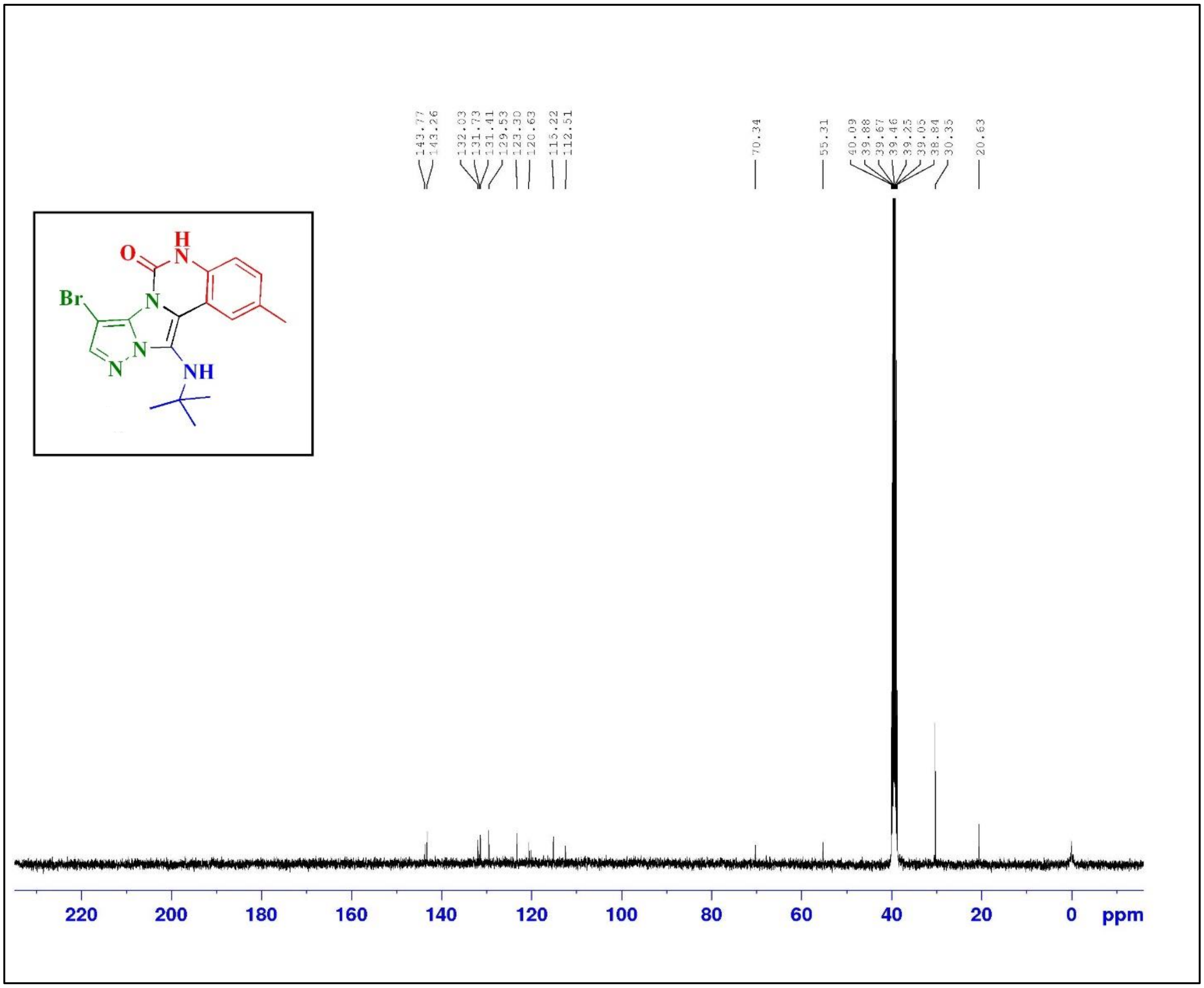

Figure S30. ^{13}C NMR spectrum of compound (4h) in DMSO.

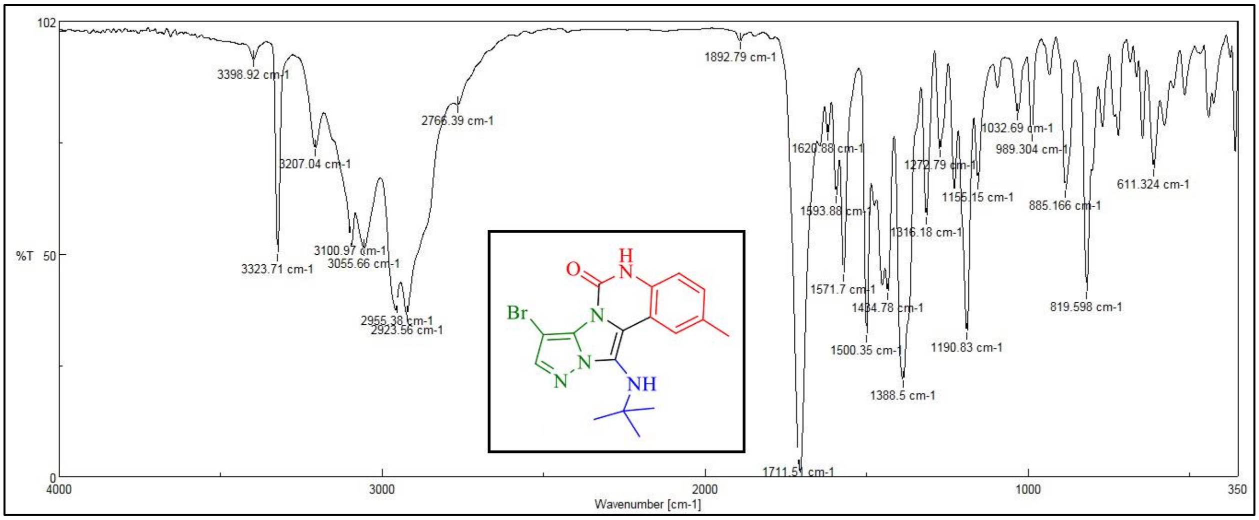

Figure S31. FT-IR spectrum of compound (4h).

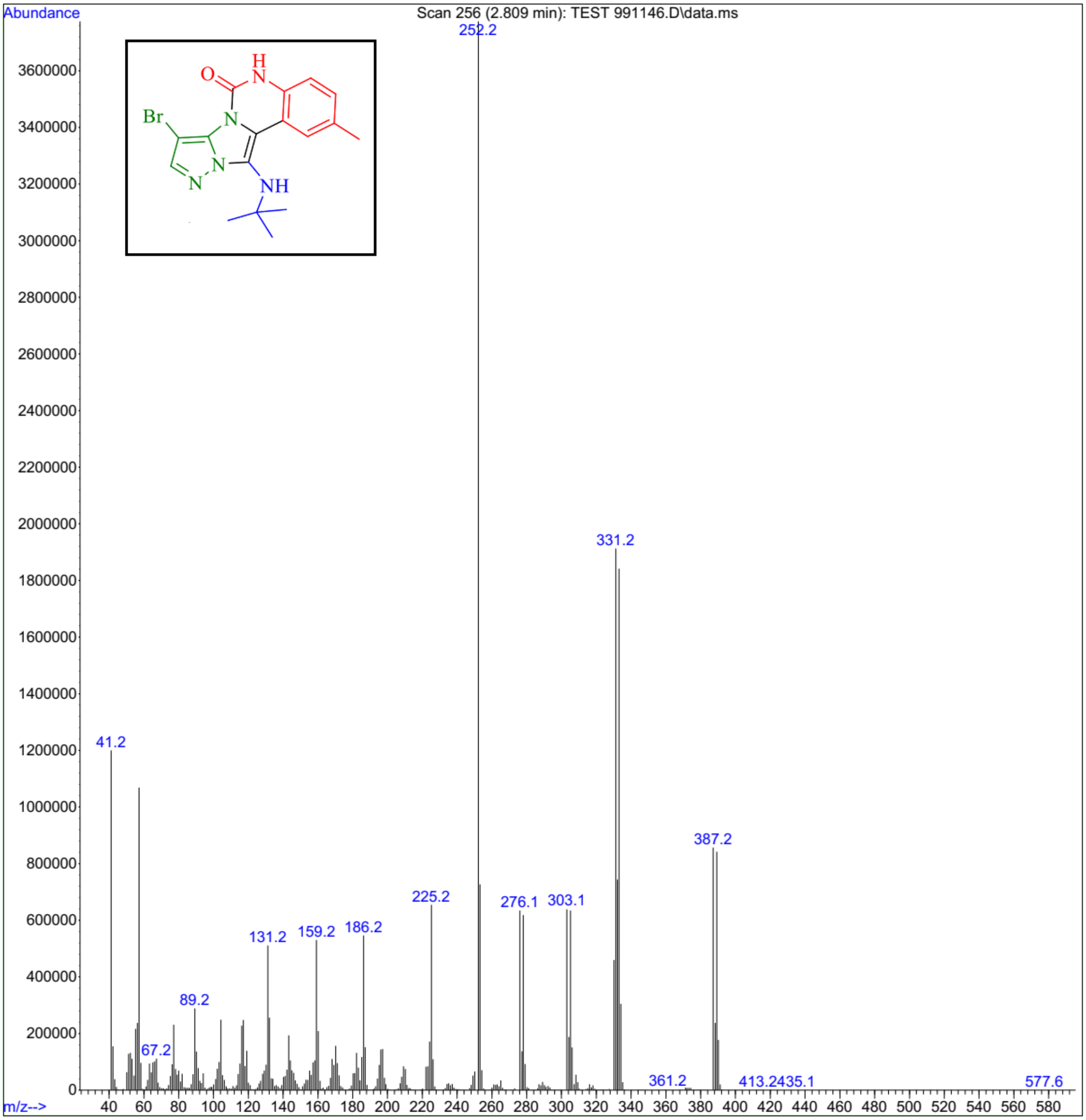

Figure S32. MASS spectrum of compound (4h).

^{1}H NMR, ^{13}C NMR, FT-IR and MASS of compound (4i)

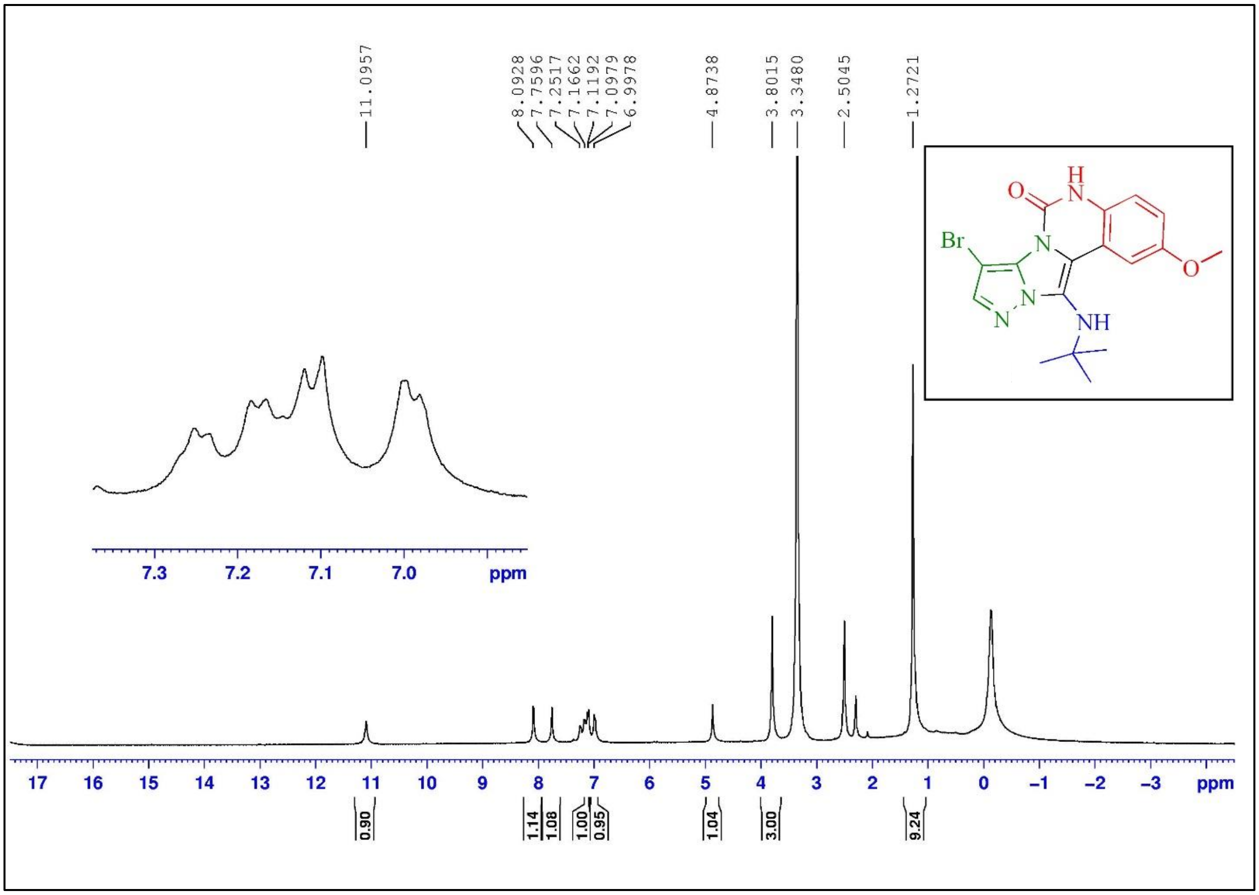

Figure S33. ^{1}H NMR spectrum of compound (4i) in DMSO.

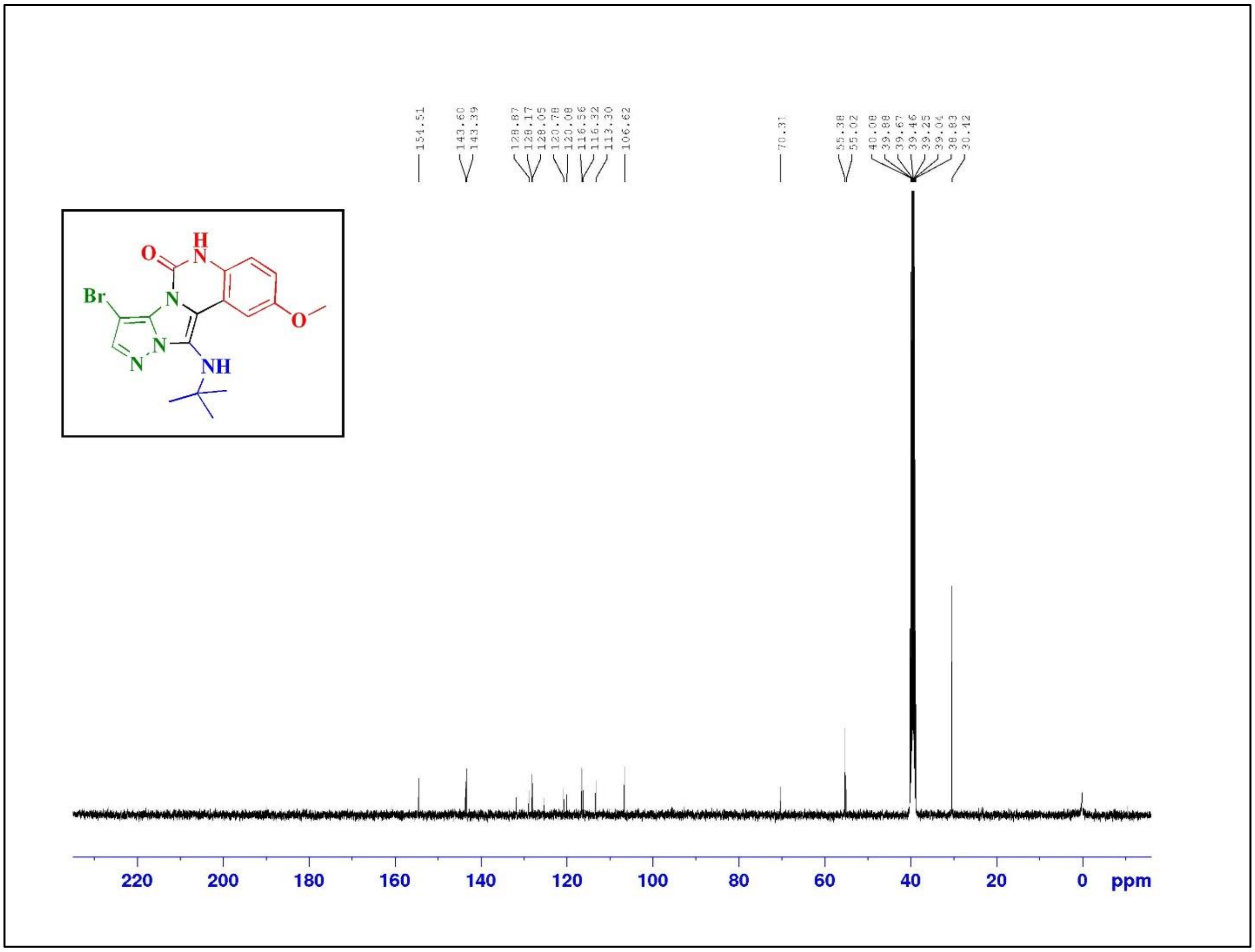

Figure S34. ^{13}C NMR spectrum of compound (4i) in DMSO.

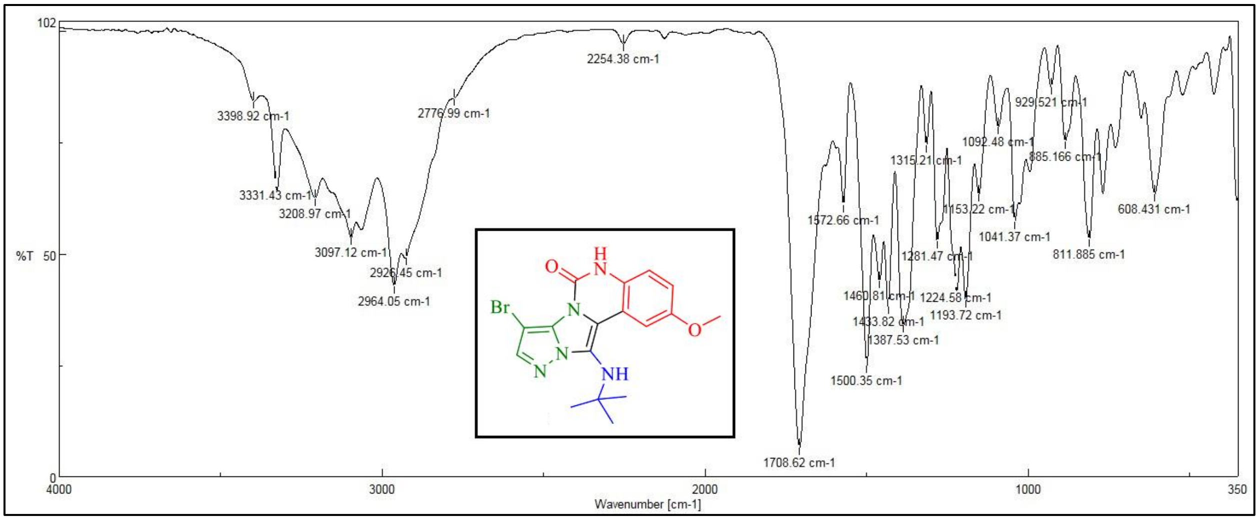

Figure S35. FT-IR spectrum of compound (4i).

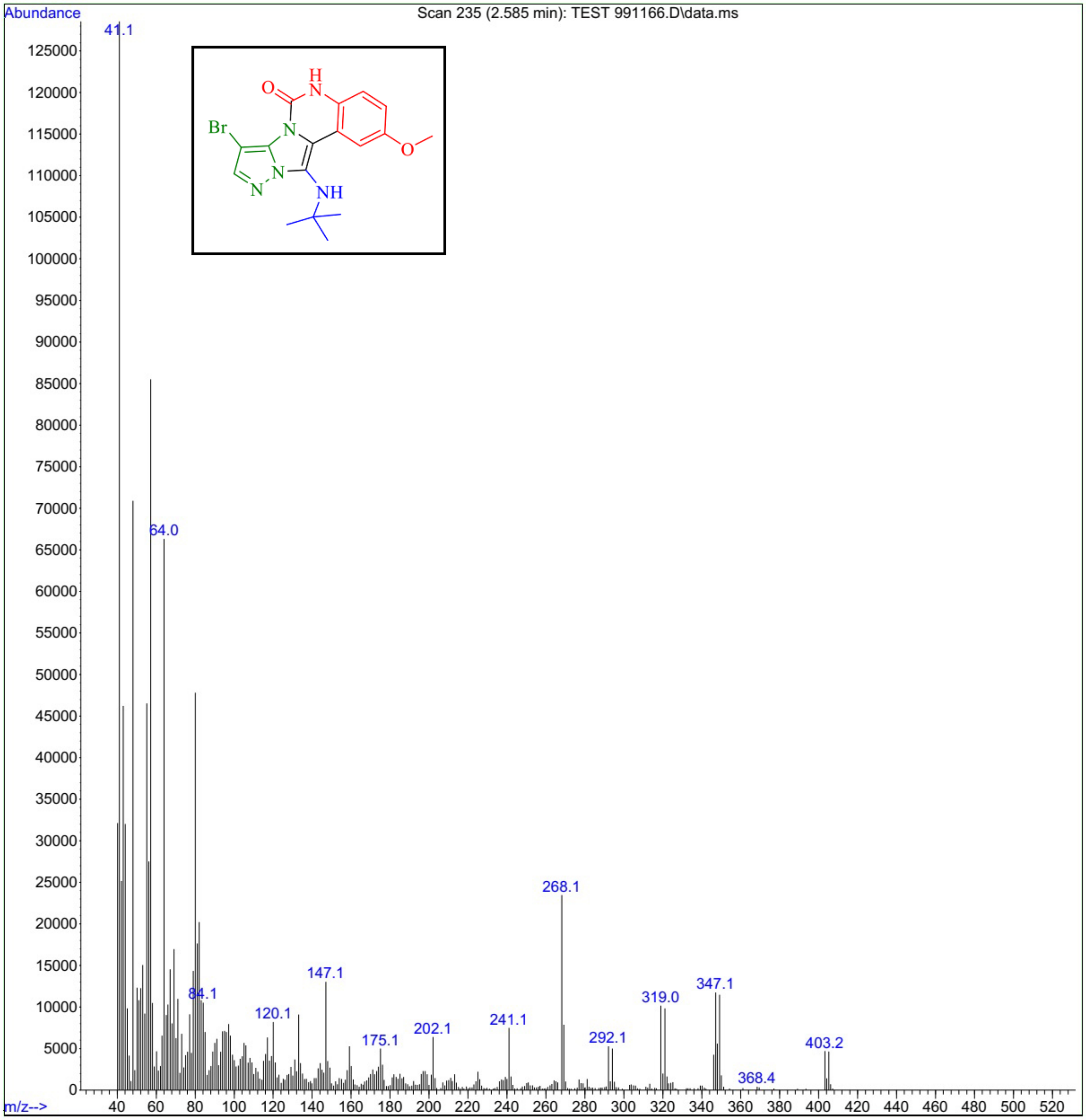

Figure S36. MASS spectrum of compound (4i).

^{1}H NMR, ^{13}C NMR, FT-IR and MASS of compound (4j)

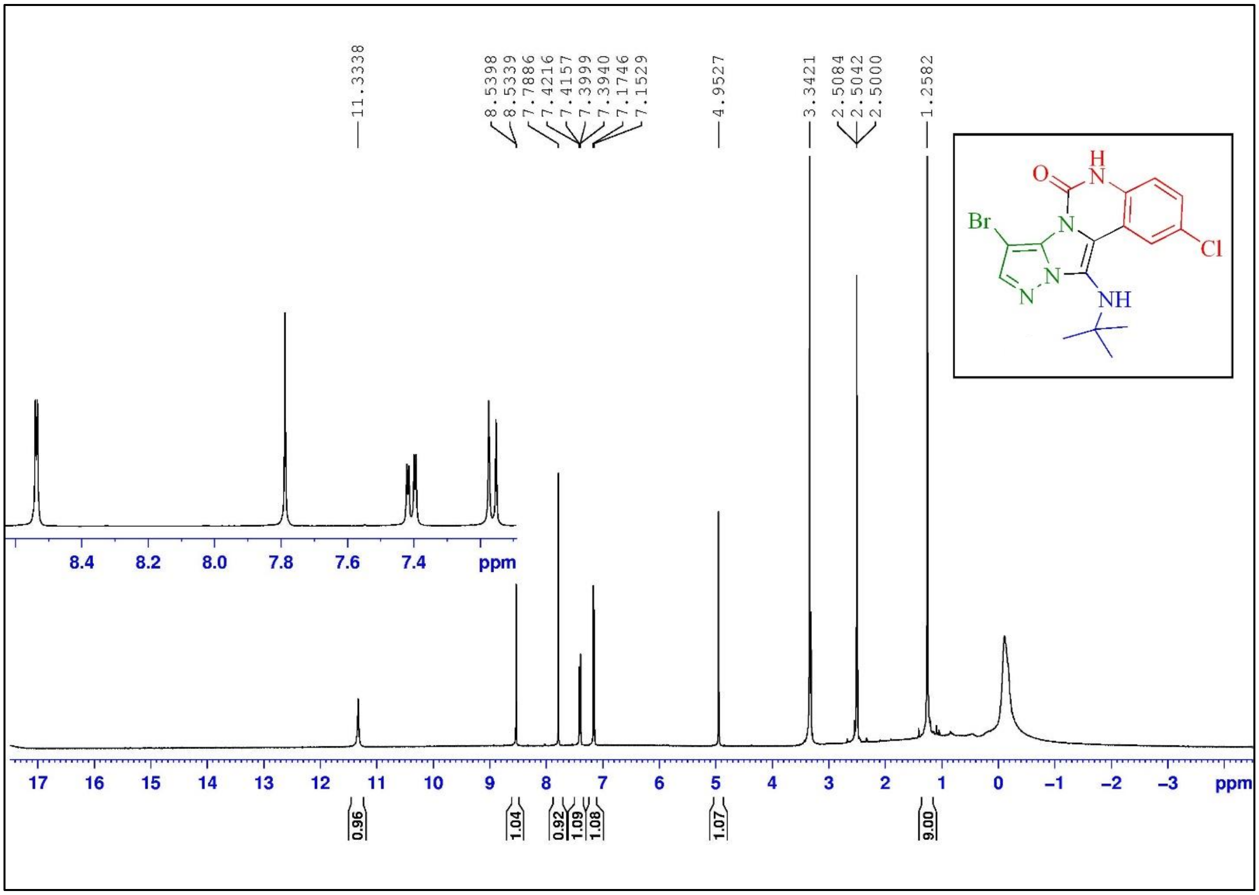

Figure S37. ^{1}H NMR spectrum of compound (4j) in DMSO.

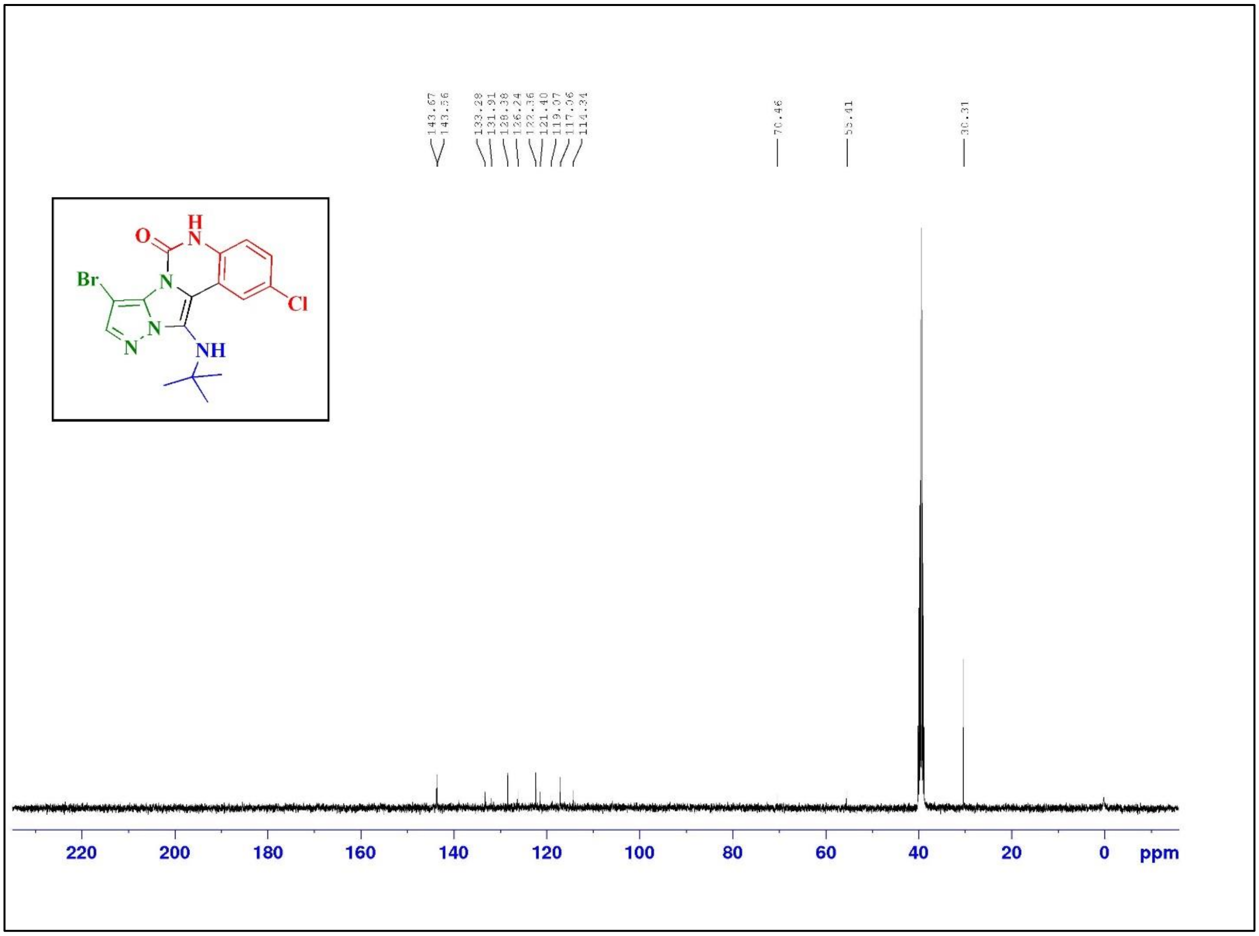

Figure S38. ^{13}C NMR spectrum of compound (4j) in DMSO.

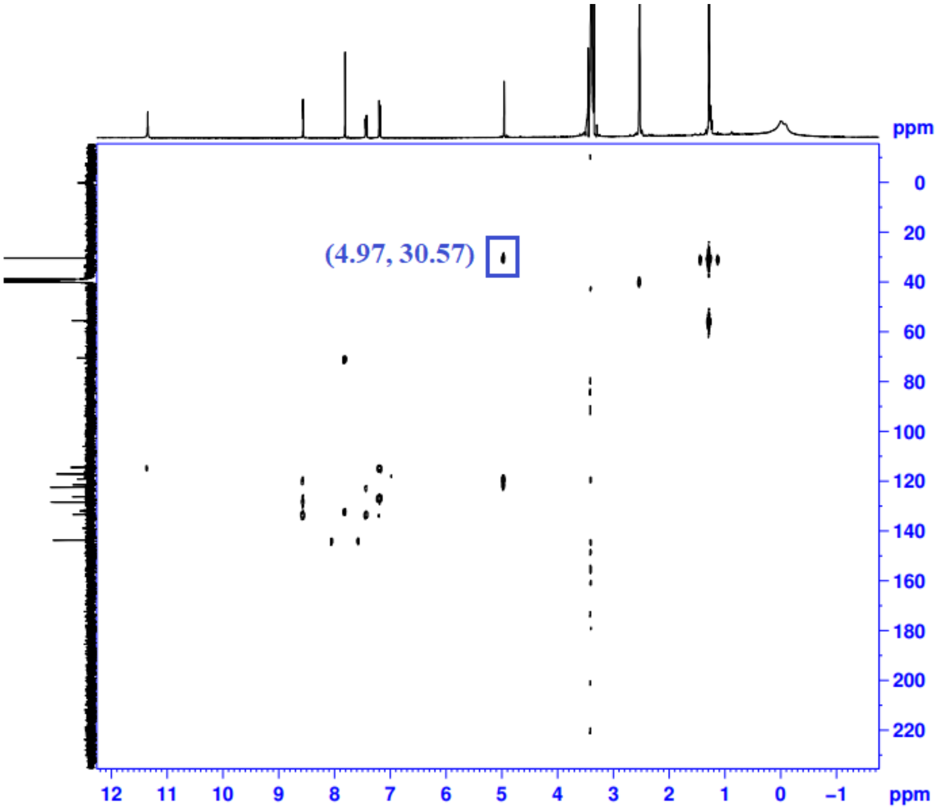

Figure S39. 2D HMBC spectrum of compound (4j) in DMSO.

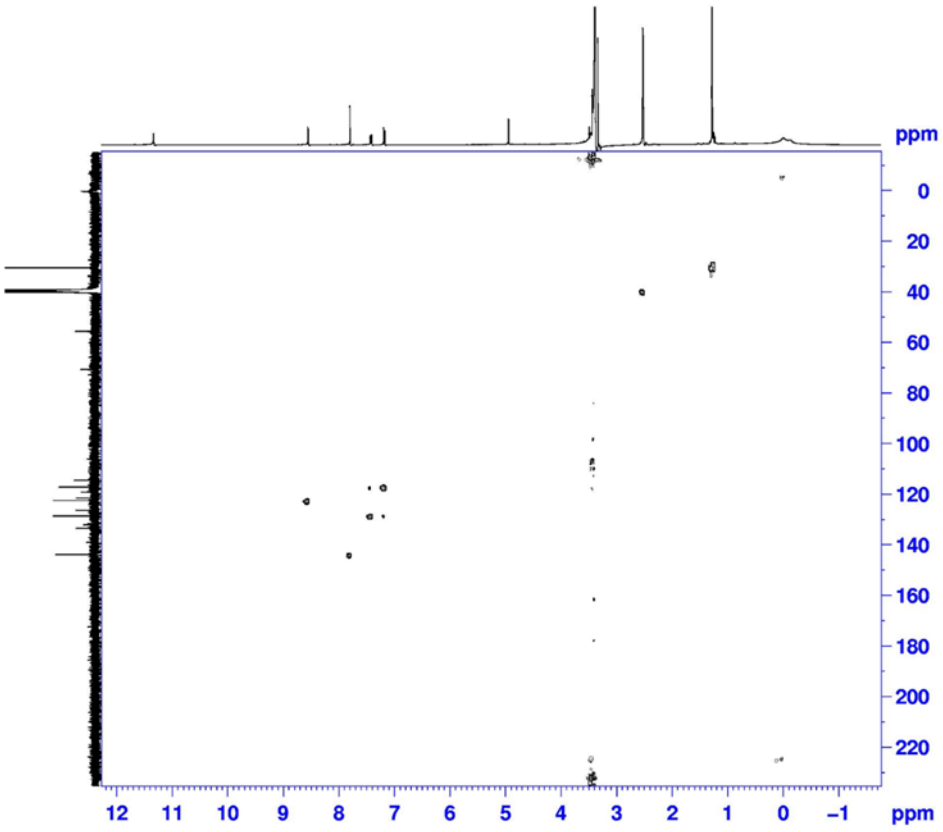

Figure S40. 2D HMBC spectrum of compound (4j) in DMSO.

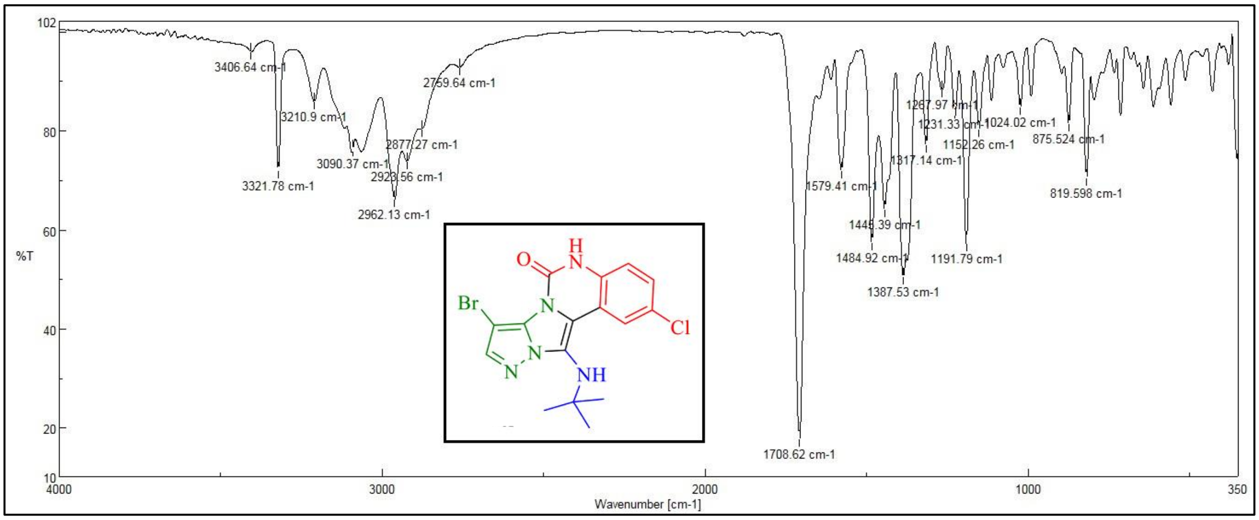

Figure S41. FT-IR spectrum of compound (4j).

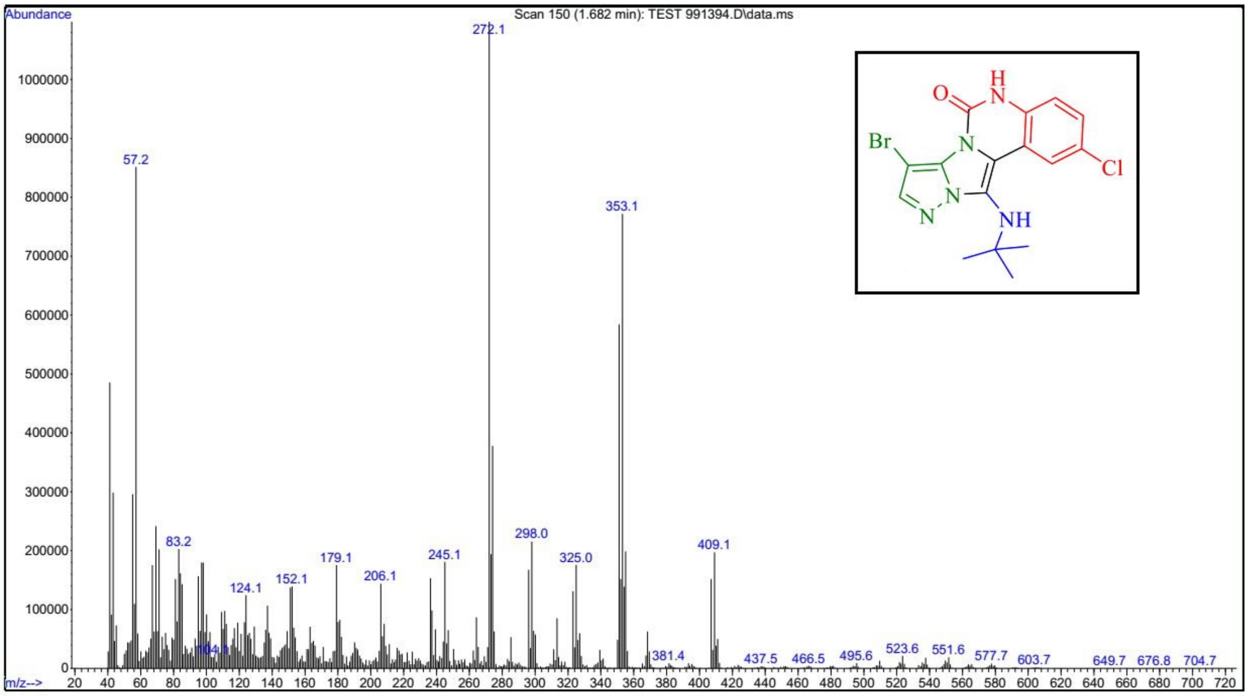

Figure S42. MASS spectrum of compound (4j).

^{1}H NMR, ^{13}C NMR, FT-IR and MASS of compound (4k)

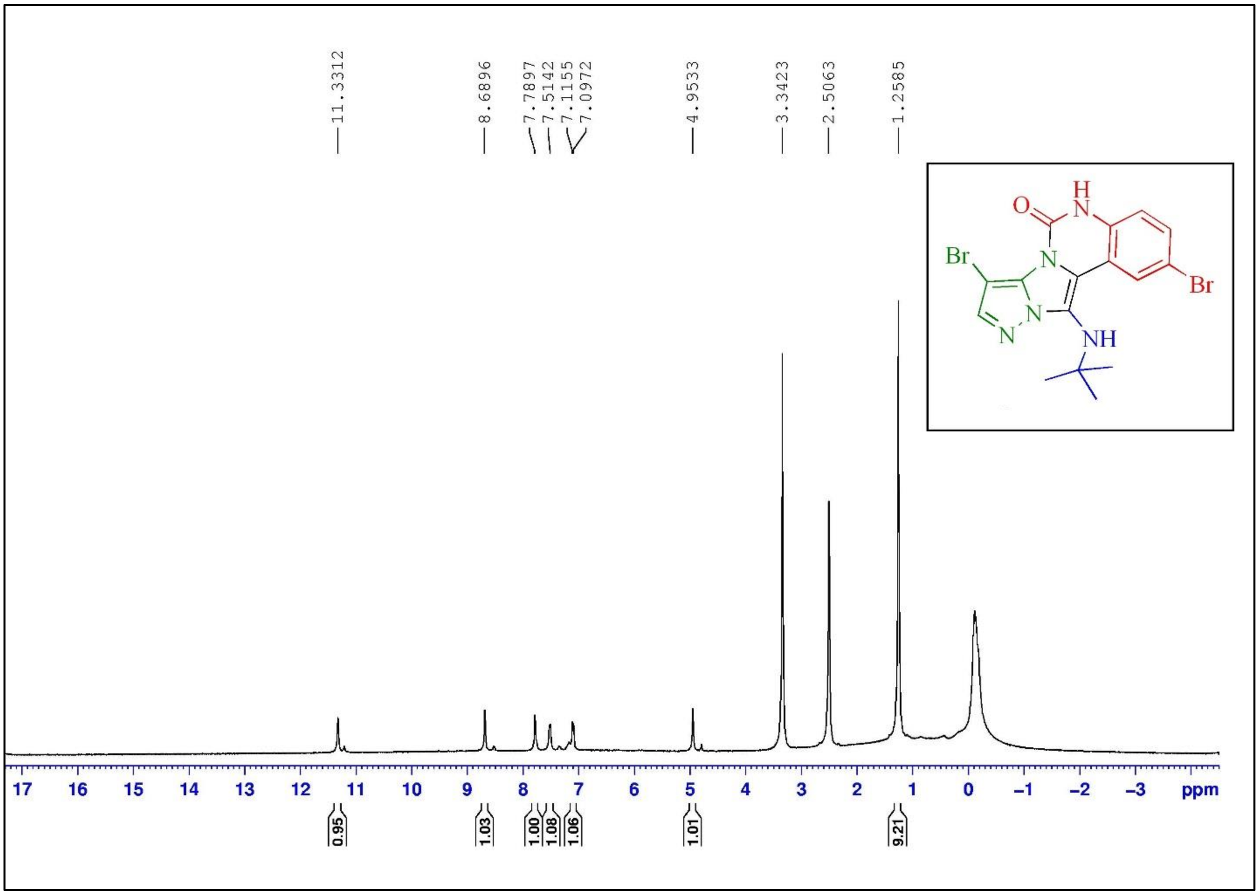

Figure S43. ^{1}H NMR spectrum of compound (4k) in DMSO.

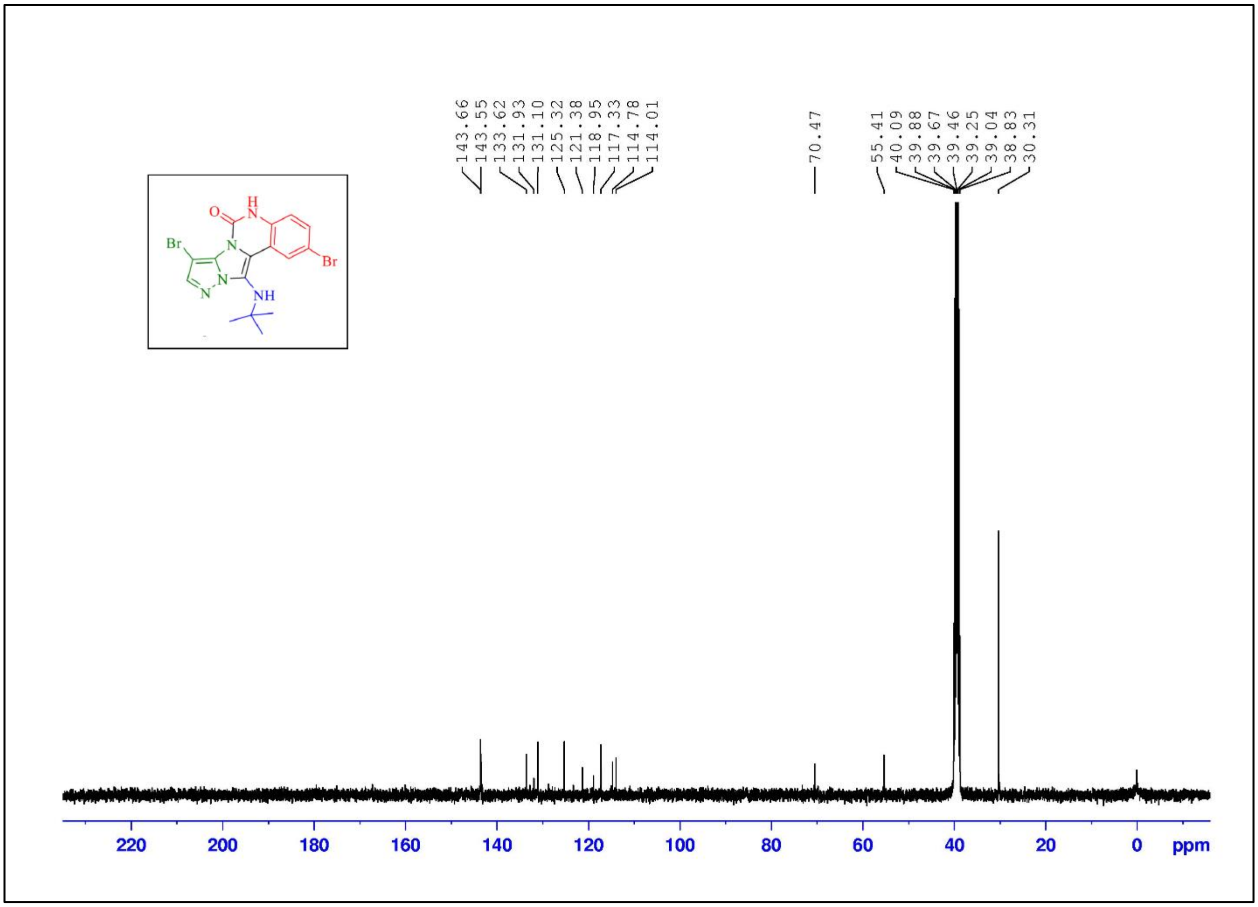

Figure S44. ^{13}C NMR spectrum of compound (4k) in DMSO.

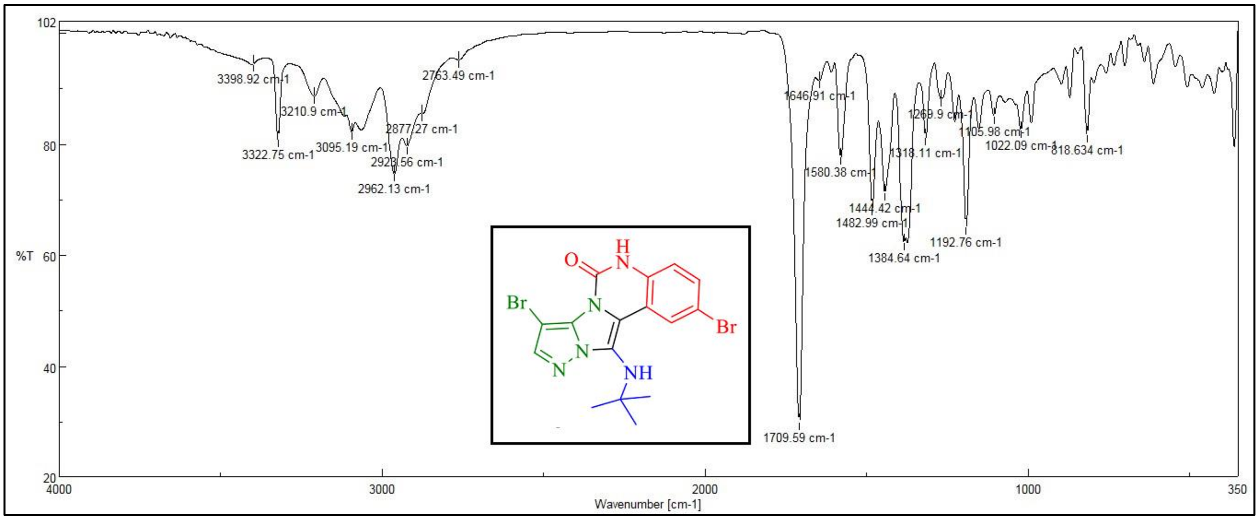

Figure S45. FT-IR spectrum of compound (4k).

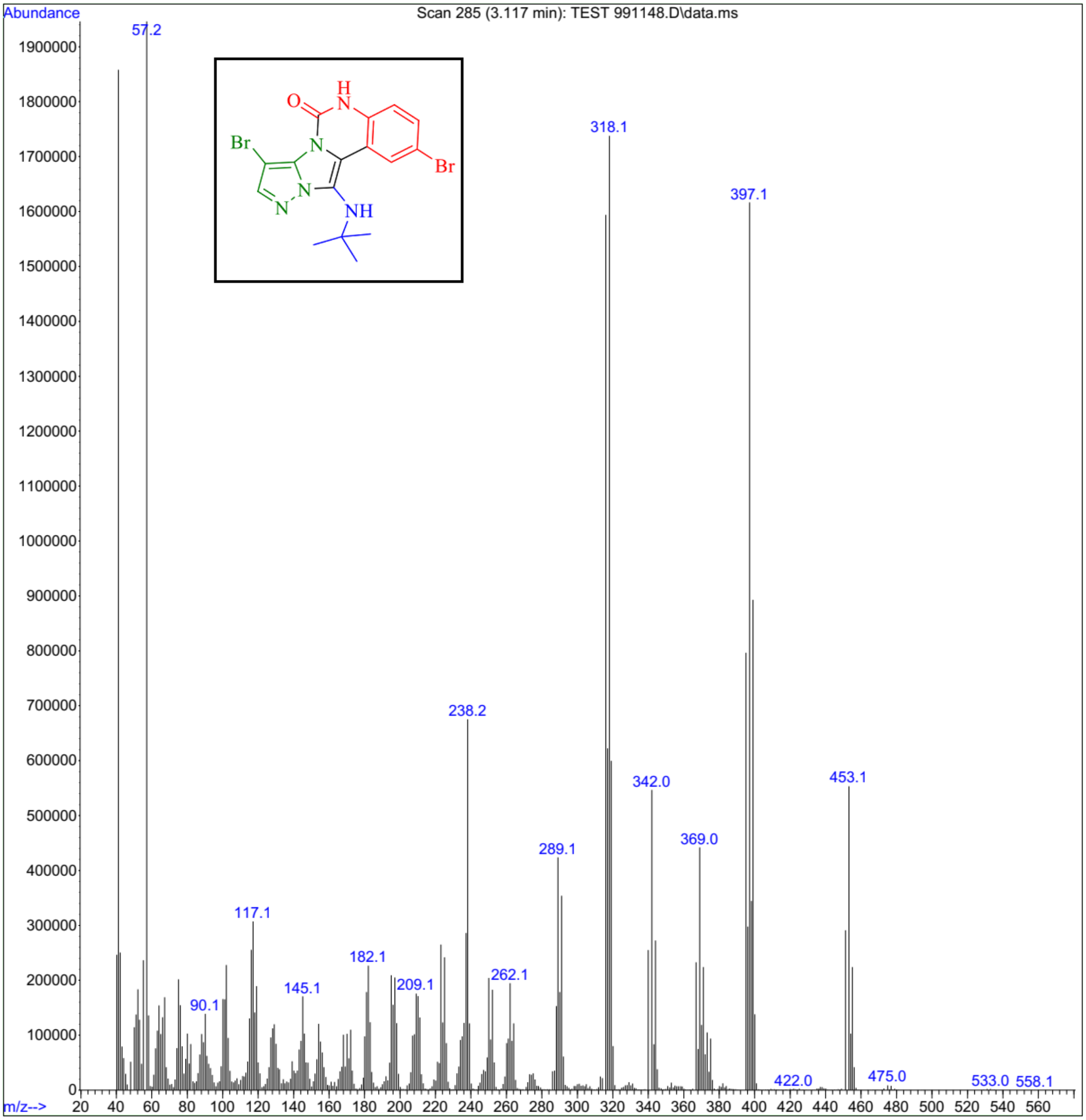

Figure S46. MASS spectrum of compound (4k).

^{1}H NMR, ^{13}C NMR, FT-IR and MASS of compound (4l)

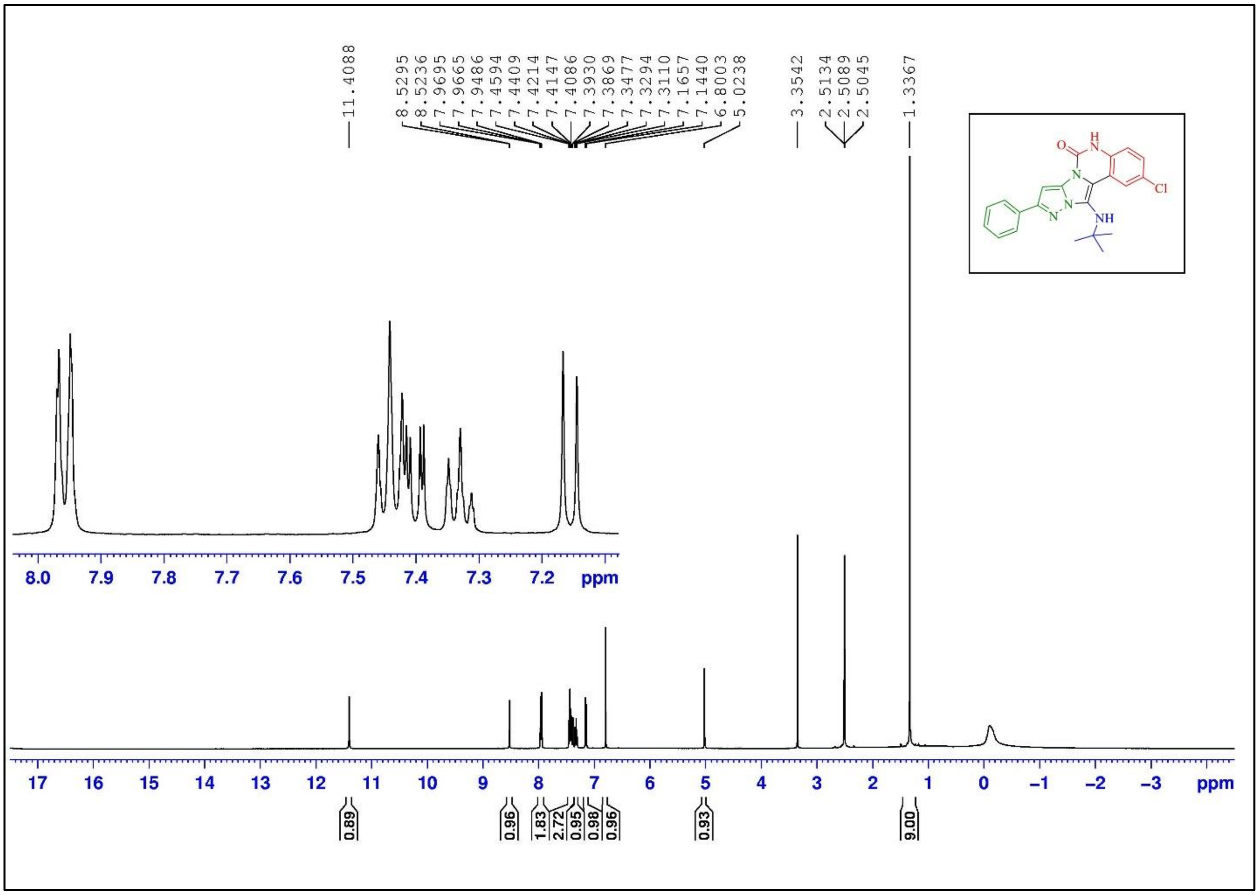

Figure S47. ^{1}H NMR spectrum of compound (4l) in DMSO.

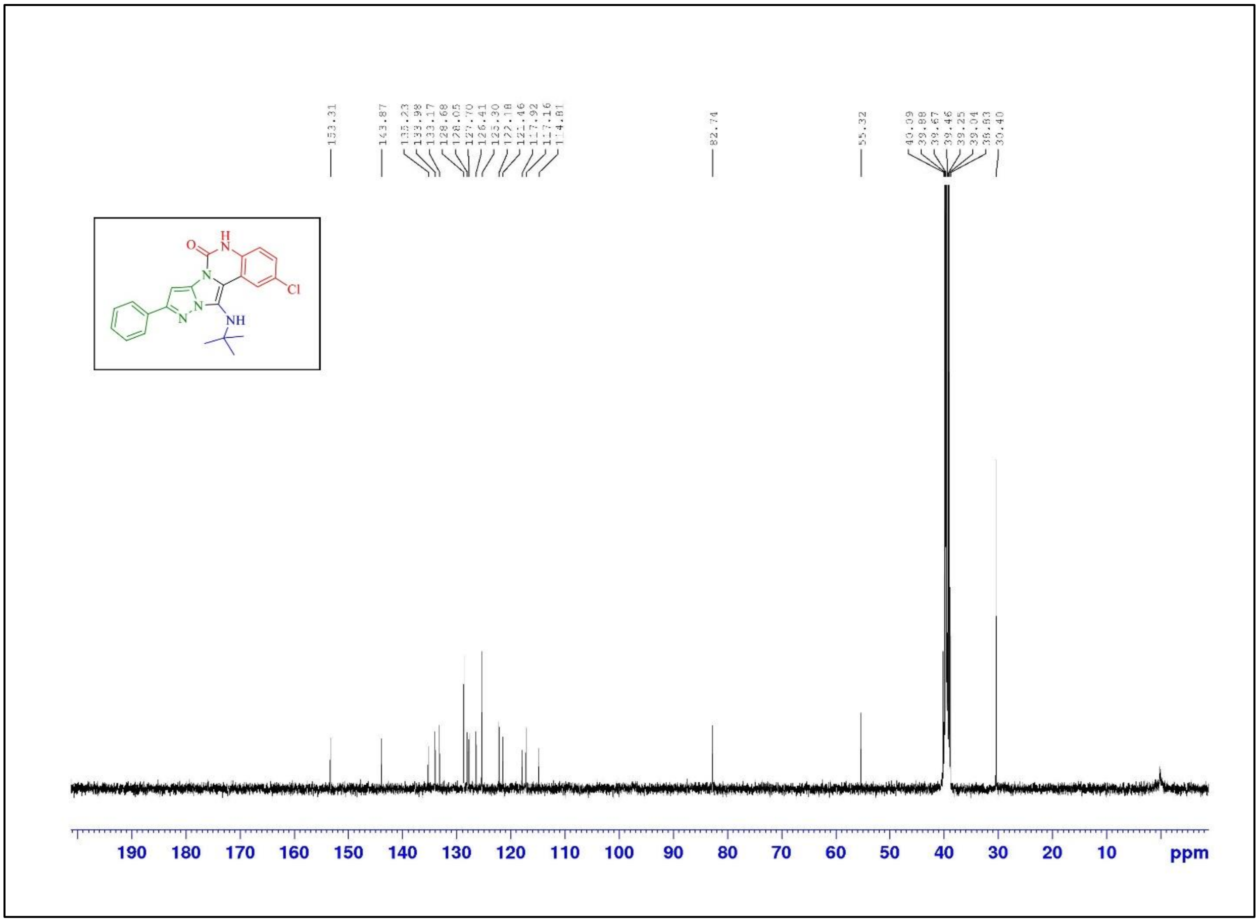

Figure S48. ^{13}C NMR spectrum of compound (4l) in DMSO.

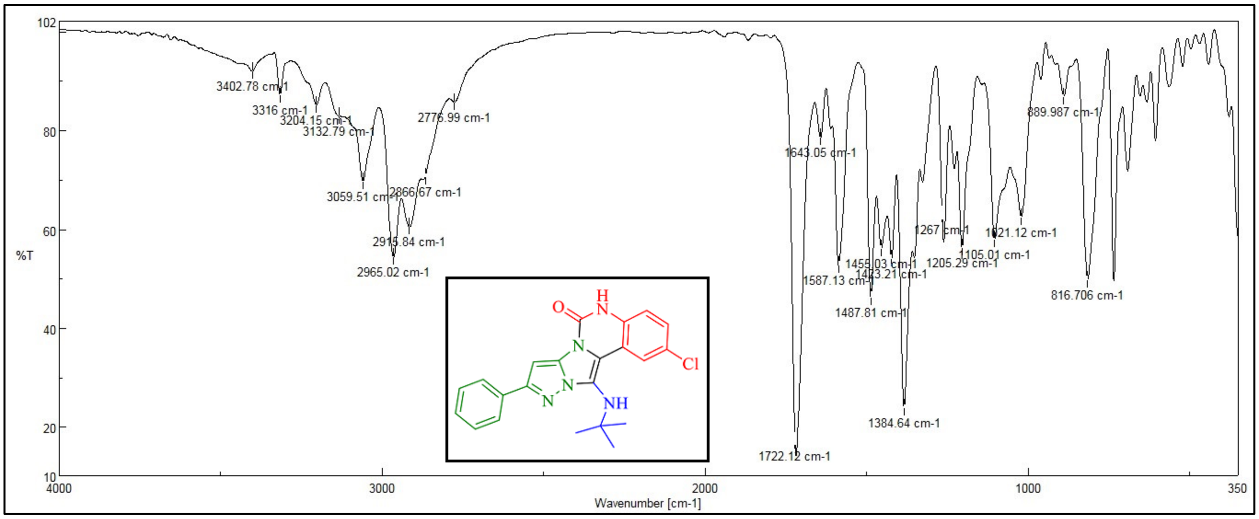

Figure S49. FT-IR spectrum of compound (4l).

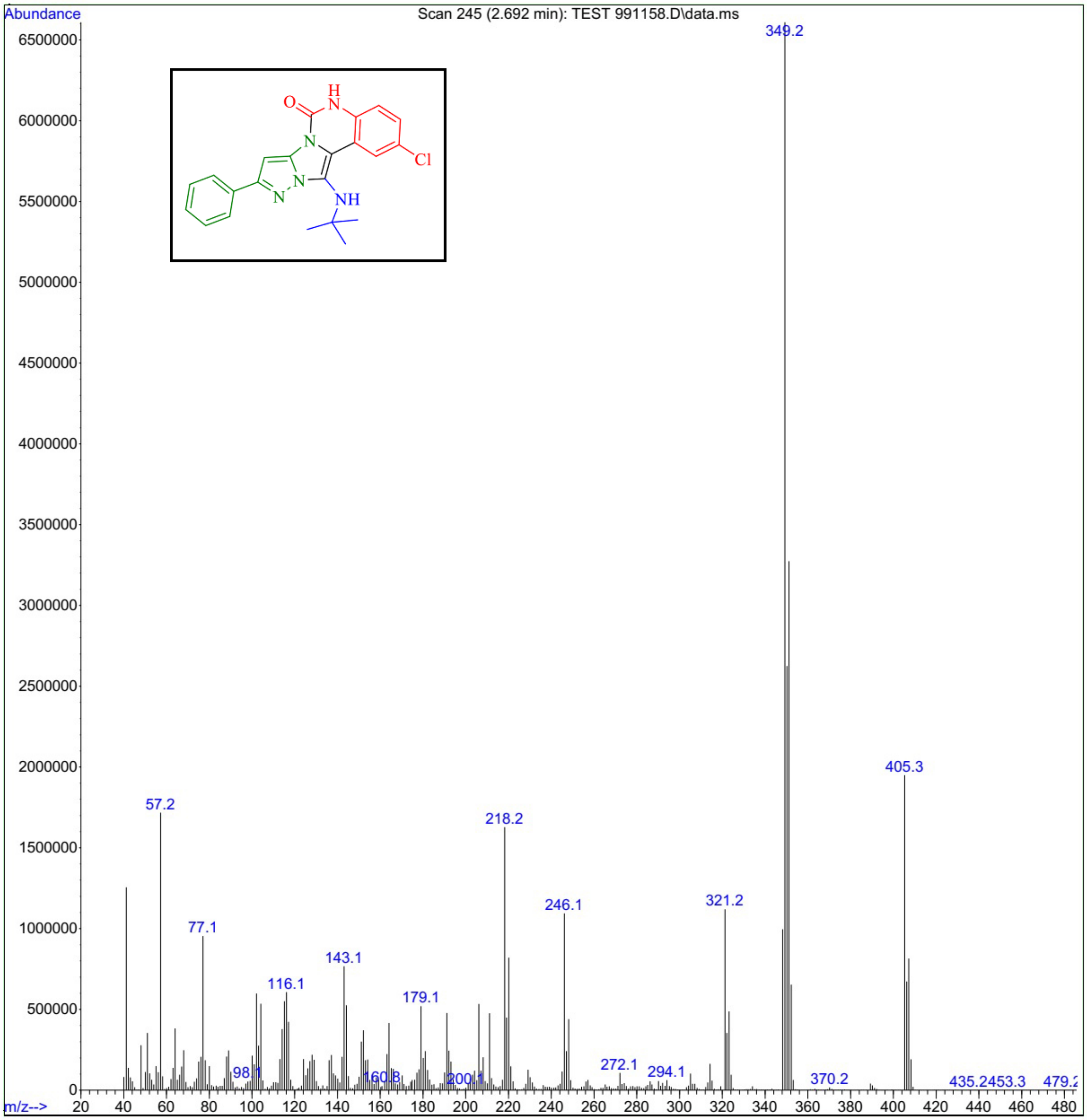

Figure S50. MASS spectrum of compound (4l).

^{1}H NMR, ^{13}C NMR, FT-IR and MASS of compound (4m)

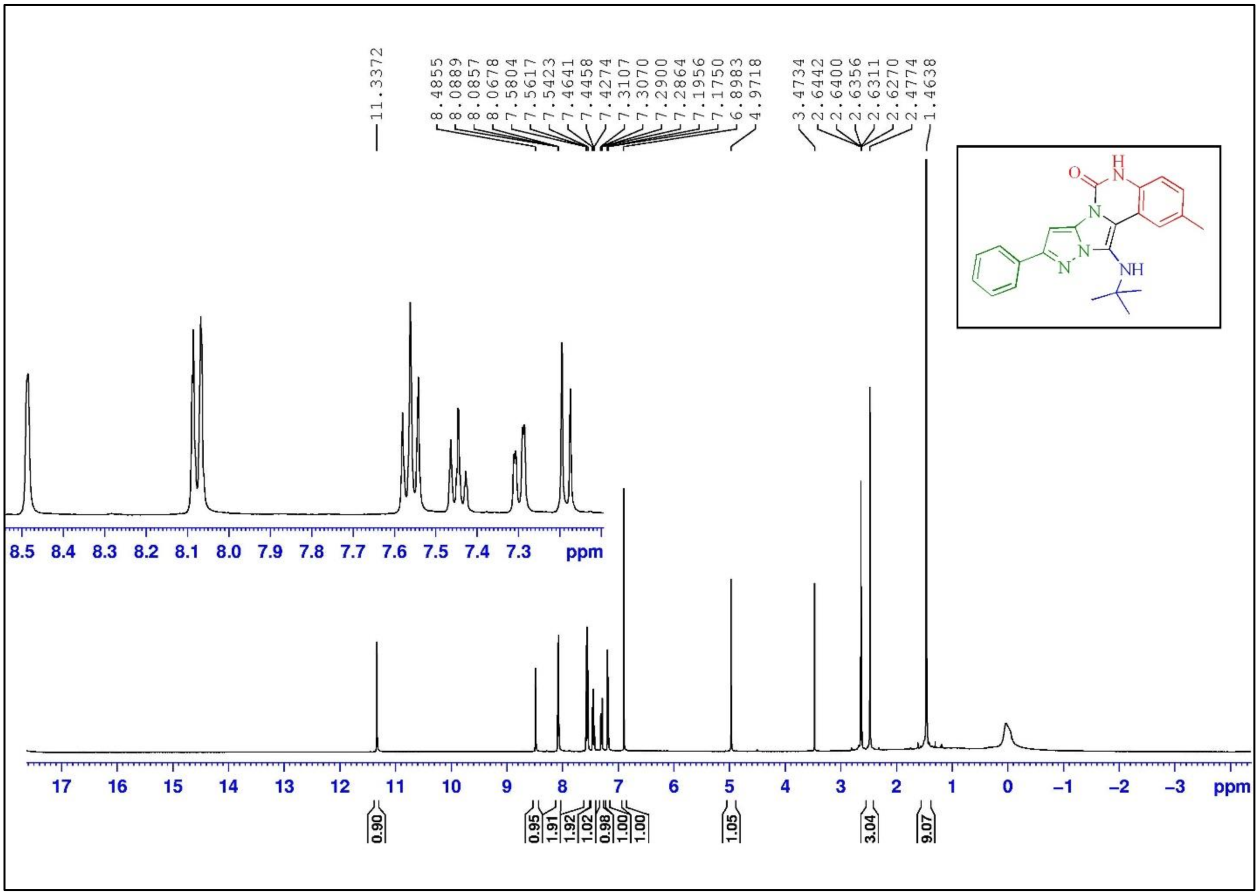

Figure S51. ^{1}H NMR spectrum of compound (4m) in DMSO.

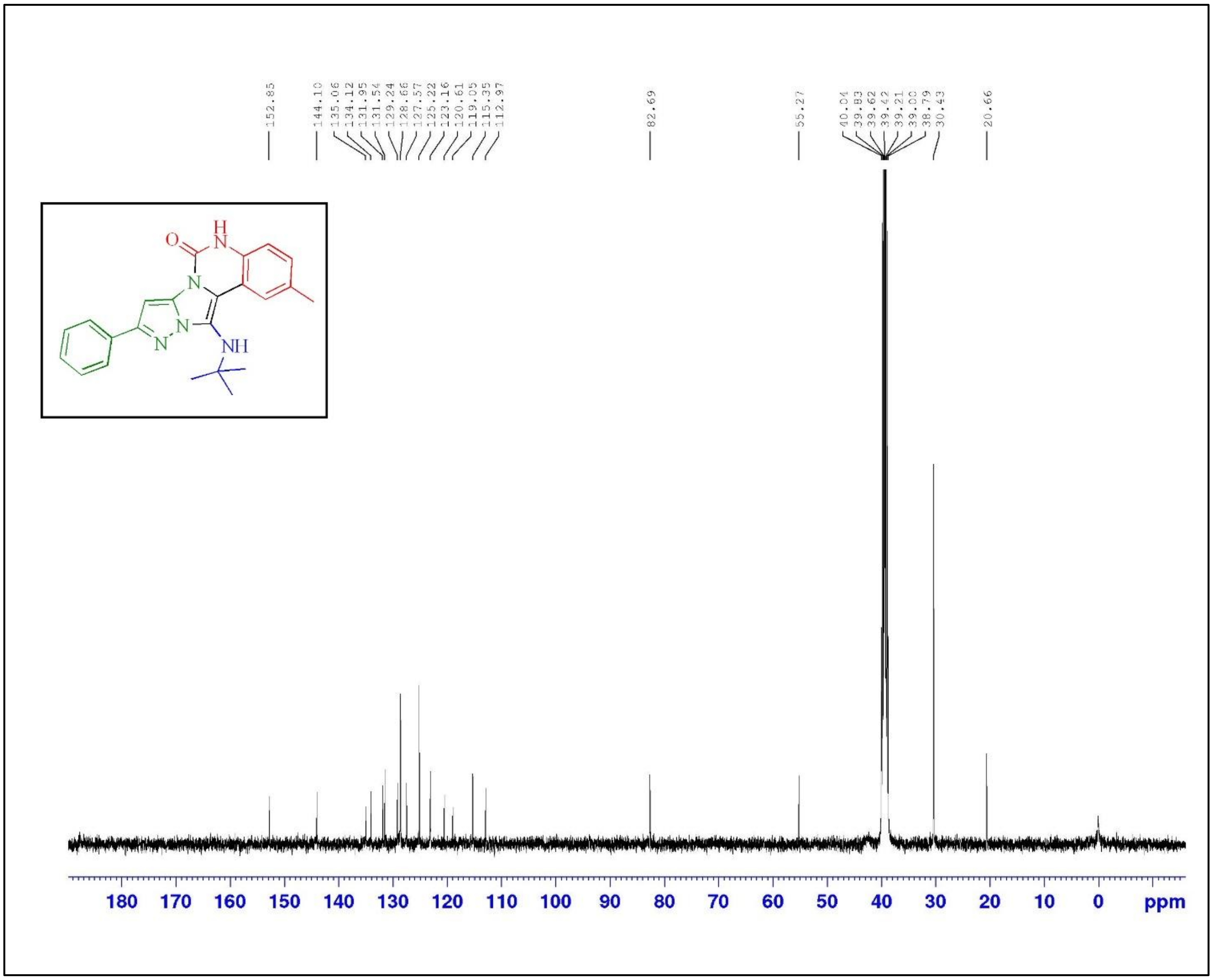

Figure S52. ^{13}C NMR spectrum of compound (4m) in DMSO.

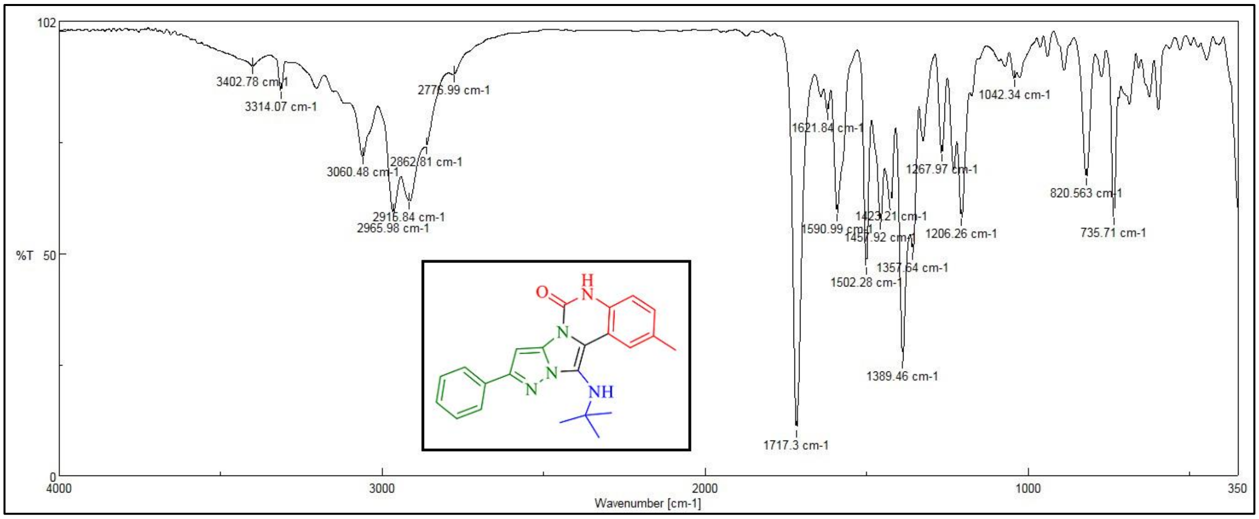

Figure S53. FT-IR spectrum of compound (4m).

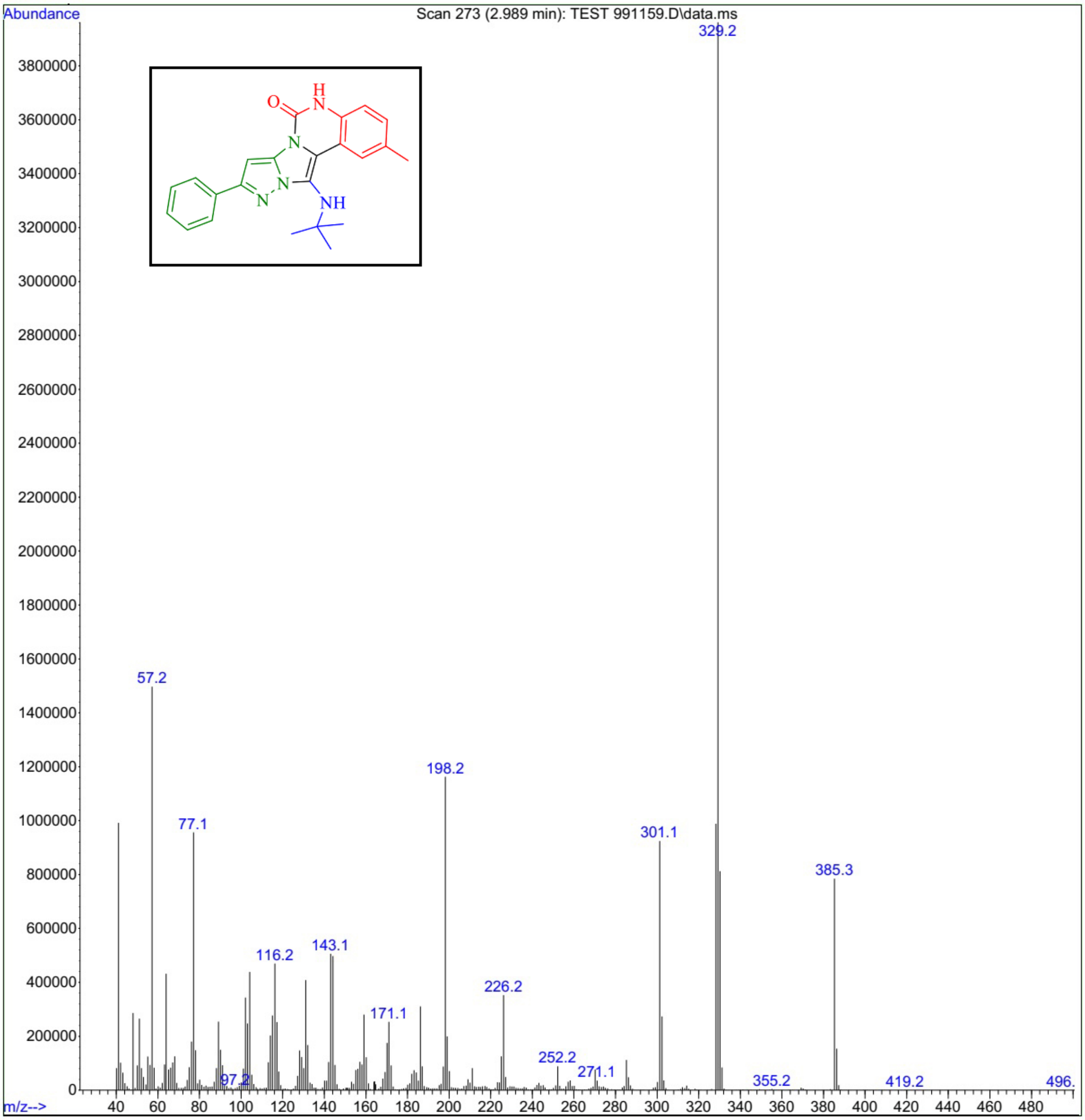

Figure S54. MASS spectrum of compound (4m).

^{1}H NMR, ^{13}C NMR, FT-IR and MASS of compound (4n)

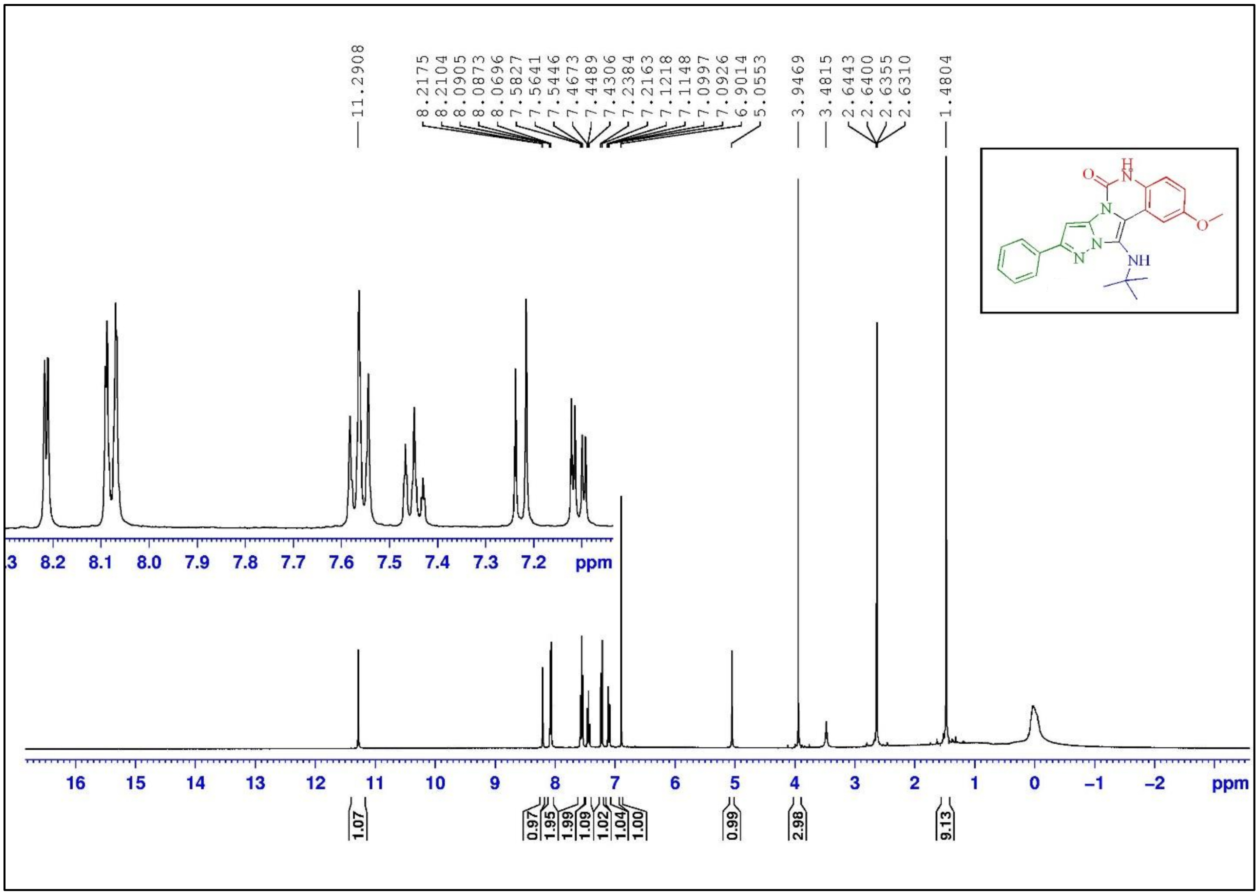

Figure S55. ^{1}H NMR spectrum of compound (4n) in DMSO.

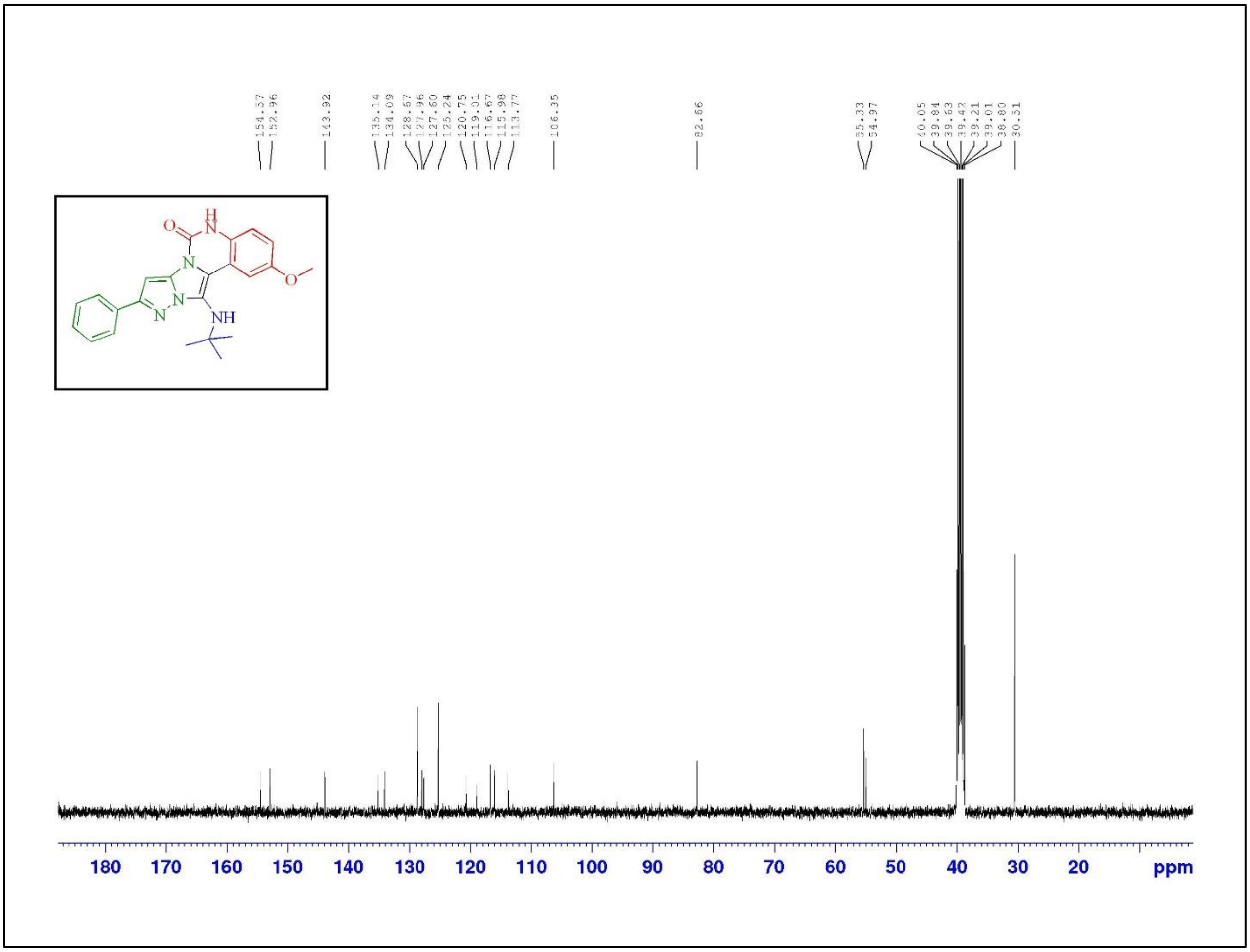

Figure S56. ^{13}C NMR spectrum of compound (4n) in DMSO.

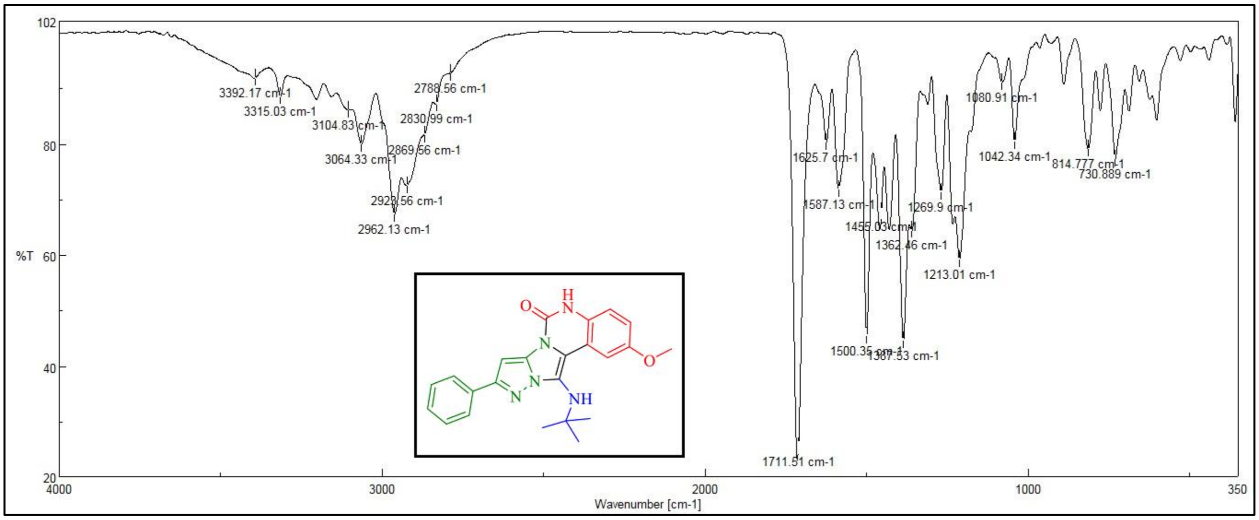

Figure S57. FT-IR spectrum of compound (4n).

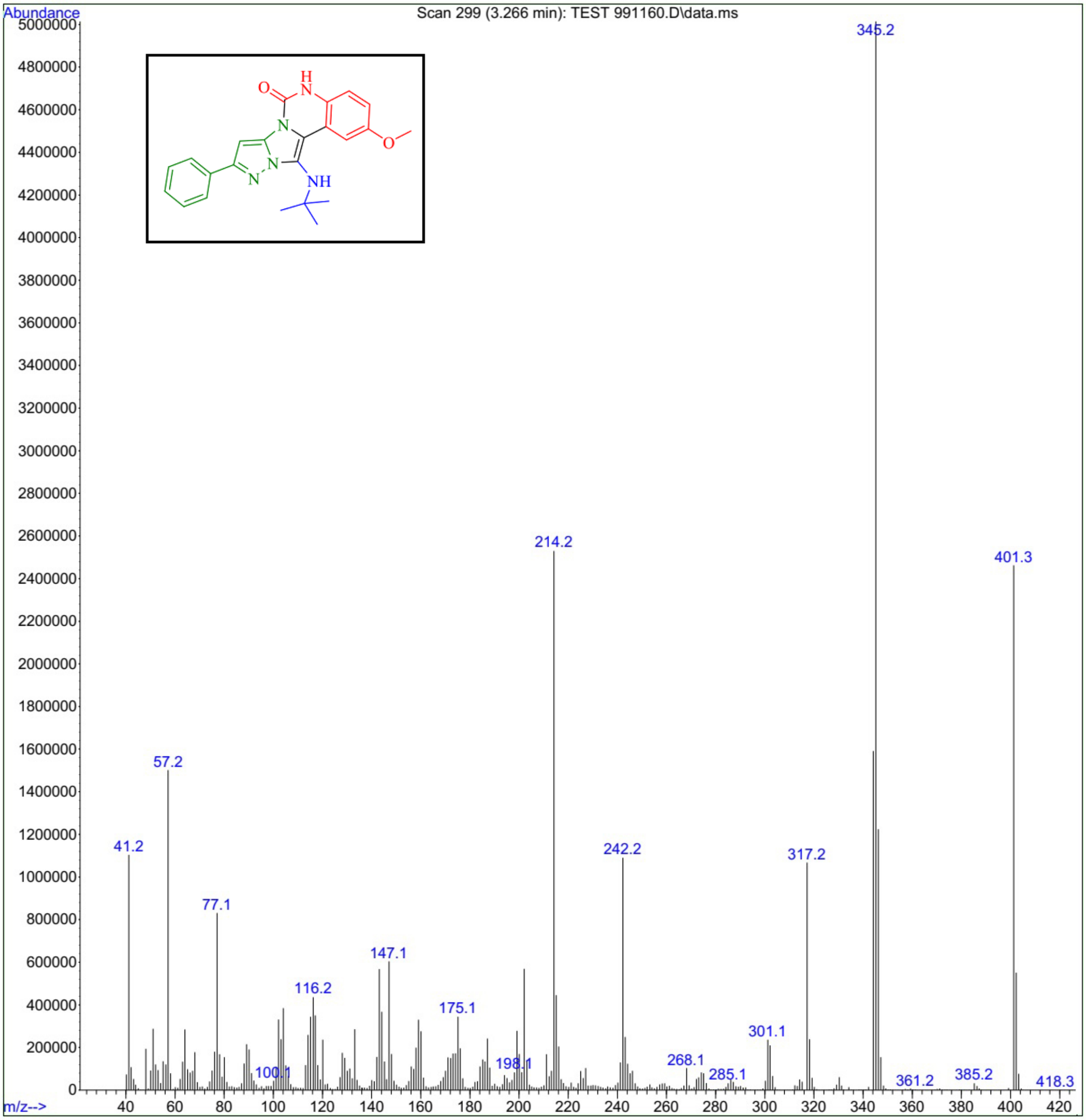

Figure S58. MASS spectrum of compound (4n).

^{1}H NMR, ^{13}C NMR, FT-IR and MASS of compound (4o)

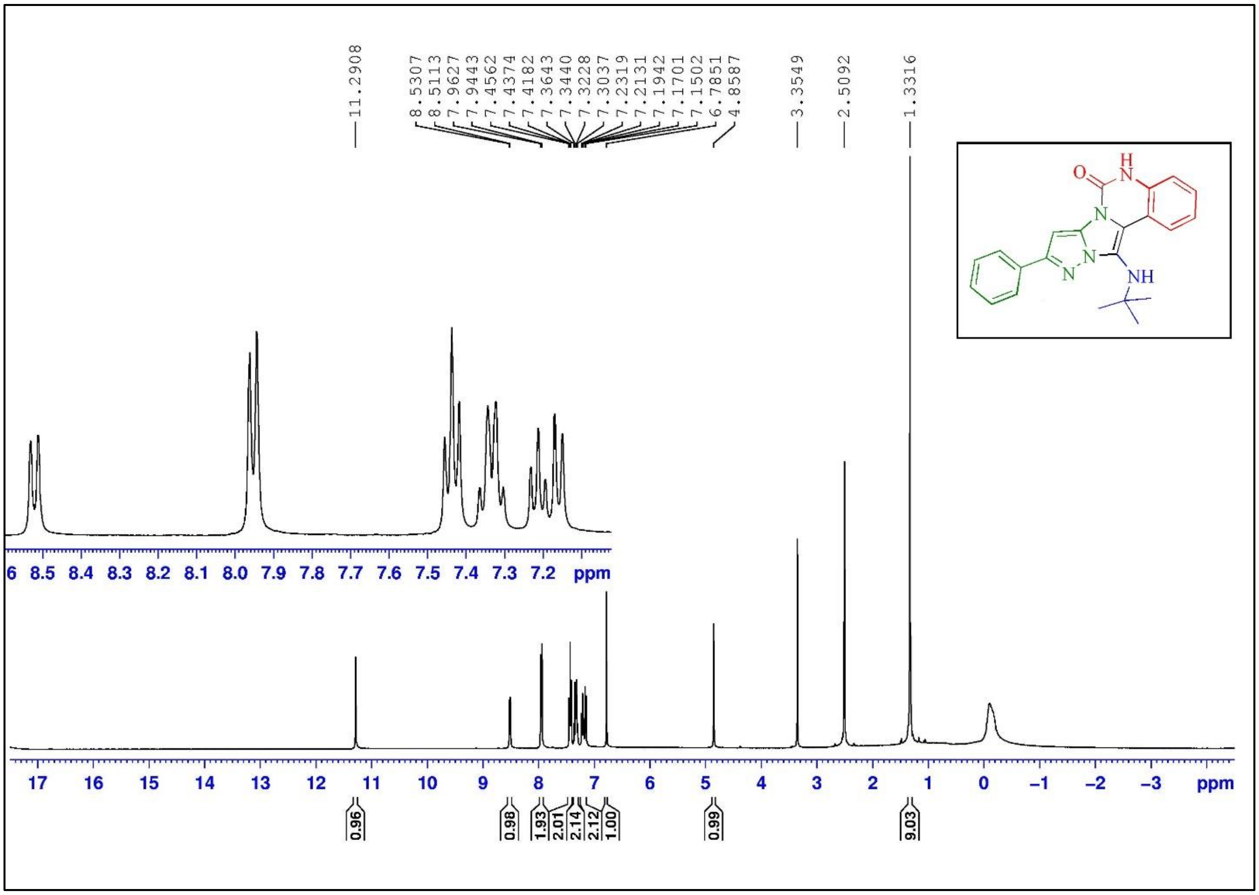

Figure S59. ^{1}H NMR spectrum of compound (4o) in DMSO.

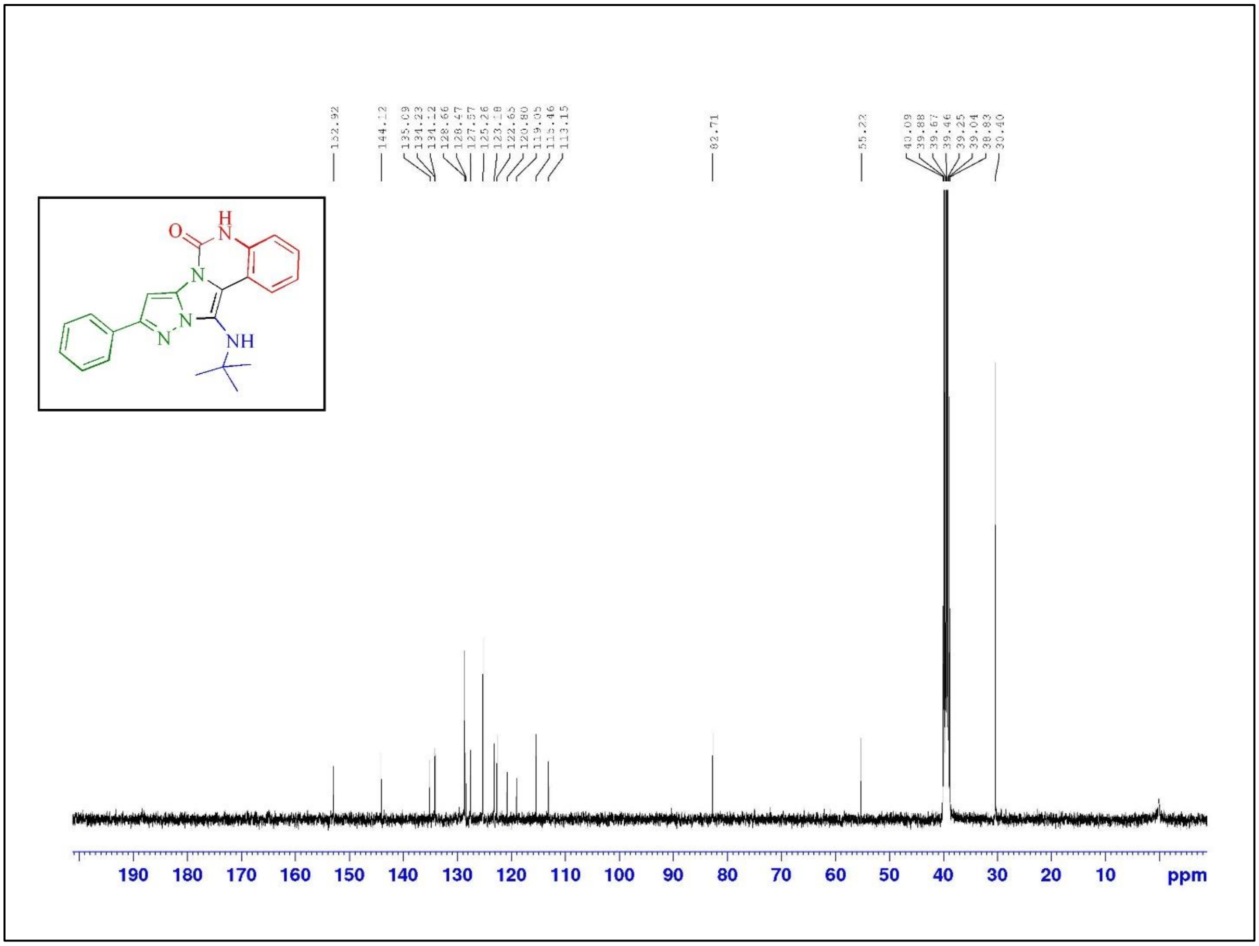

Figure S60. ^{13}C NMR spectrum of compound (4o) in DMSO.

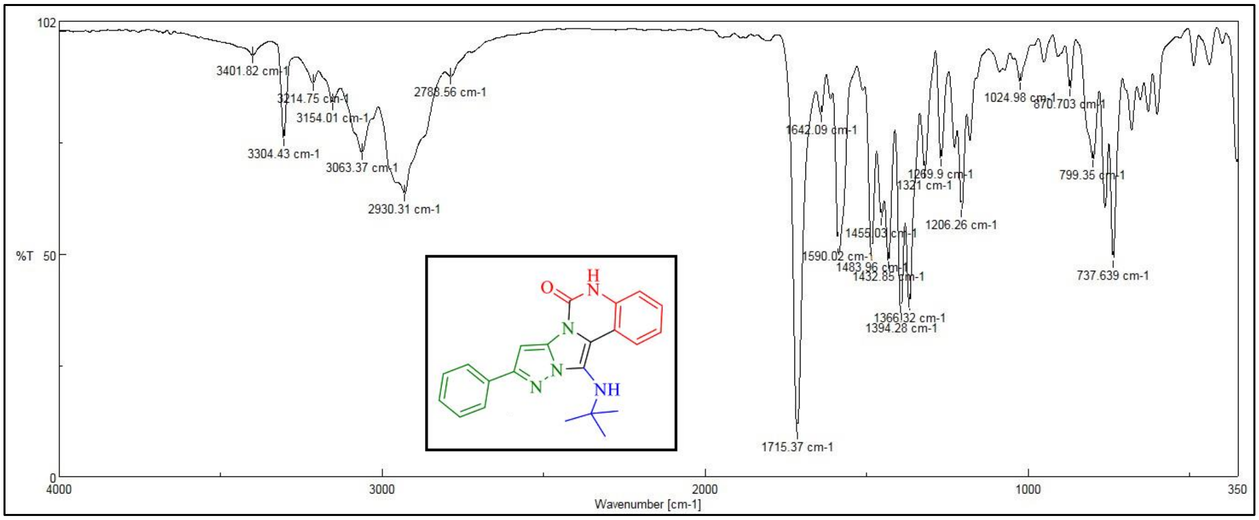

Figure S61. FT-IR spectrum of compound (4o).

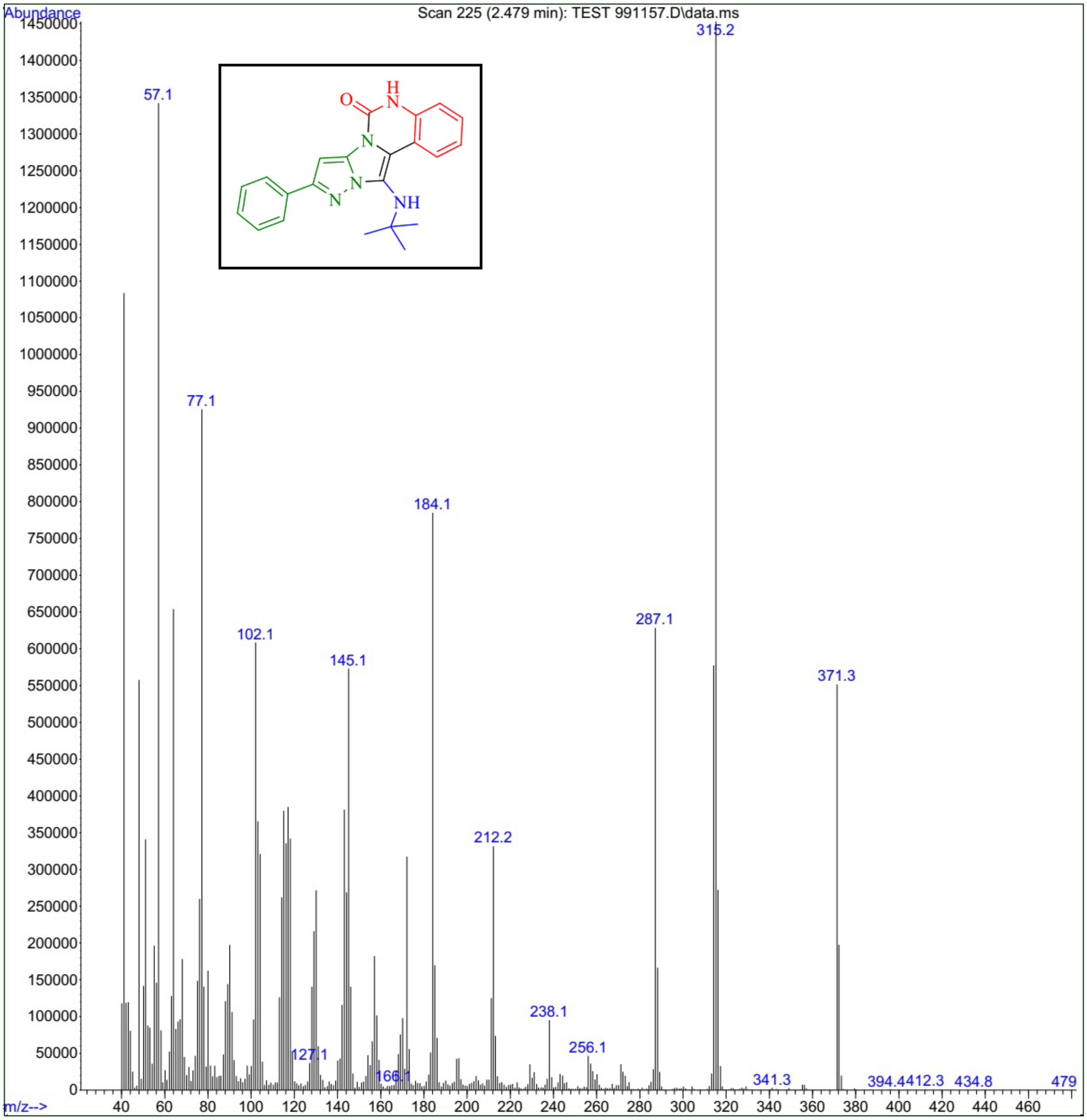

Figure S62. MASS spectrum of compound (4o).

^{1}H NMR, ^{13}C NMR, FT-IR and MASS of compound (p)

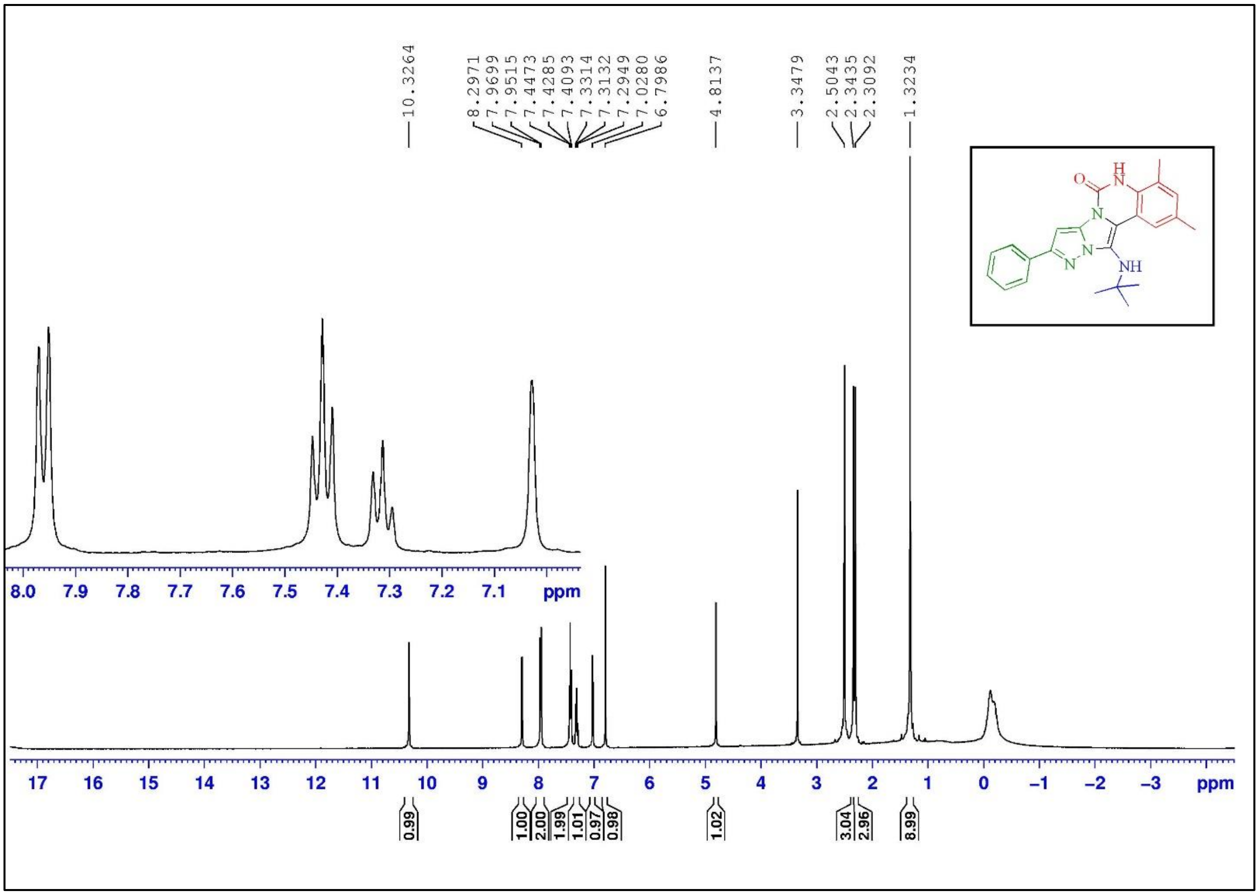

Figure S63. ^{1}H NMR spectrum of compound (4p) in DMSO.

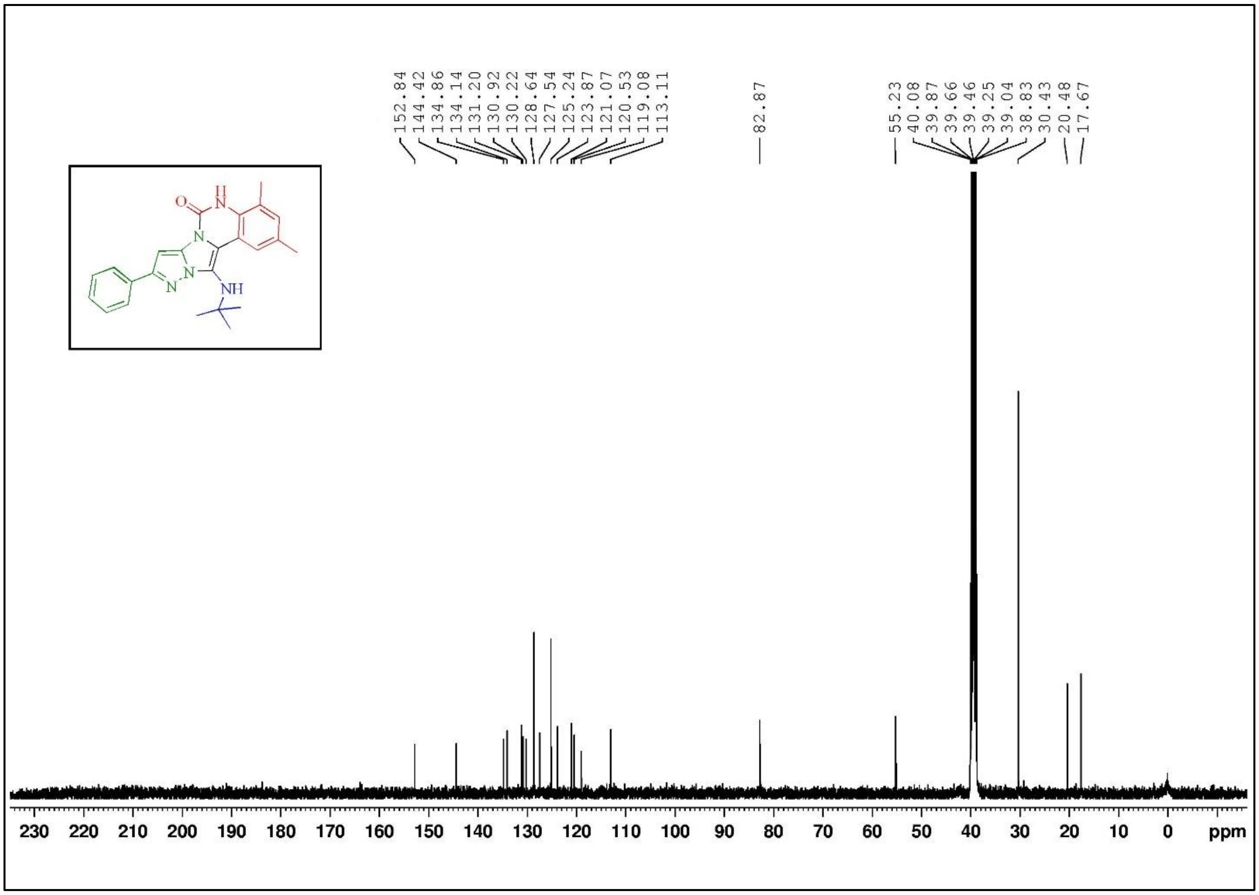

Figure S64. ^{13}C NMR spectrum of compound (4p) in DMSO.

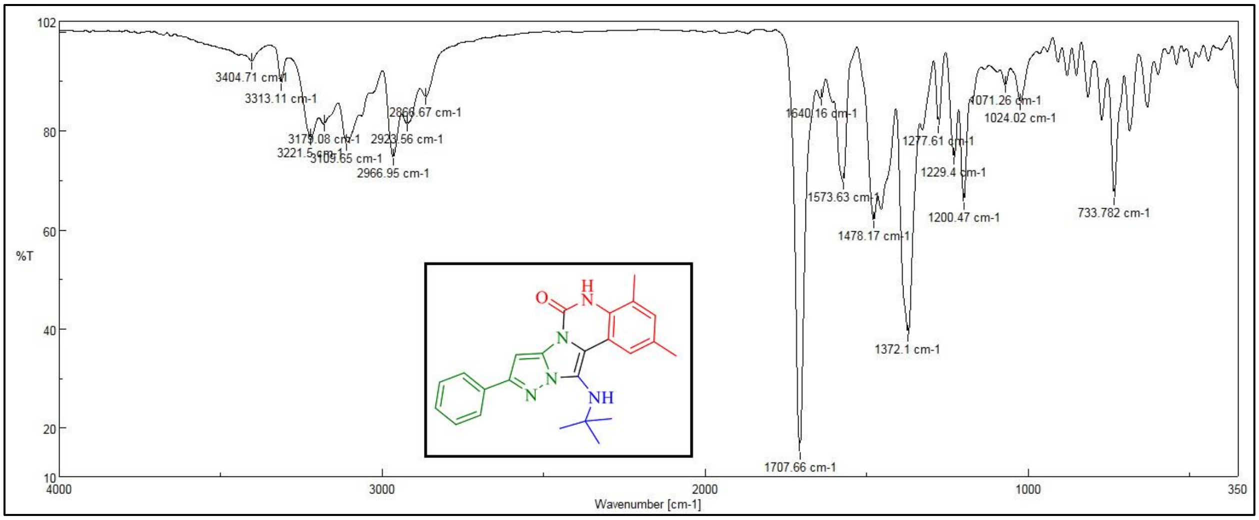

Figure S65. FT-IR spectrum of compound (4p).

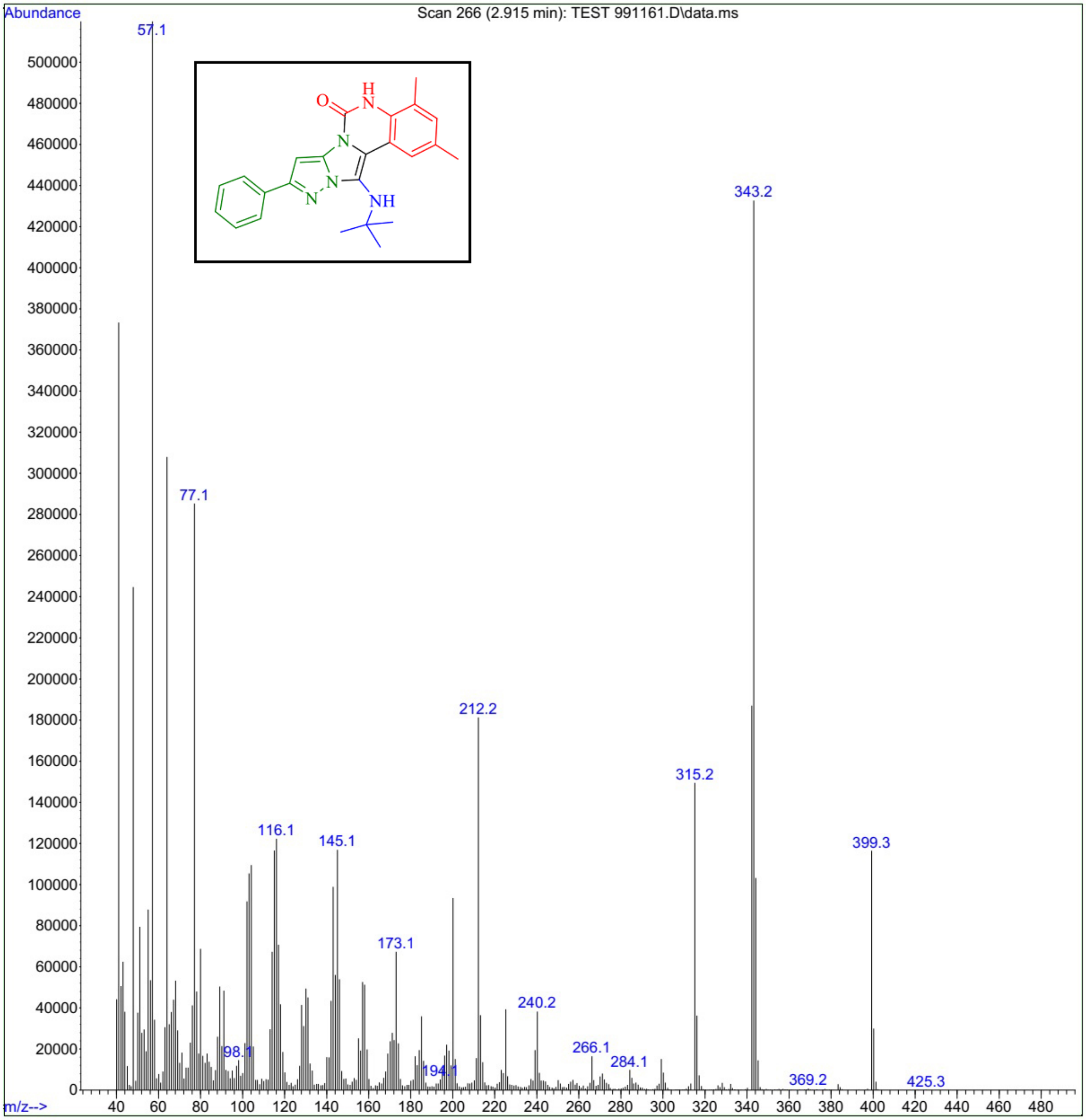

Figure S66. MASS spectrum of compound (4p).

^{1}H NMR, ^{13}C NMR, FT-IR and MASS of compound (4q)

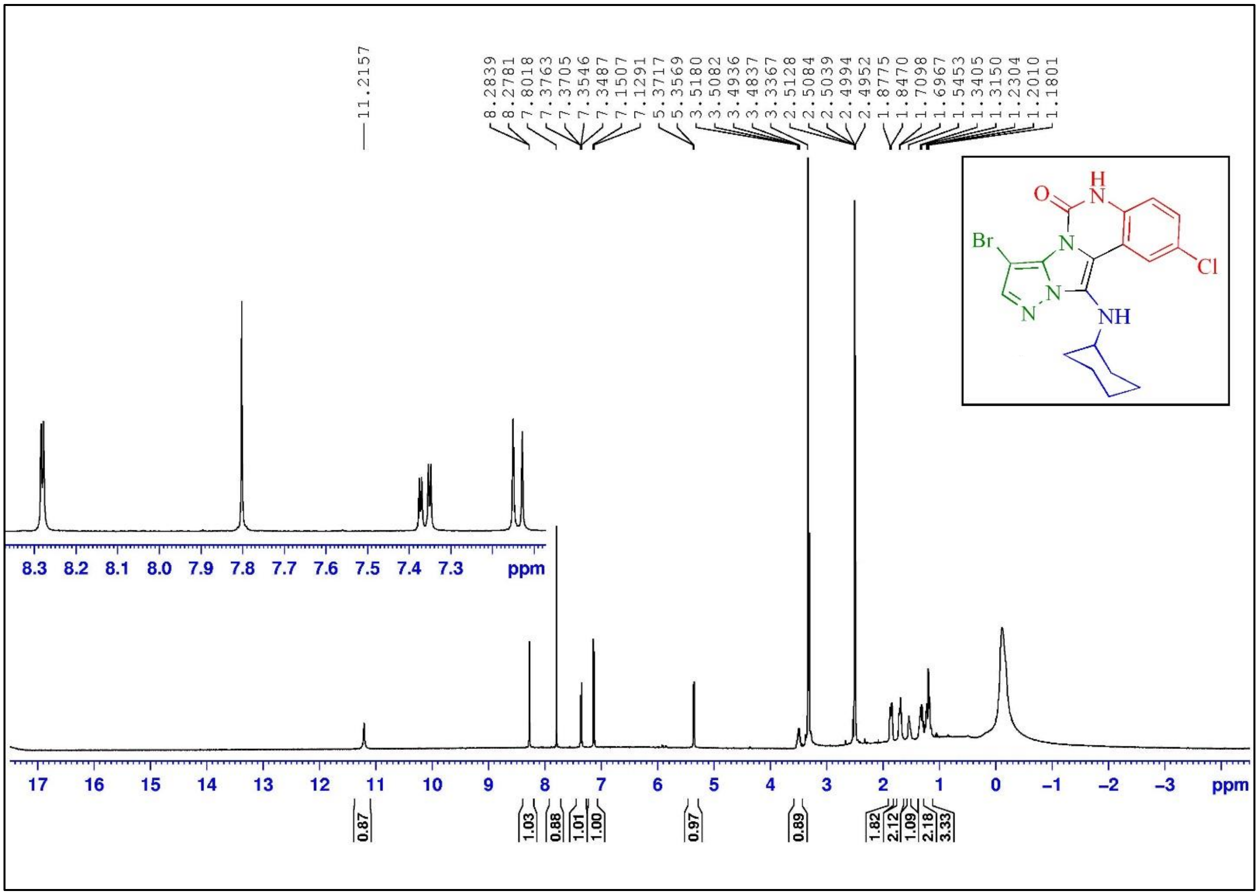

Figure S67. ^{1}H NMR spectrum of compound (4q) in DMSO.

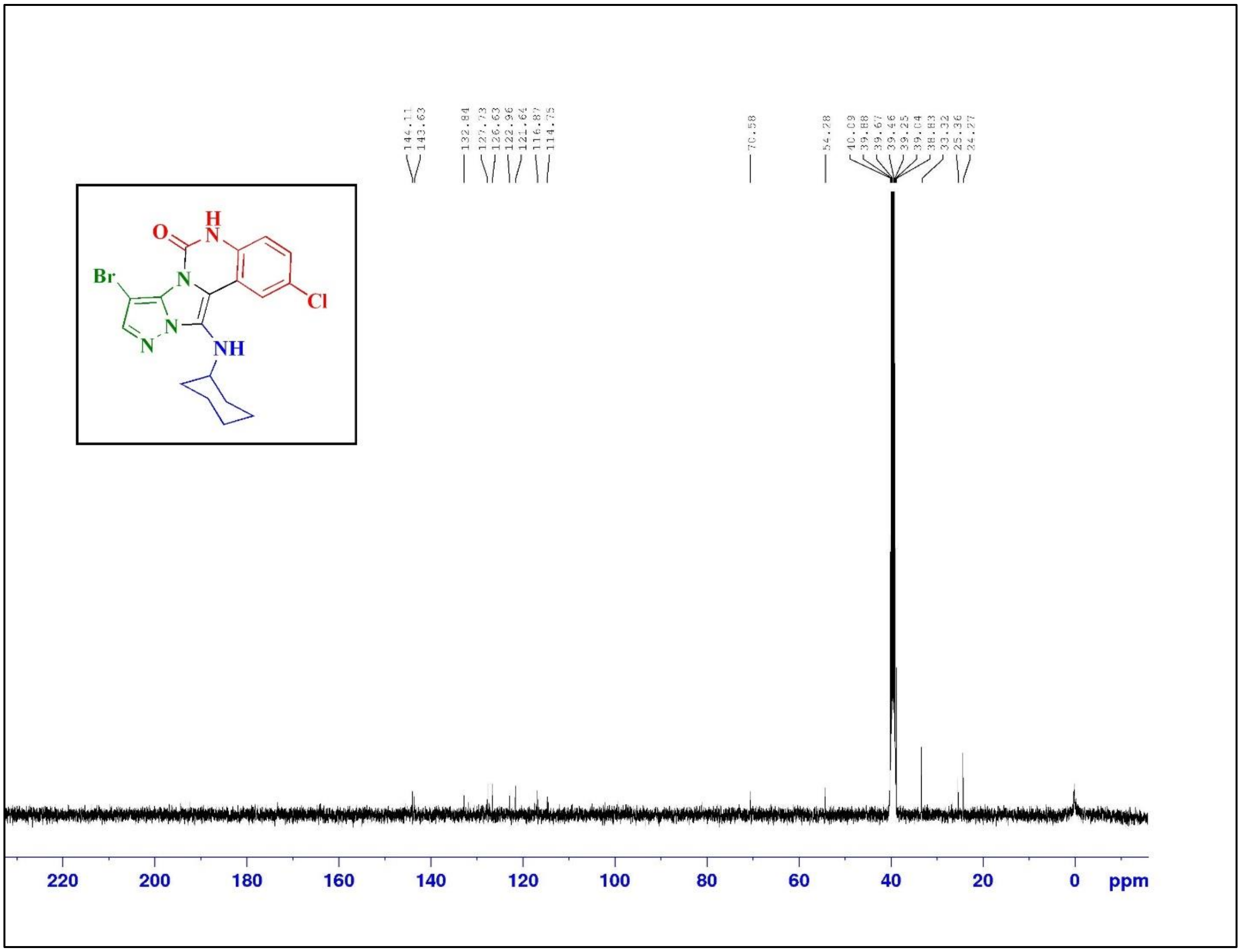

Figure S68. ^{13}C NMR spectrum of compound (4q) in DMSO.

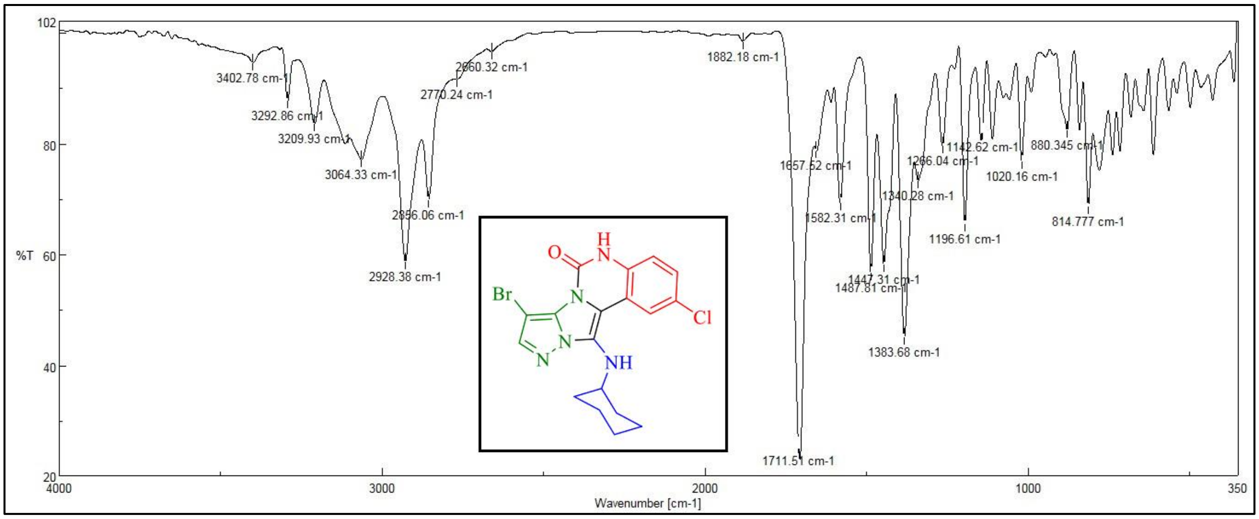

Figure S69. FT-IR spectrum of compound (4q).

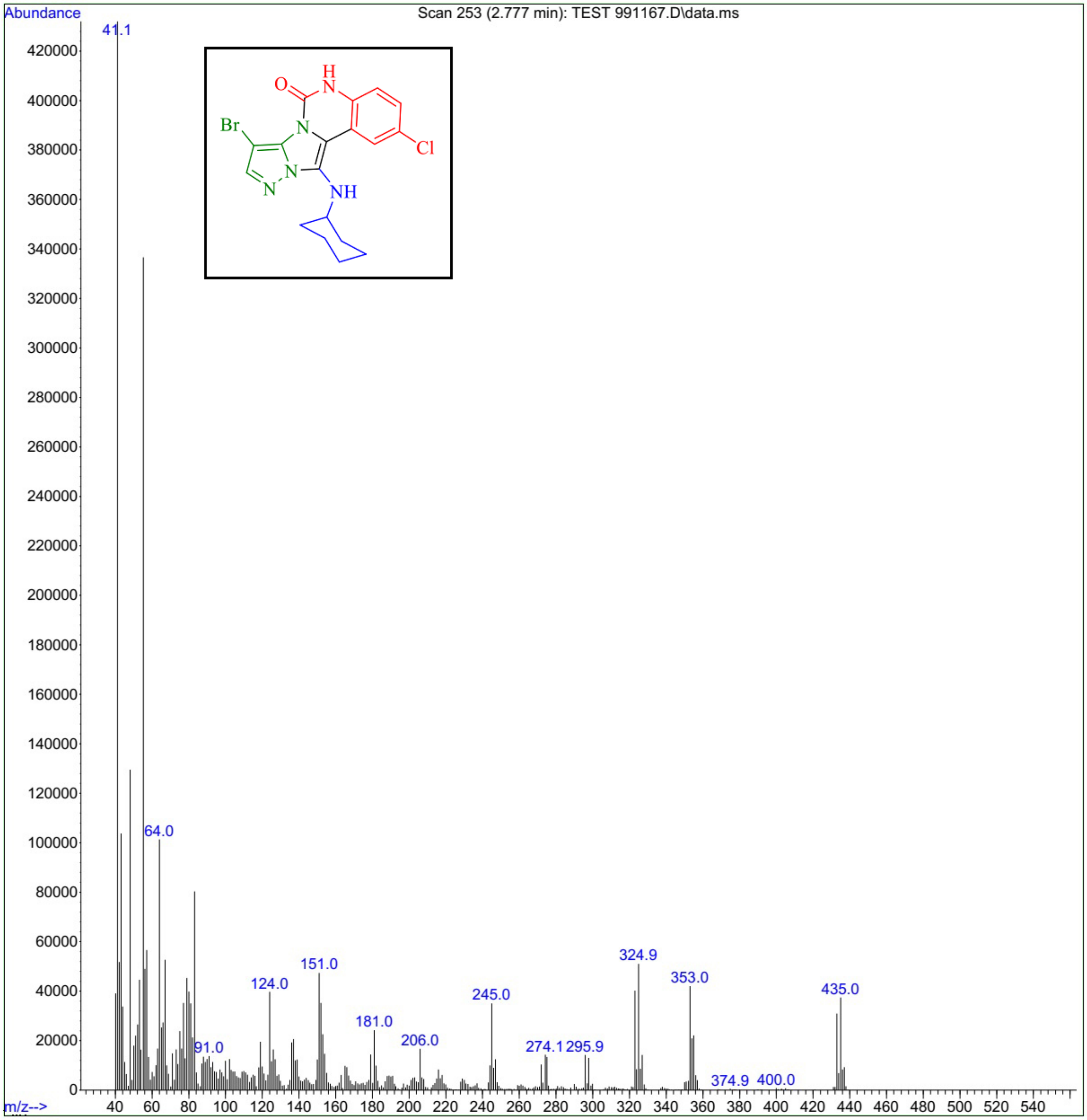

Figure S70. MASS spectrum of compound (4q).

^{1}H NMR, ^{13}C NMR, FT-IR and MASS of compound (4r)

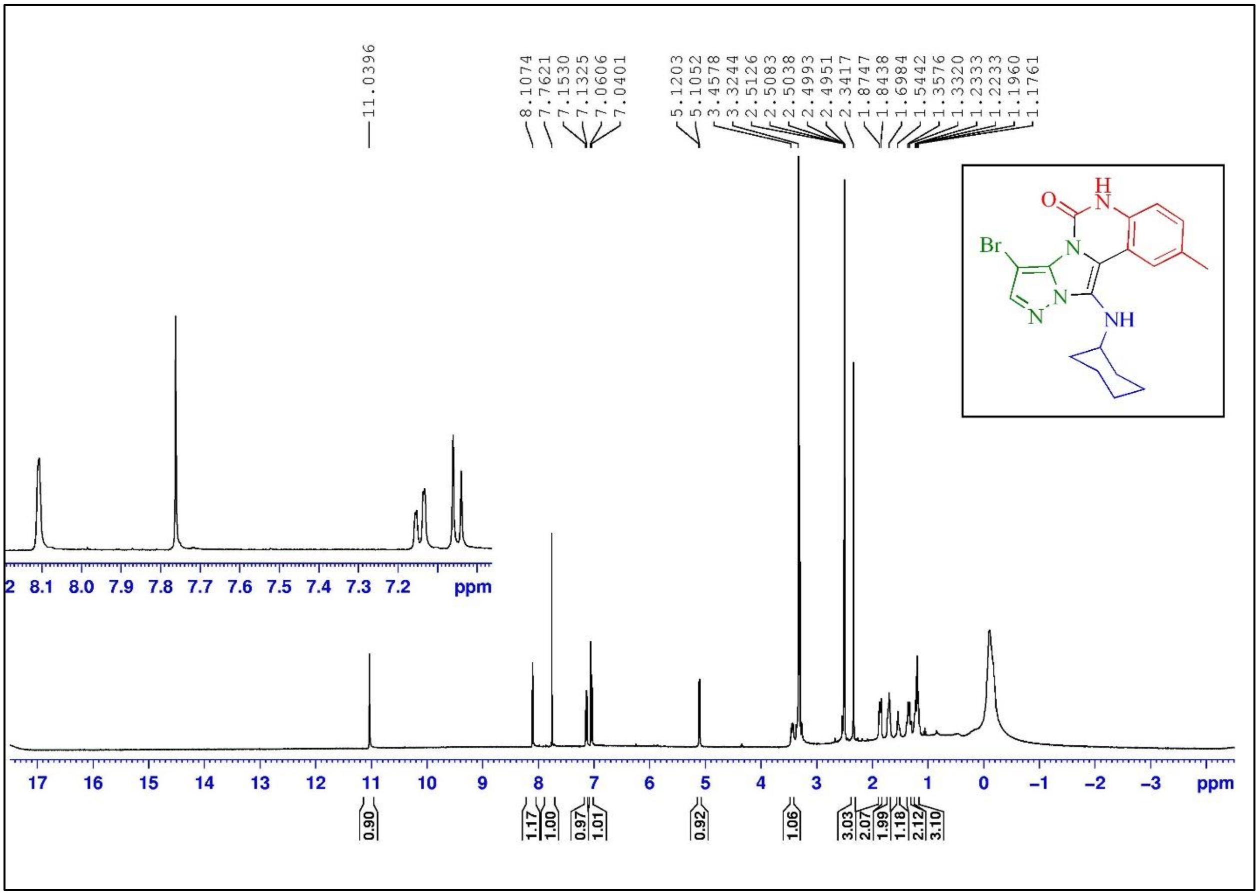

Figure S71. ^{1}H NMR spectrum of compound (4r) in DMSO.

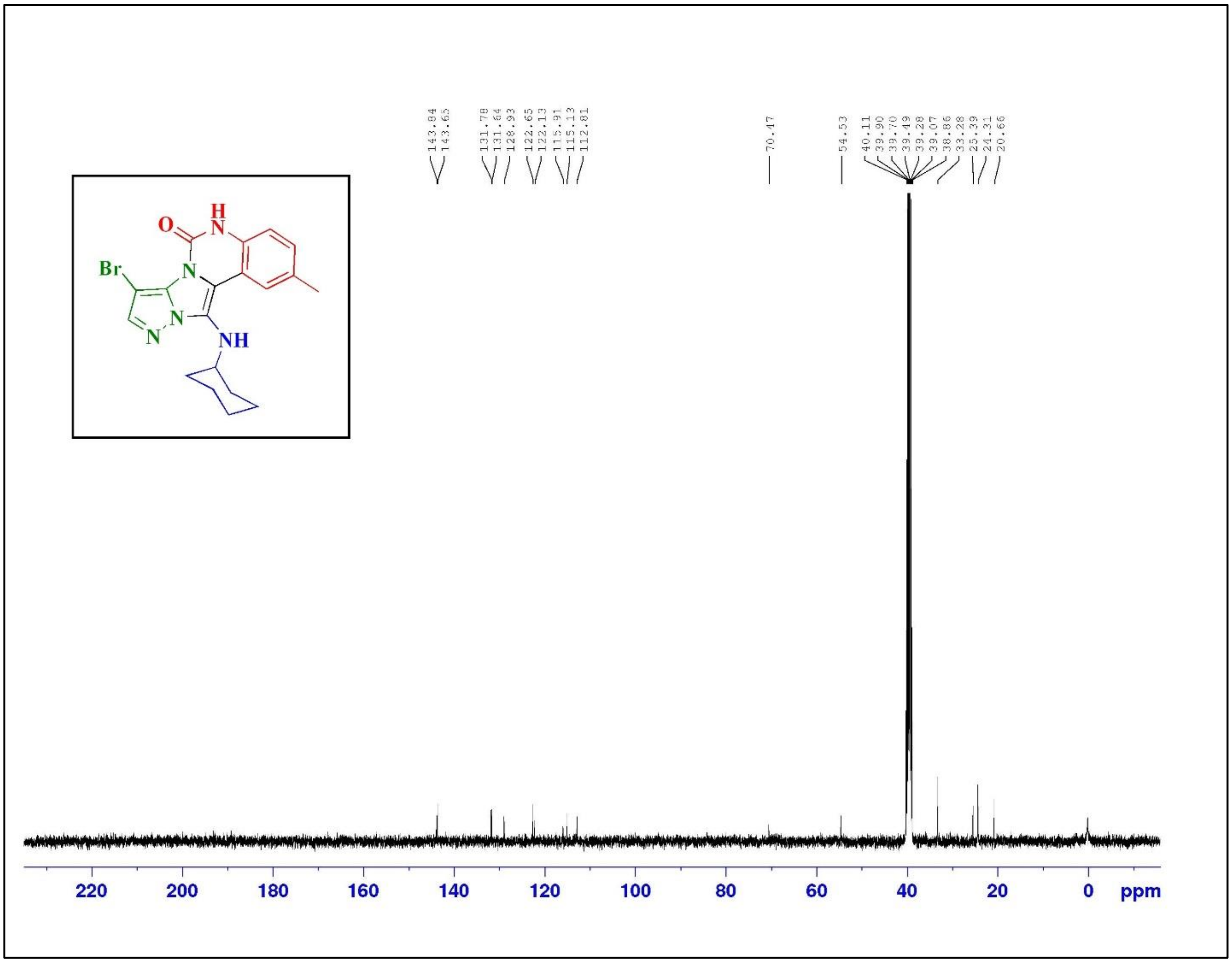

Figure S72. ^{13}C NMR spectrum of compound (4r) in DMSO.

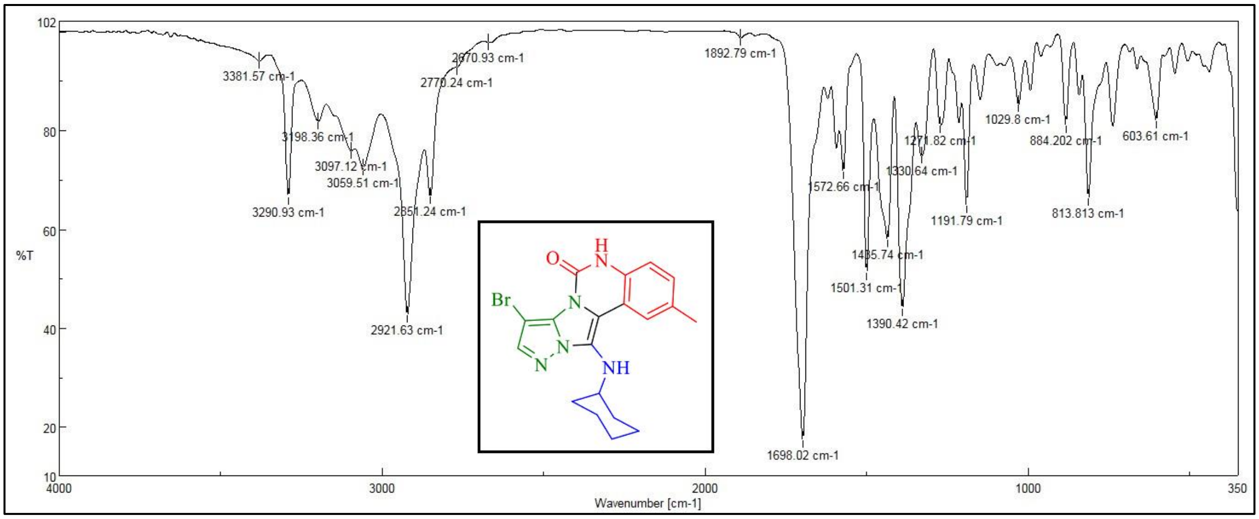

Figure S73. FT-IR spectrum of compound (4r).

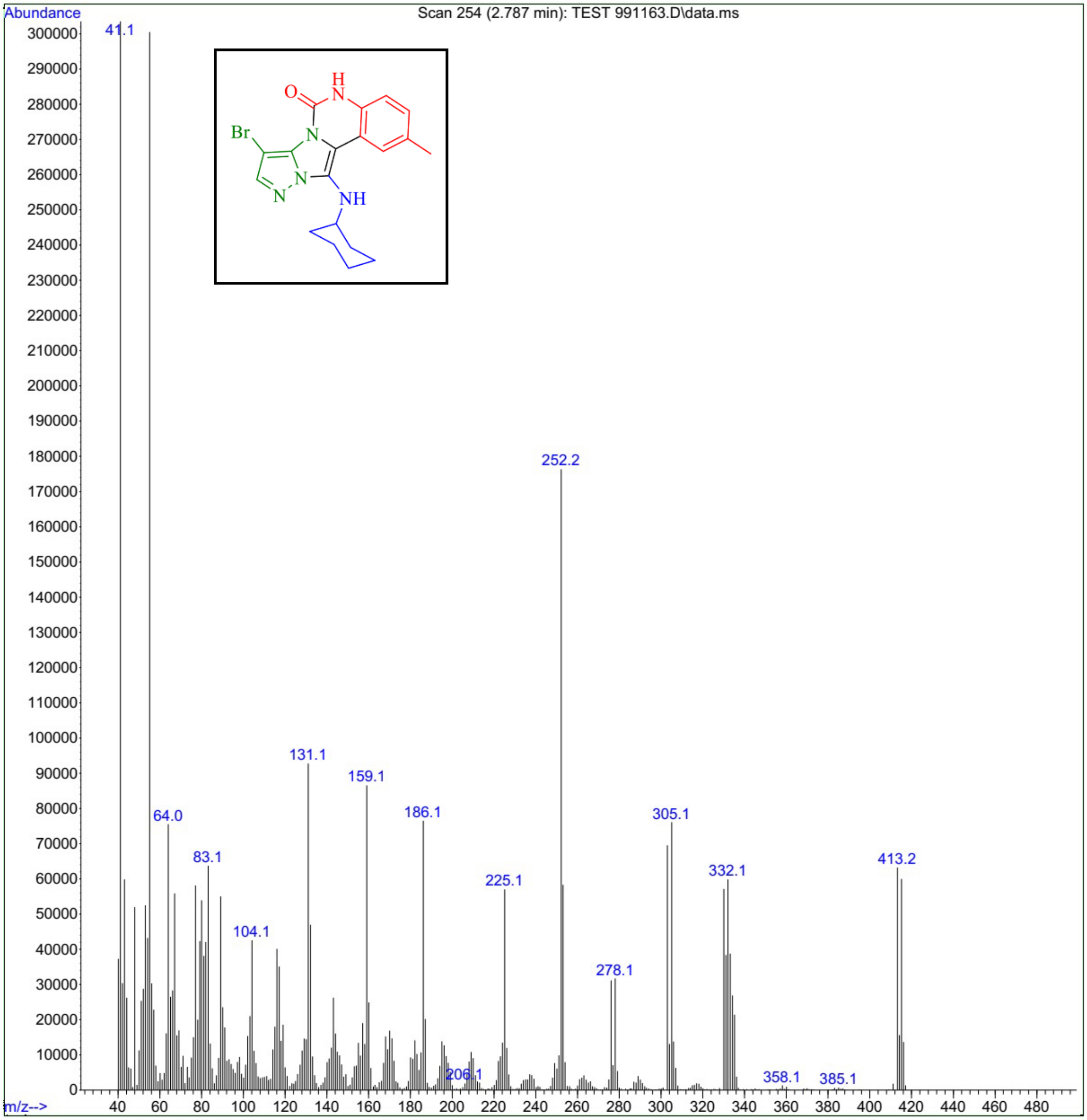

Figure S74. MASS spectrum of compound (4r).

^{1}H NMR, ^{13}C NMR, FT-IR and MASS of compound (4s)

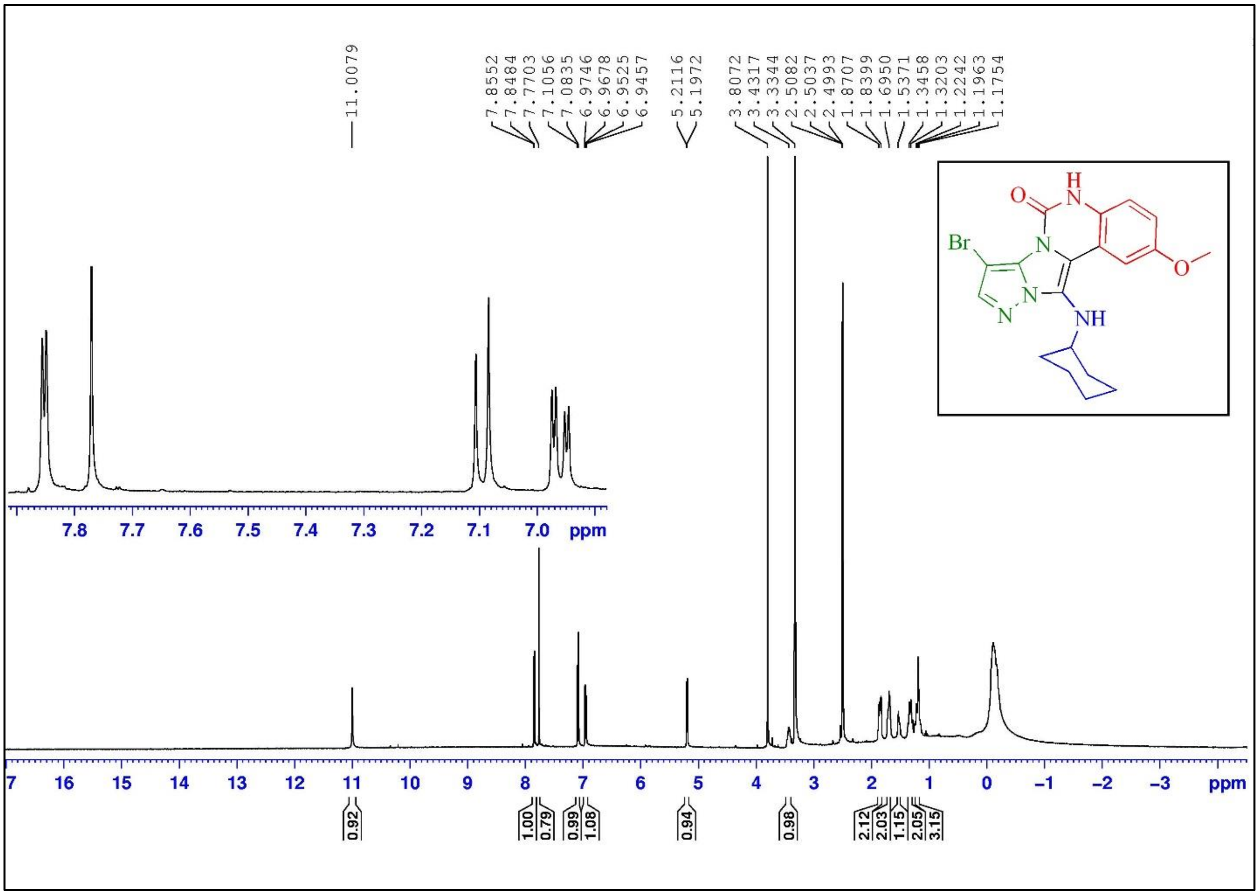

Figure S75. ^{1}H NMR spectrum of compound (4s) in DMSO.

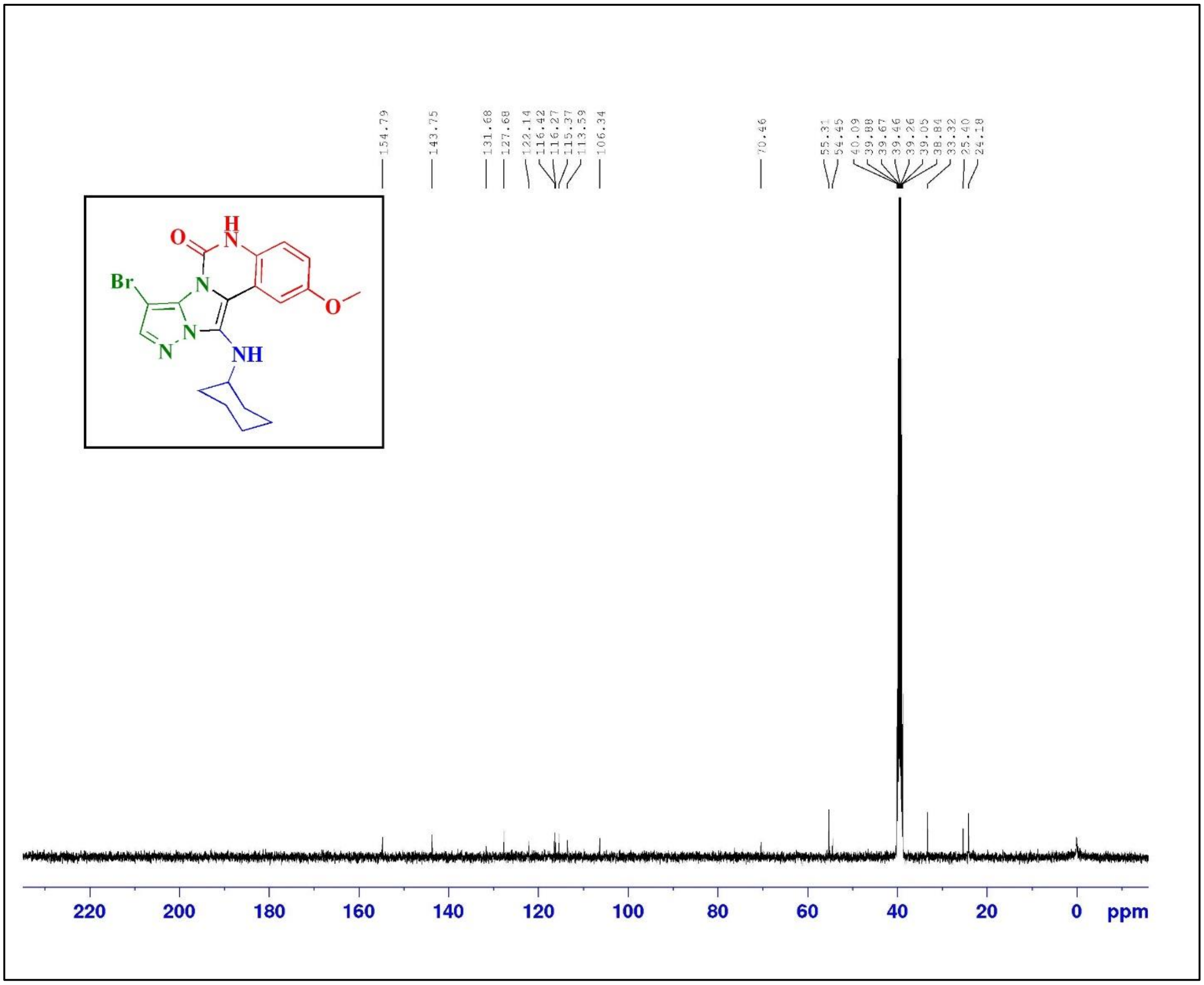

Figure S76. ^{13}C NMR spectrum of compound (4s) in DMSO.

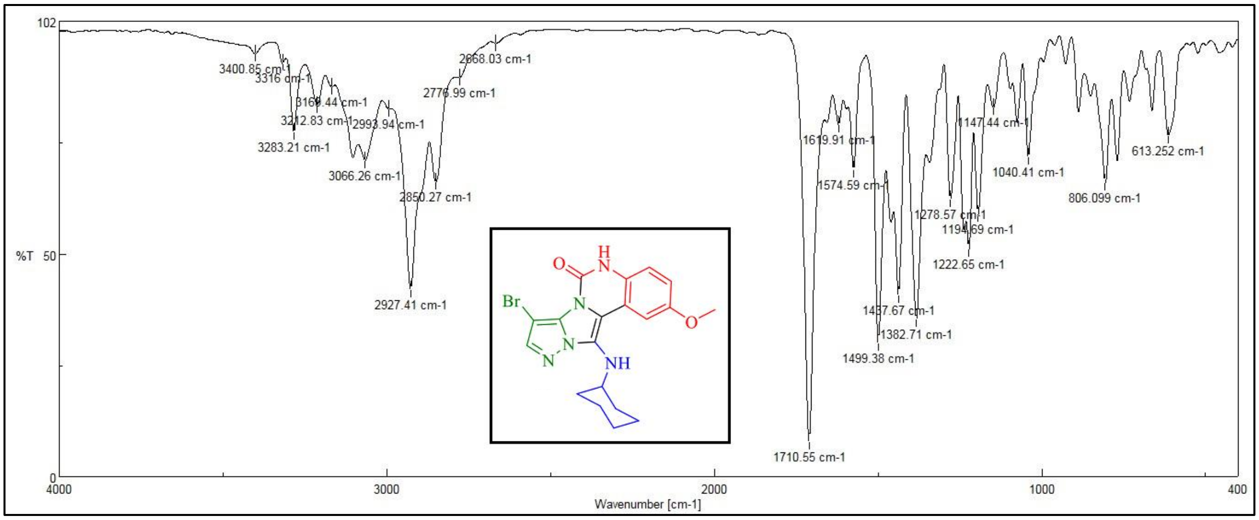

Figure S77. FT-IR spectrum of compound (4s).

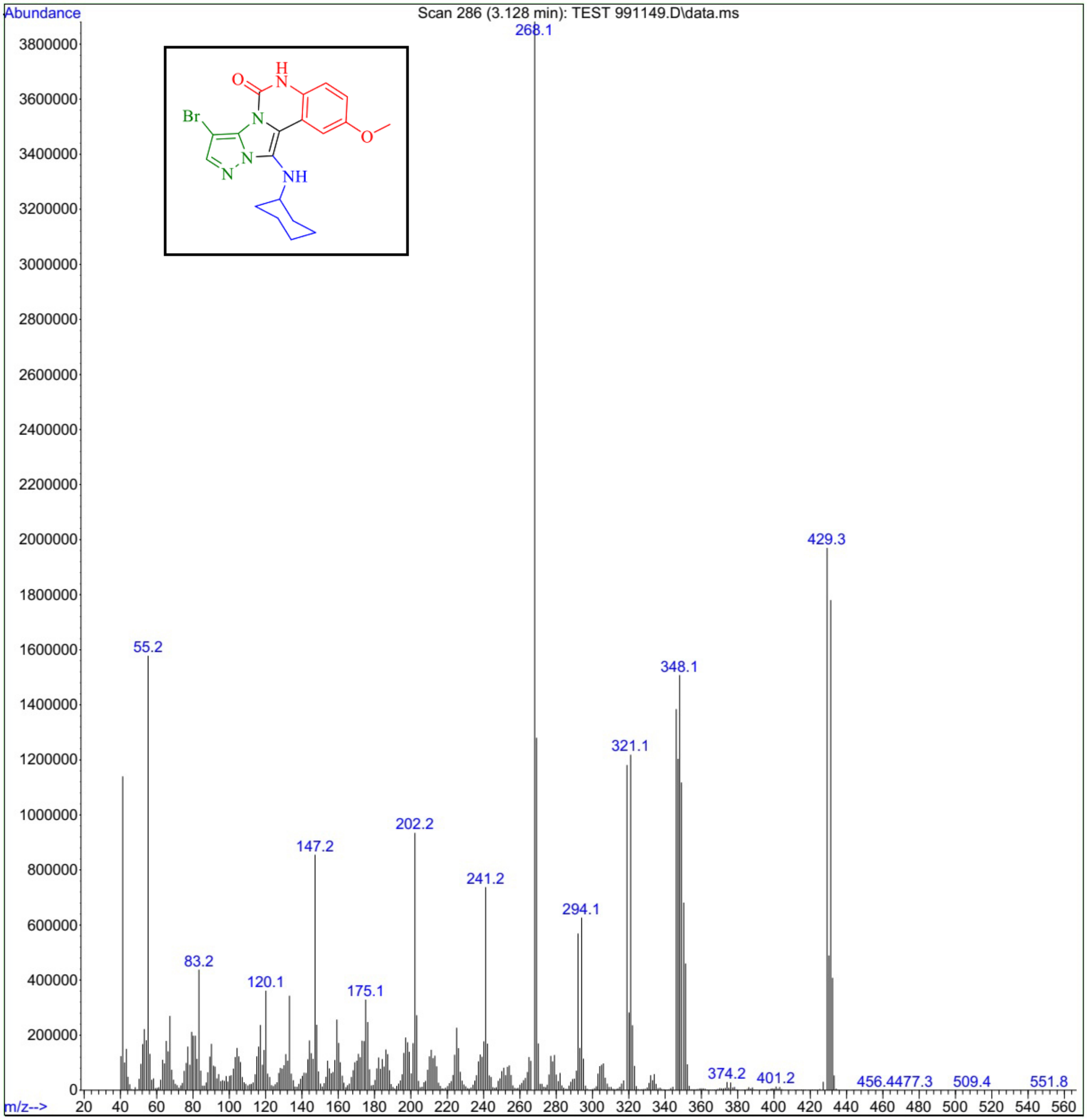

Figure S78. MASS spectrum of compound (4s).

^{1}H NMR, ^{13}C NMR, FT-IR and MASS of compound (4t)

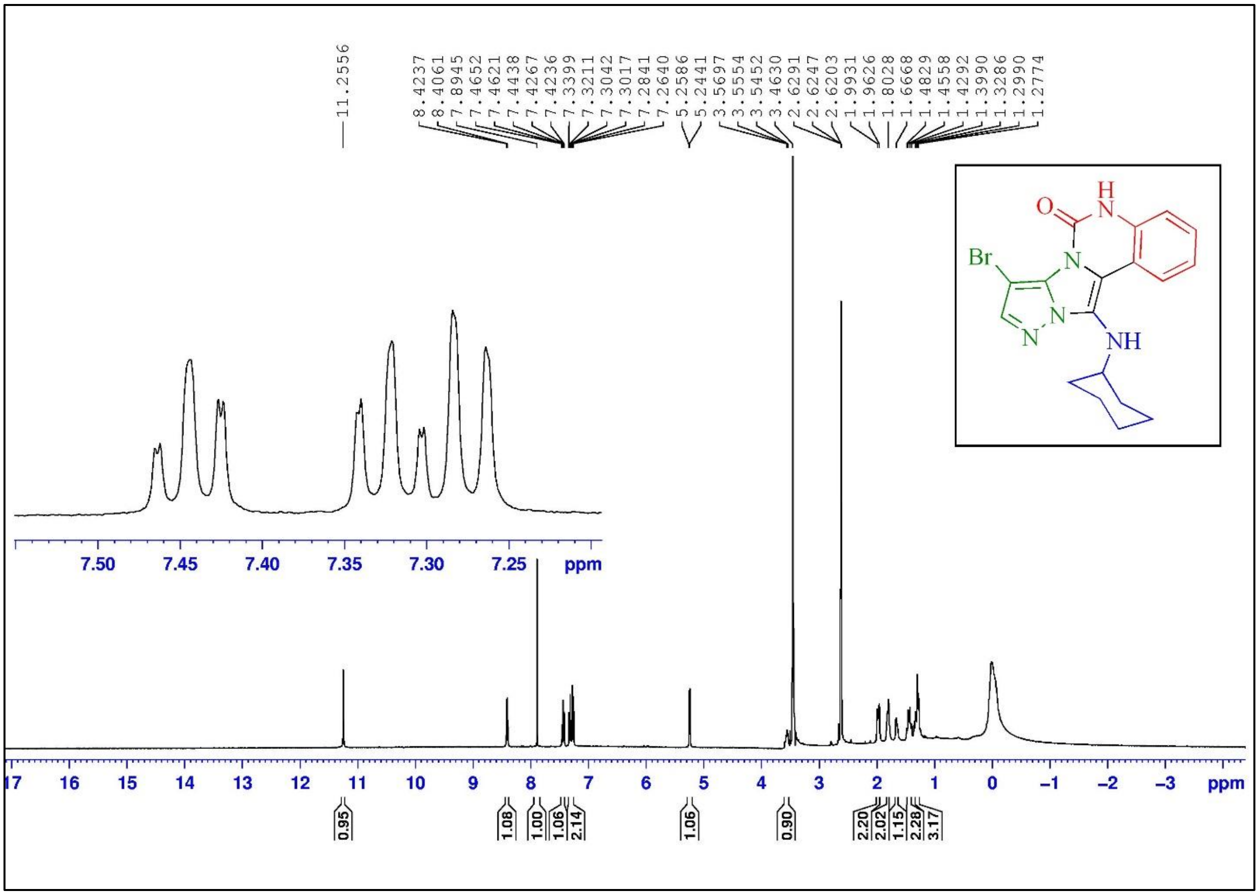

Figure S79. ^{1}H NMR spectrum of compound (4t) in DMSO.

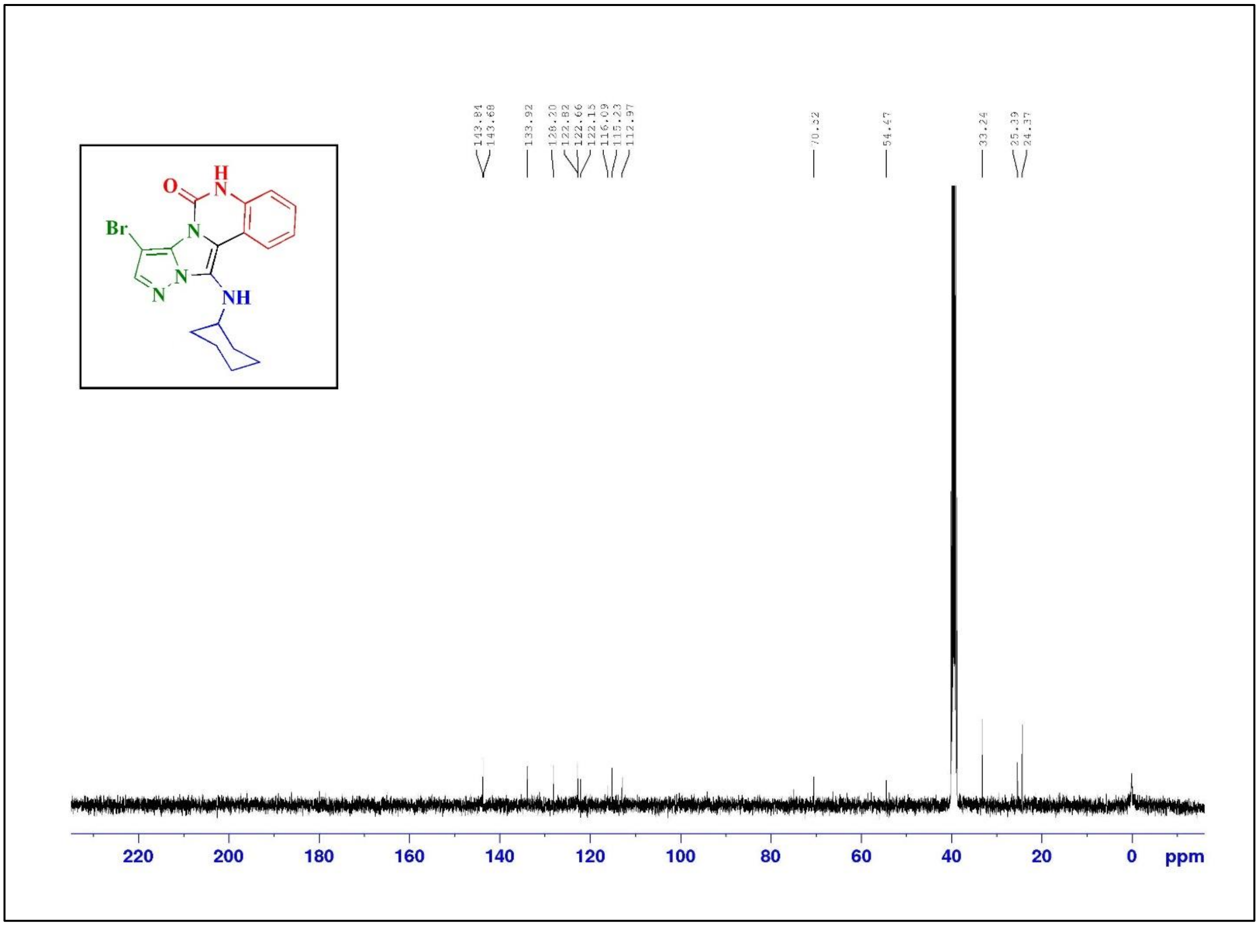

Figure S80. ^{13}C NMR spectrum of compound (4t) in DMSO.

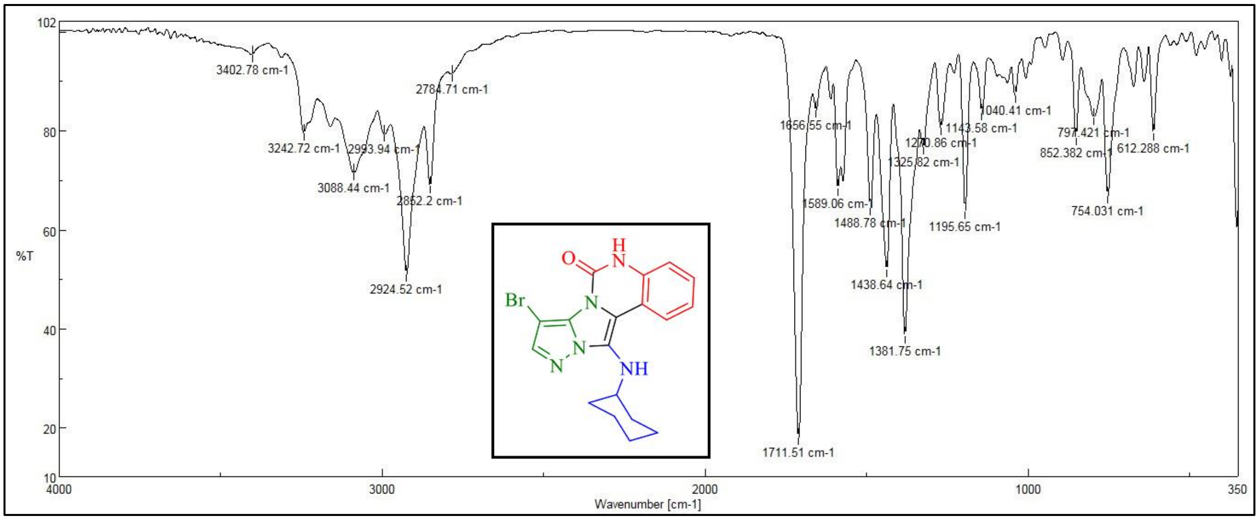

Figure S81. FT-IR spectrum of compound (4t).

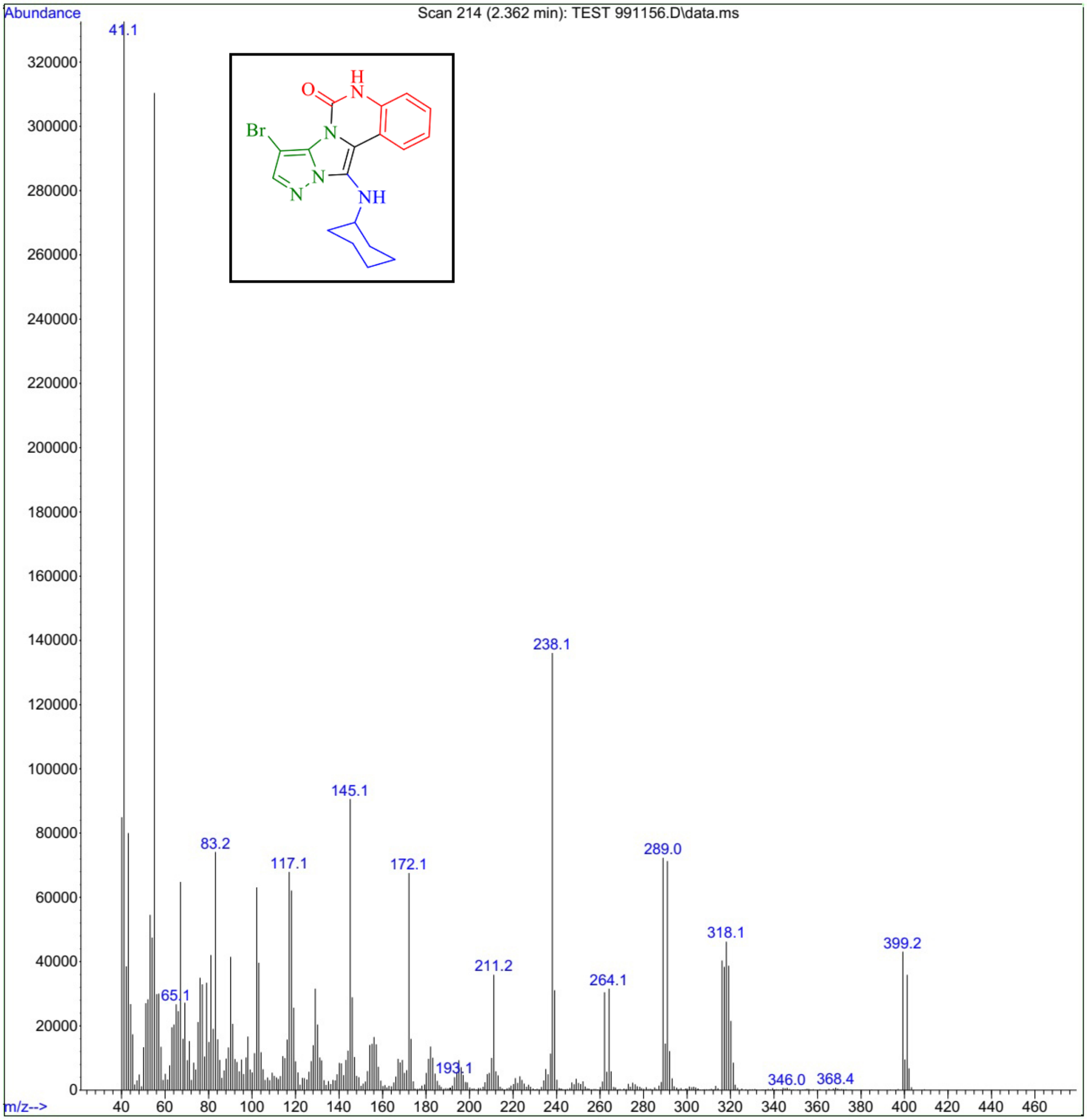

Figure S82. MASS spectrum of compound (4t).

^{1}H NMR, ^{13}C NMR, FT-IR and MASS of compound (4u)

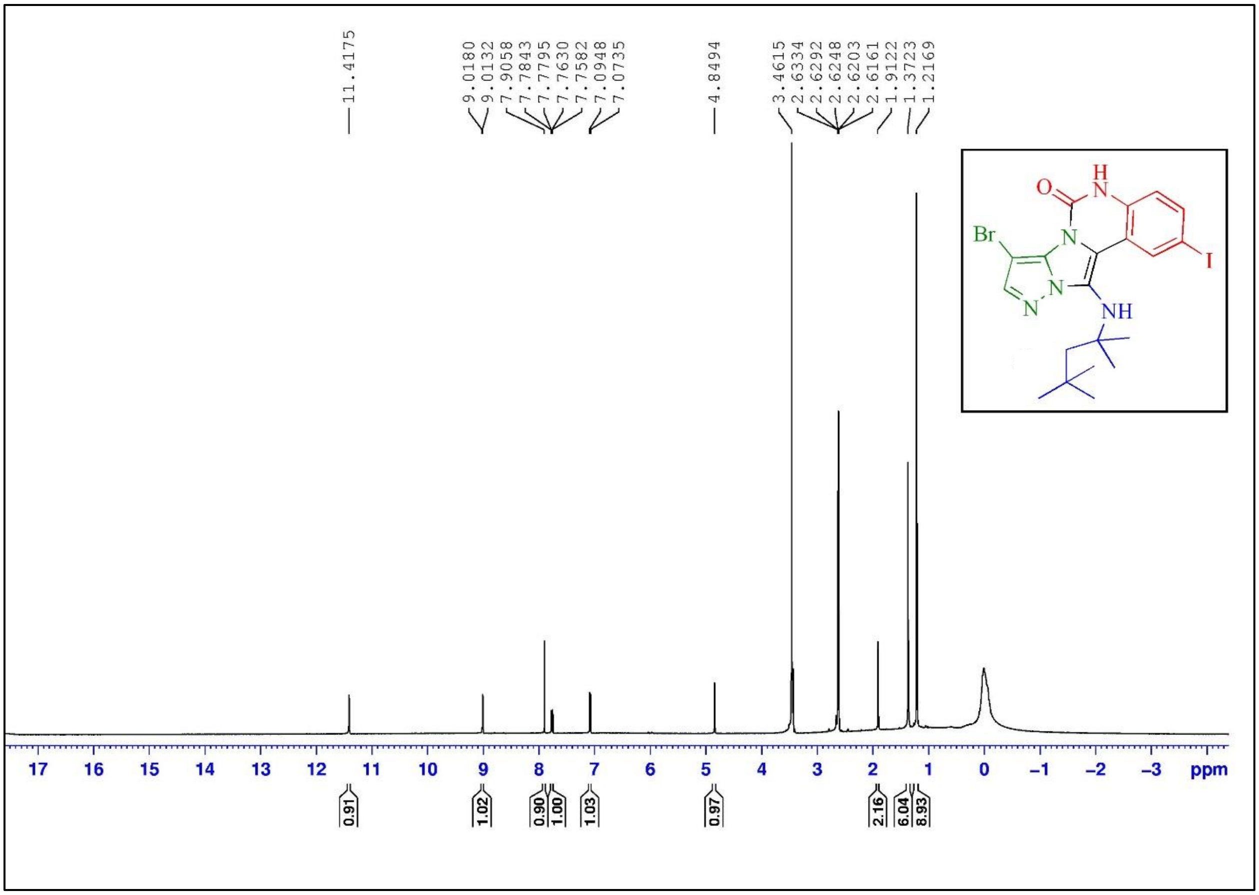

Figure S83. ^{1}H NMR spectrum of compound (4u) in DMSO.

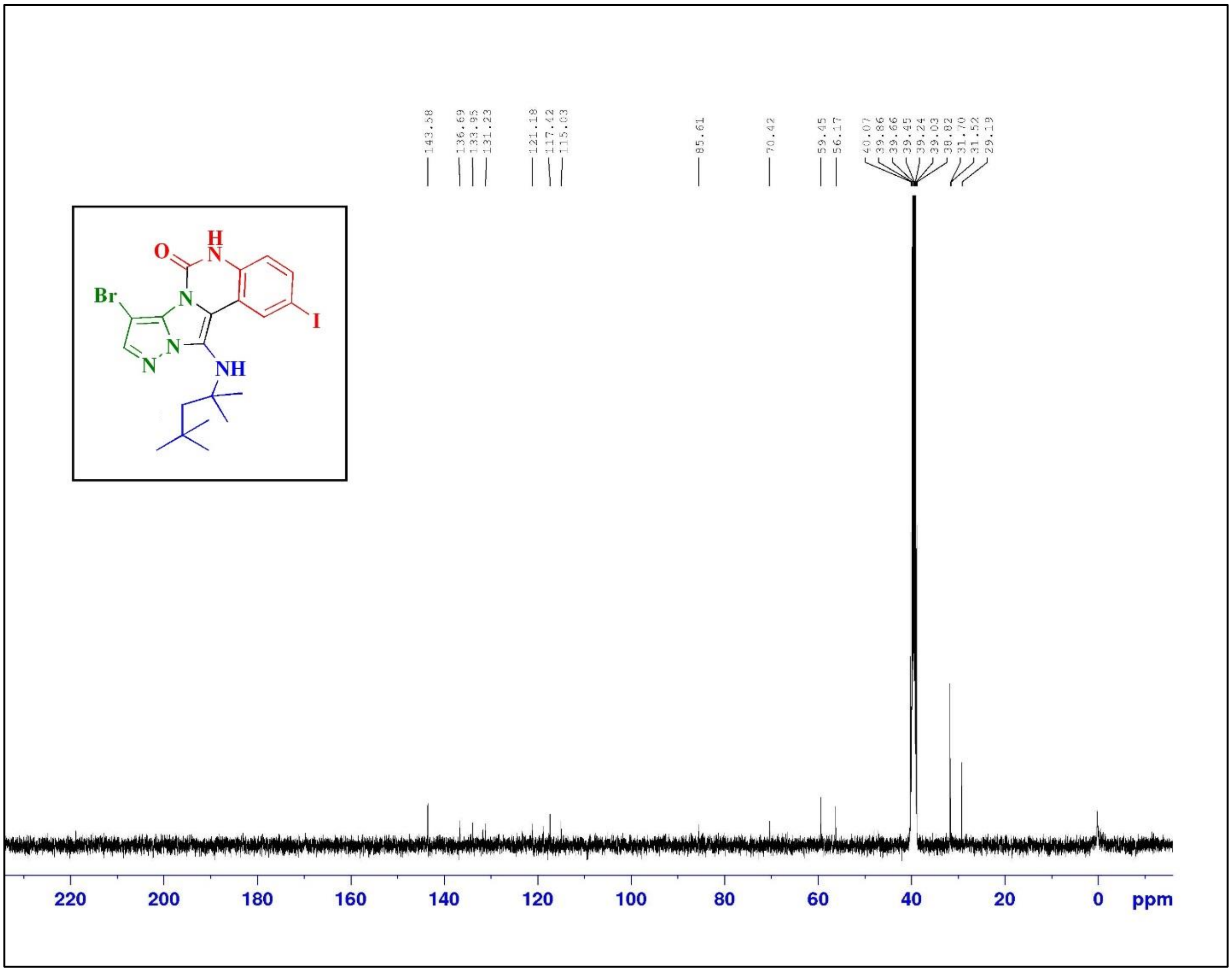

Figure S84. ^{13}C NMR spectrum of compound (4u) in DMSO.

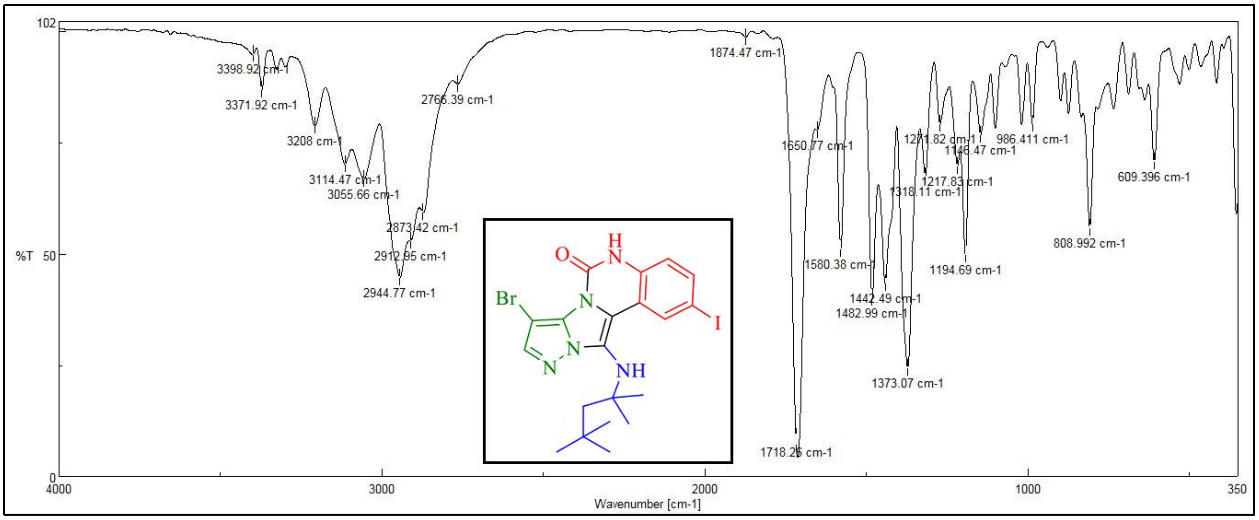

Figure S85. FT-IR spectrum of compound (4u).

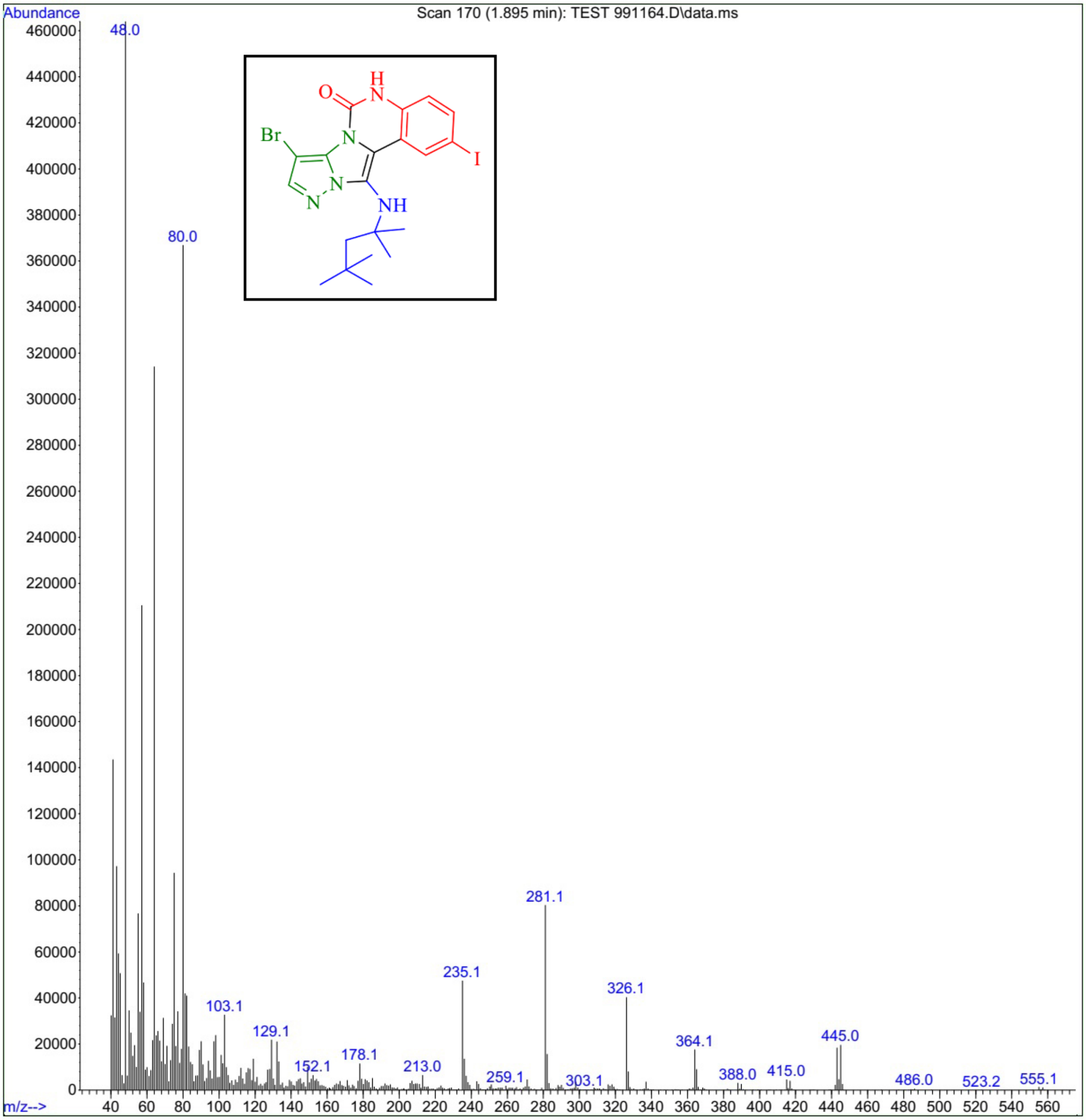

Figure S86. MASS spectrum of compound (4u).

Table S1. Molecular docking results for the interaction between Mpro and compounds 4a-4f.

Compound	Energy (kcal/mol)	Hydrogen Bond	Halogen Bond	Hydrophobic Interaction
4a	-8.30	THR26, ASN142, GLY143, CYS145	-	THR25, PHE140, GLU166, VAL309, LEU310
4b	-8.44	THR26, ASN142, GLY143, CYS145	-	THR25, PHE140, GLU166, VAL309, LEU310
4c	-8.26	THR26, ASN142, GLY143, CYS145	-	THR25, PHE140, GLU166, VAL309, LEU310
4d	-8.05	THR26, ASN142, GLY143, CYS145	-	THR25, PHE140, GLU166, VAL309, LEU310
4e	-8.13	THR26, ASN142, GLY143, CYS145	-	THR25, PHE140, GLU166, VAL309
4f	-8.22	SER144, HIS163, GLY143, CYS145	-	THR25, PHE140, GLU166, VAL309

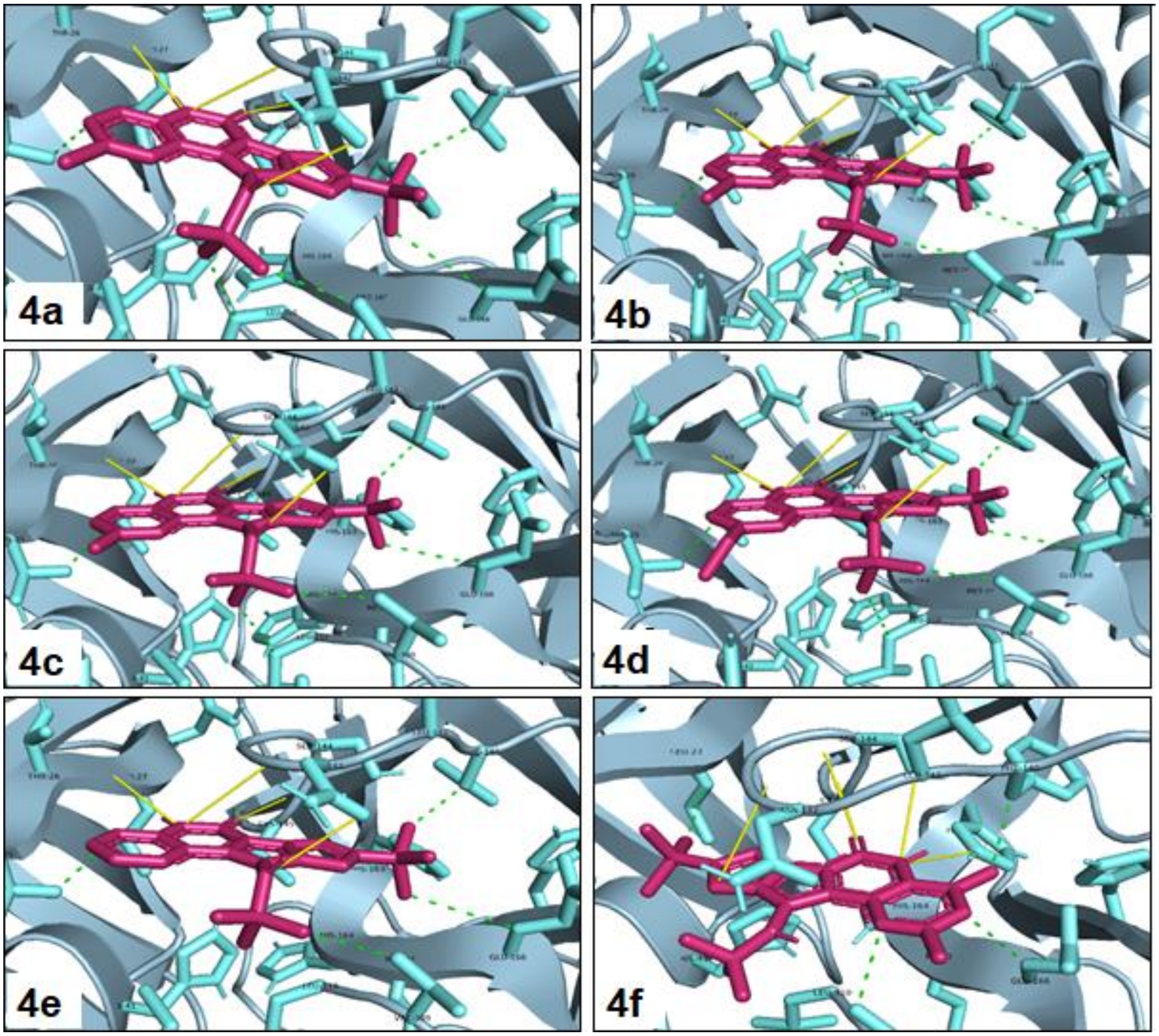

Figure S87. View of the Mpro binding site (Cys145-His41) for compounds 4a-4f. image of hydrophobic interaction, hydrogen and halogen bonds for ligands (yellow, green and blue color indication H-bond, hydrophobic interaction and halogen bond, respectively) that prepared by pymol software.

Table S2. Molecular docking results for the interaction between Mpro and compounds 4g-4k.

Compound	Energy (kcal/mol)	Hydrogen Bond	Halogen Bond	Hydrophobic Interaction
4g	-7.96	GLY143, SER144, CYS145	-	PHE140,GLU166
4h	-8.22	GLY143, SER144, CYS145	-	PHE140
4i	-7.90	ASN142,GLY143, SER144, CYS145	-	PHE140,GLU166
4j	-8.18	GLY143, SER144, CYS145	-	PHE140,GLU166
4k	-8.28	ASN142,GLY143, SER144, CYS145	-	PHE140

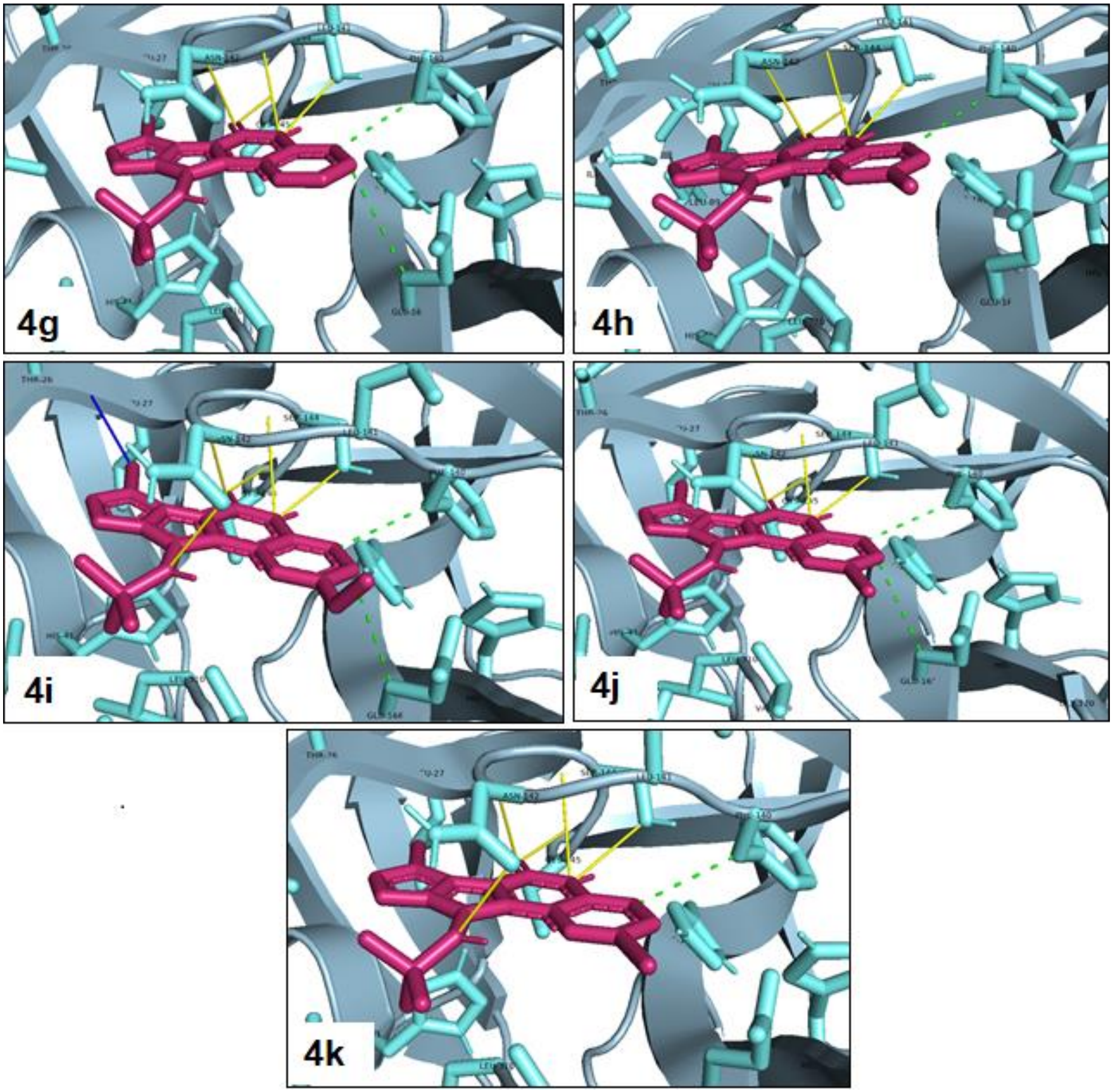

Figure S88. View of the Mpro binding site (Cys145-His41) for compounds 4g-4k. image of hydrophobic interaction, hydrogen and halogen bonds for ligands (yellow, green and blue color indication H-bond, hydrophobic interaction and halogen bond, Respectively) that prepared by pymol software.

Table S3. Molecular docking results for the interaction between Mpro and compounds 4l-4p.

Compound	Energy (kcal/mol)	Hydrogen Bond	Halogen Bond	Hydrophobic Interaction
4l	-8.51	SER144, ASN142, GLY143, CYS145	-	THR25, PHE140, GLU166
4m	**-8.63**	SER144, GLY143, CYS145	-	THR25, PHE140, GLU166
4n	-8.39	SER144, ASN142, GLY143, CYS145	-	THR25, PHE140, GLU166
4o	-8.41	HIS163, SER144, GLY143, CYS145	-	THR25, THR26, GLU166, PHE140
4p	-7.62	ASN142, GLY143	-	LEU27, PHE140, GLU166, VAL309

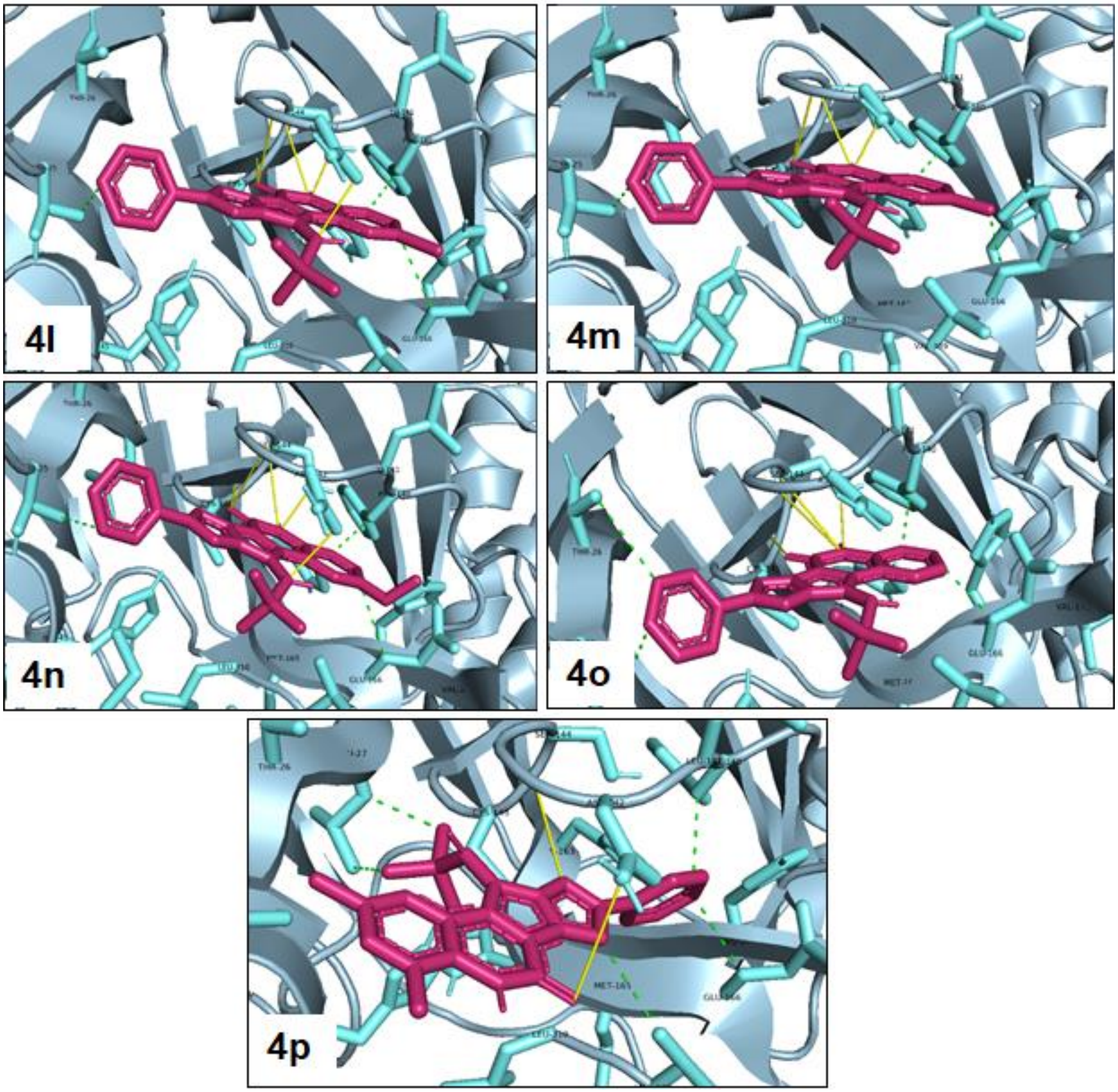

Figure S89. View of the Mpro binding site (Cys145-His41) for compounds 4l-4p. image of hydrophobic interaction, hydrogen and halogen bonds for ligands (yellow, green and blue color indication H-bond, hydrophobic interaction and halogen bond, Respectively) that prepared by pymol software.

Table S4. Molecular docking results for the interaction between Mpro and compounds 4q-4u.

Compound	Energy (kcal/mol)	Hydrogen Bond	Halogen Bond	Hydrophobic Interaction
4q	**-8.73**	ASN142,GLY143, SER144, CYS145	THR26	PHE140,GLU166
4r	**-8.77**	HIS163,GLY143, SER144, CYS145	THR26	PHE140,GLU166
4s	-8.51	ASN142,GLY143, SER144, CYS145	THR26	PHE140,GLU166
4t	-8.54	HIS163,GLY143, SER144, CYS145	THR26	PHE140,GLU166
4u	-8.22	ASN142,GLY143, SER144, CYS145	-	PHE140,GLU166,VAL309

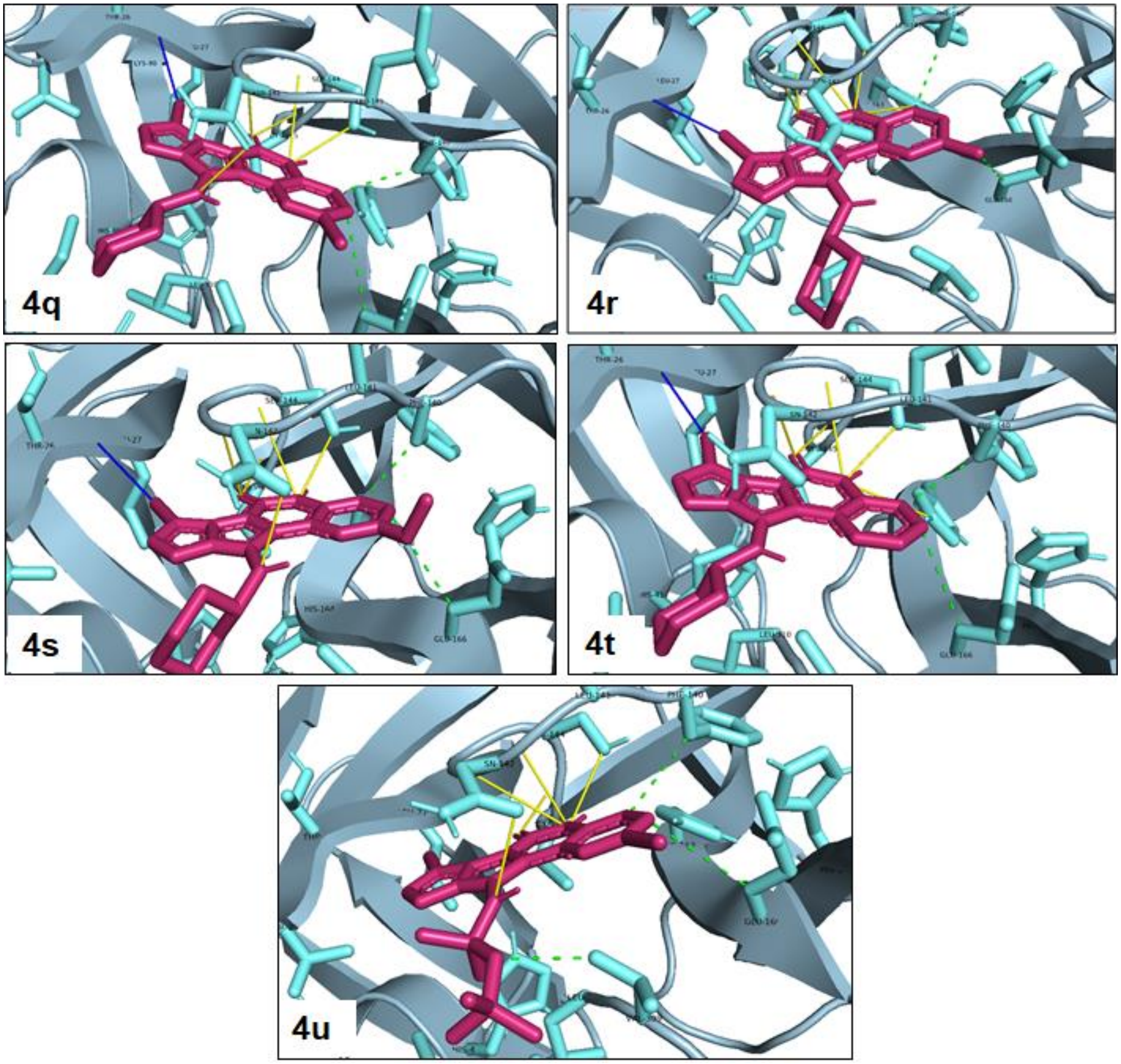

Figure S90. View of the Mpro binding site (Cys145-His41) for compounds 4q-4u. image of hydrophobic interaction, hydrogen and halogen bonds for ligands (yellow, green and blue color indication H-bond, hydrophobic interaction and halogen bond, Respectively) that prepared by pymol software.
